# In silico prediction of high-resolution Hi-C interaction matrices

**DOI:** 10.1038/s41467-019-13423-8

**Published:** 2019-12-06

**Authors:** Shilu Zhang, Deborah Chasman, Sara Knaack, Sushmita Roy

**Affiliations:** 1 Wisconsin Institute for Discovery, 330 North Orchard Street, Madison, WI 53715 USA; 2 Department of Biostatistics and Medical Informatics, University of Wisconsin-Madison, Madison, WI 53715 USA

**Keywords:** Computational models, Gene regulatory networks, Machine learning

## Abstract

The three-dimensional (3D) organization of the genome plays an important role in gene regulation bringing distal sequence elements in 3D proximity to genes hundreds of kilobases away. Hi-C is a powerful genome-wide technique to study 3D genome organization. Owing to experimental costs, high resolution Hi-C datasets are limited to a few cell lines. Computational prediction of Hi-C counts can offer a scalable and inexpensive approach to examine 3D genome organization across multiple cellular contexts. Here we present HiC-Reg, an approach to predict contact counts from one-dimensional regulatory signals. HiC-Reg predictions identify topologically associating domains and significant interactions that are enriched for CCCTC-binding factor (CTCF) bidirectional motifs and interactions identified from complementary sources. CTCF and chromatin marks, especially repressive and elongation marks, are most important for HiC-Reg’s predictive performance. Taken together, HiC-Reg provides a powerful framework to generate high-resolution profiles of contact counts that can be used to study individual locus level interactions and higher-order organizational units of the genome.

## Abstract (web summary)

Existing computational approaches to predict long-range regulatory interactions do not fully exploit high-resolution Hi-C datasets. Here the authors present a Random Forests regression-based approach to predict high-resolution Hi-C counts using one-dimensional regulatory genomic signals.

## Introduction

The three-dimensional (3D) organization of the genome has emerged as an important component of the gene regulation machinery that enables distal regulatory elements, such as enhancers, to control the expression of genes hundreds of kilobases away. These long-range interactions can have major roles in tissue-specific expression^1–3^ and how regulatory sequence variants impact complex phenotypes^4,5^, including diseases such as cancer, diabetes, and obesity^6,7^. Chromosome conformation capture (3C) technologies such as, 4C, 5C, ChIA-PET, Hi-C^8^, and Capture-Hi-C^9^, used to measure 3D proximity of genomic loci have rapidly matured over the past decade. However, there are several challenges for identifying such interactions in diverse cell types and contexts. First, the majority of these experimental technologies have been applied to well-studied cell lines. Second, very few Hi-C datasets are available at resolutions high enough (e.g., 5 kbp) to identify enhancer-gene interactions due to the tradeoff between measuring genome-wide chromosome organizations while achieving higher resolutions. Third, long-range gene regulation involves a complex interplay of transcription factors, histone marks and architectural proteins^10–13^, making it important to examine three-dimensional proximity in concert with these components of the transcription machinery.

Recently, numerous computational approaches for predicting long-range interactions have been developed^4,14–17^, which leverage the fact that regulatory regions that participate in long-range regulatory interactions have characteristic one-dimensional genomic signatures^14,15,18^. However, these methods have all used a binary classification framework, which is not optimal because the interaction prediction problem is a one-class problem with no negative examples of interactions. In addition, these do not exploit the genome-wide nature of high-throughput chromosome capture datasets such as Hi-C as they focus on significantly interacting pairs, which can be sensitive to the method used to call significant interactions. Recent comparison of methods for calling significant interactions show that there are substantial differences in the interactions identified from different methods^19^.

To overcome the limitations of a classification approach and to maximally exploit the information in high-throughput assays such Hi-C, we develop a Random Forests regression-based approach, HiC-Reg. HiC-Reg integrates published Hi-C datasets with one-dimensional regulatory genomic datasets such as chromatin marks, architectural and transcription factor proteins, and chromatin accessibility, to predict interaction counts between two genomic loci in a cell line-specific manner. We apply our approach to high-resolution Hi-C data from five cell lines to predict interaction counts at 5 kb resolution and systematically evaluate its ability to predict interactions within the same cell line (tested using cross-validation), across different chromosomes as well as different cell lines. Our work shows that a Random Forests-based regression framework can predict genome-wide Hi-C interaction matrices within cell lines and can generalize to new chromosomes and cell lines. Furthermore, modeling the signal between regions as well as integrating data from multiple cell lines is beneficial for improved predictive power. Feature analysis of the predicted interactions suggests that the CCCTC-binding factor (CTCF) and chromatin signals are both important for high-quality predictions. HiC-Reg predictions agree well with ChIA-PET datasets, recapitulate well-known examples of long-range interactions, exhibit bidirectional CTCF loops, and can recover topologically associating domains. Overall, HiC-Reg provides a computational approach for predicting the interaction counts discovered by Hi-C technology, which can be used for examining long-range interactions for individual loci as well as for global studies of chromosome conformation organization.

## Results

### HiC-Reg for predicting contact count using Random Forests

**Fig. 1:**
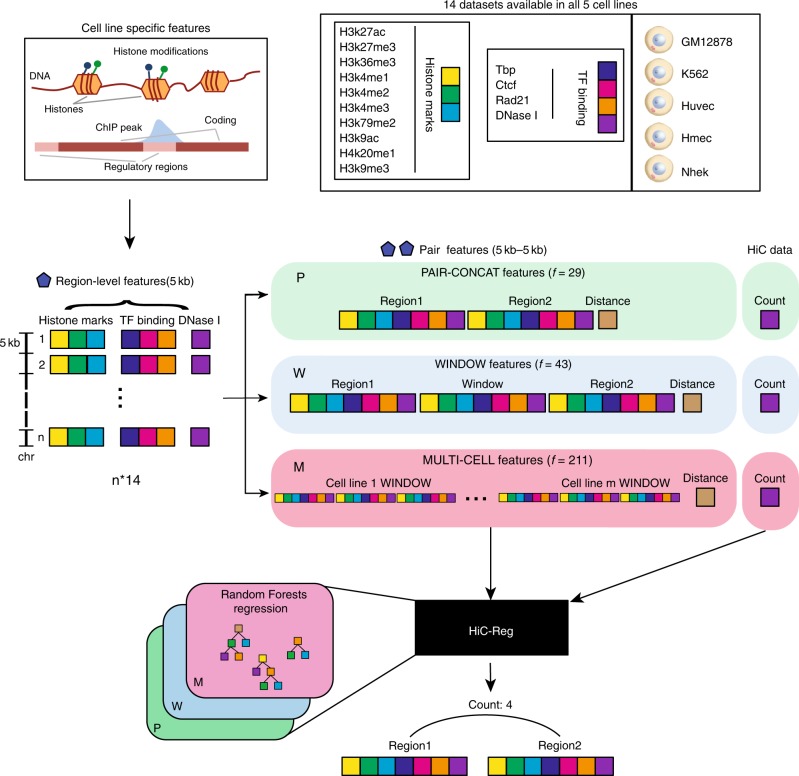
Overview of the HiC-Reg framework. HiC-Reg makes use of 14 datasets: ten chromatin marks and four datasets related to transcription factor (TF) binding. The TF-binding datasets are factor-specific ChIP-seq datasets and sequence-specific motif counts of a TF in accessible regions. A 5 kb genomic region is represented by a vector of aggregated signals of either chromatin marks, accessibility, or TF occupancy. A pair of regions in HiC-Reg is represented using one of three types of features: PAIR-CONCAT, WINDOW, and MULTI-CELL. HiC-Reg uses Random Forests regression to predict the contact counts between pairs of genomic regions. Once trained, HiC-Reg takes as input a feature vector for a pair of regions and gives as output a predicted contact count for that pair (e.g., Count: 4).

We developed a regression-based approach called HiC-Reg to predict cell line-specific contact counts between pairs of 5 kb genomic regions using cell line-specific one-dimensional regulatory signals, such as histone marks and transcription factor-binding profiles (Fig. 1). Our regression framework treats contact counts as outputs of a regression model from input one-dimensional regulatory signals associated with a pair of genomic regions. As cell line-specific training count data we used high-resolution (5 kb) Hi-C datasets from five cell lines from Rao et al.^20^. We used features from 14 cell line-specific regulatory genomic datasets and genomic distance to represent a pair of regions. These datasets include the architectural protein CTCF, repressive marks (H3k27me3, H3k9me3), marks associated with active gene bodies and elongation (H3k36me3, H4k20me1, H3k79me2), enhancer-specific marks (H3k4me1, H3k27ac), activating marks (H3k9ac, H3k4me2, H3k4me3), cohesin component (RAD21), a general transcription factor (TBP) and DNase I (open chromatin)^14^. HiC-Reg uses a Random Forests regression model as its core predictive model. The Random Forests regression model significantly outperforms a linear regression model, suggesting that there are non-linear dependencies that are important to be captured to effectively solve the count prediction problem (Supplementary Fig. 1).

**Fig. 2:**
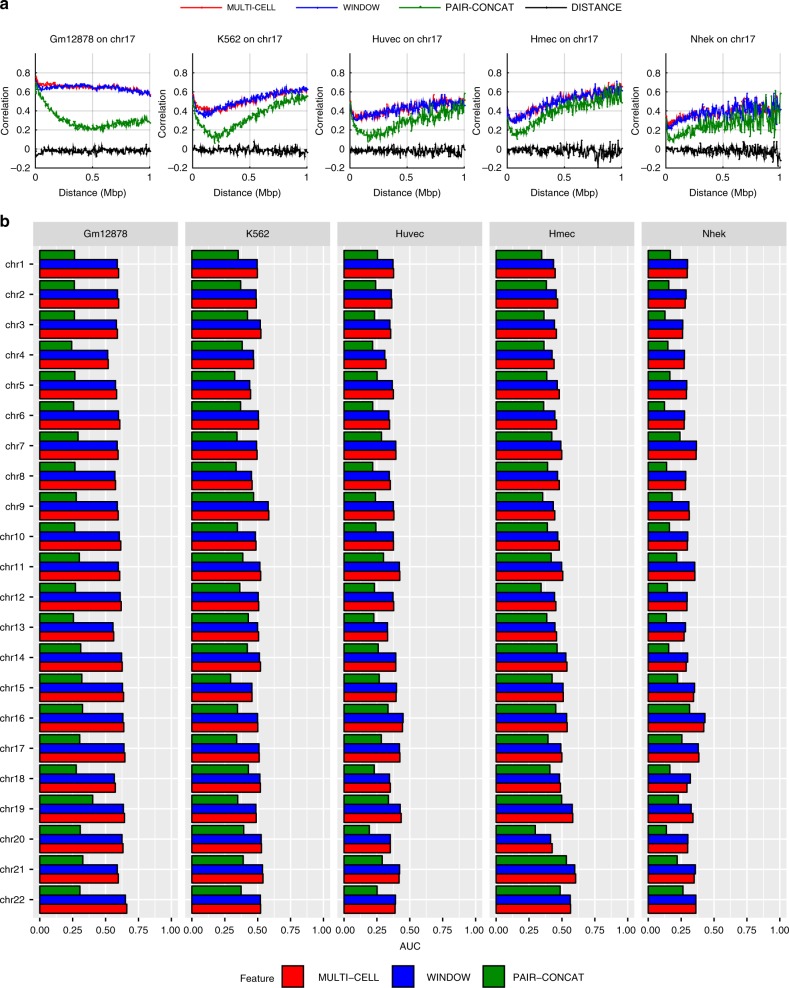
HiC-Reg cross-validation performance. **a** The distance-stratified Pearson’s correlation plots of test data when training on the same cell line for chromosome 17 in five cell lines: Gm12878, K562, Huvec, Hmec, Nhek. The *x*-axis corresponds to a particular distance bin and the *y*-axis corresponds to the Pearson’s correlation of the counts predicted by HiC-Reg for pairs at a particular distance and the true counts. The correlation plots for three types of feature representations of pairs are shown: MULTI-CELL, WINDOW, and PAIR-CONCAT. **b** Area under the curve (AUC) for the distance-stratified Pearson’s correlation in all chromosomes for each of the five cell lines. The AUC is computed for test pairs using fivefold cross-validation. Source data are provided as a Source Data file.

A key consideration for chromosomal count prediction is how to represent the genomic region pairs as examples for training a regression model. To represent a pair in HiC-Reg, we considered three main feature encodings in addition to genomic distance between the two regions of interest: PAIR-CONCAT, WINDOW, and MULTI-CELL (Fig. 1). PAIR-CONCAT simply concatenates the features for each region into a single vector. The WINDOW feature encoding additionally uses the average signal between two regions and was proposed in TargetFinder, a classification-based method^15^. MULTI-CELL incorporates signals from other cell lines. We assessed the performance of these methods using distance-stratified Pearson’s correlation computed on test set pairs in a fivefold cross-validation setting (Methods). The distance-stratified correlation measures the Pearson’s correlation between predicted and true counts for genomic pairs at a particular distance threshold. Distance stratification is important due to the high dependence of contact count on genomic distance^21^. As a baseline we compared the performance of these models to a Random Forests regression model trained on distance alone. The performance of HiC-Reg is much better than the performance of distance alone, demonstrating that addition of regulatory signals significantly improves performance (Fig. 2). Between PAIR-CONCAT and WINDOW, WINDOW features are significantly better and the performance of PAIR-CONCAT often decreases as a function of distance especially for the first 250 kb (Fig. 2a). WINDOW and MULTI-CELL have similar performance and are both better than PAIR-CONCAT.

To examine the generality of this behavior across all chromosomes, we applied HiC-Reg in all chromosomes in a fivefold cross-validation setting. To summarize the performance captured in the distance-stratified correlation curve, we computed the area under the distance-stratified correlation curve (AUC, Methods). The AUC metric ranges from –1 to 1, with 1 representing the best performance. We find that across all cell lines and chromosomes, both the WINDOW and the MULTI-CELL features are significantly better than PAIR-CONCAT, suggesting that incorporating the signal between two regions is important for capturing these interaction counts at longer distances (Fig. 2b). When examining the performance across the five cell lines, the AUC for the Nhek cell line was lowest, suggesting it is the hardest to predict. Owing to the superior performance of the WINDOW and MULTI-CELL features, we excluded the PAIR-CONCAT feature from subsequent experiments. Although WINDOW has more features, the number of datasets needed are still comparable to PAIR-CONCAT.

**Fig. 3:**
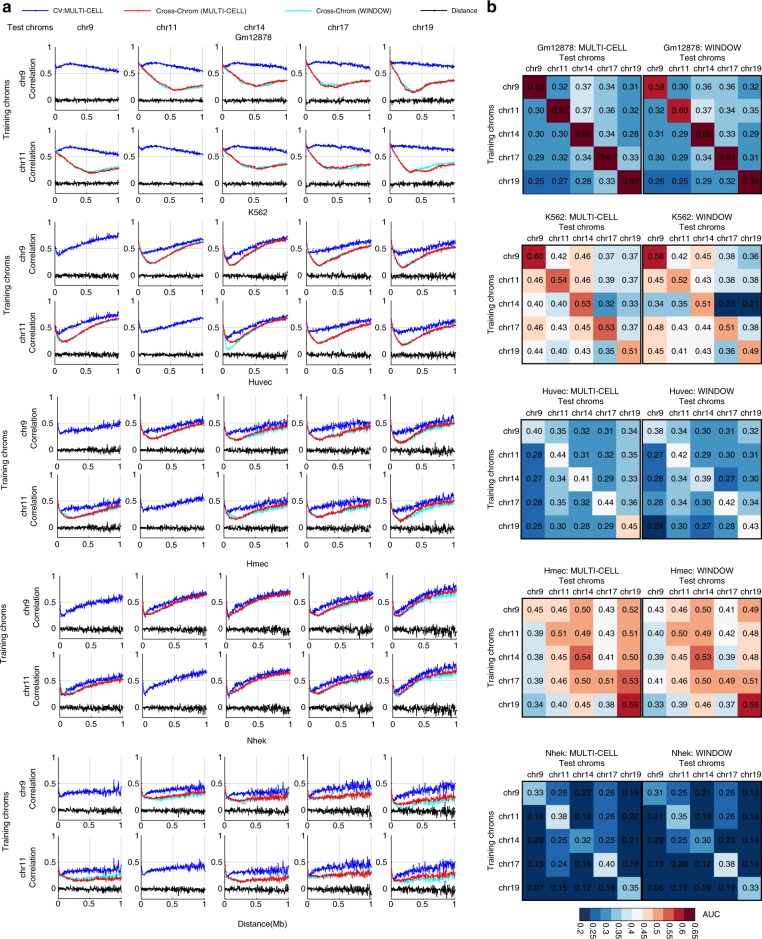
HiC-Reg cross-chromosome performance. **a** The distance-stratified Pearson’s correlation plot when training on one chromosome and testing on a different chromosome from the same cell line. Shown are the distance-stratified correlations for models trained on chromosome 9 and 11 (row groups) and tested on chromosome 9, 11, 14, 17, 19 (columns) at 5 kb resolution in different cell lines. Each pair of rows corresponds to a particular cell line and results are shown for five cell lines: Gm12878, K562, Huvec, Hmec, Nhek. The blue line refers to training and testing on the same chromosome in cross-validation mode. **b** Heatmap of AUC for all pairs of tested cross-chromosome experiments. Each off-diagonal entry in the heatmap denotes the AUC when trained on the row chromosome and tested on the column chromosome. The more red an entry the better the performance in that chromosome pair combination. The diagonal entries are the AUC values when training and testing on the same chromosome in cross-validation mode. Source data are provided as a Source Data file.

To examine whether our approach can generalize to new chromosomes, we trained HiC-Reg on one chromosome and used it to predict counts in a different chromosome for the same cell line. We considered five chromosomes, chr9, chr11, chr14, chr17, and chr19, each as training and test chromosomes, in all five cell lines. HiC-Reg predictions across chromosomes were slightly worse than when training and testing on the same chromosome in all but the Gm12878 cell line, where the cross chromosome performance was much worse. Interestingly, while both feature types, WINDOW and MULTI-CELL were comparable when training and testing in the same chromosome setting (Fig. 2), when comparing models across chromosomes, we found that the MULTI-CELL feature was significantly better than the WINDOW feature in the cell lines with low depth (Nhek, Hmec, Huvec, *t*-test one-sided *p*-value $< \, 0.05$), and comparable in the more deeply sequenced cell lines (Gm12878, K562, Fig. 3 and Supplementary Figs. 2–6). In all cases, MULTI-CELL outperformed WINDOW features more times than it was outperformed (Fig. 3). Some chromosomes produced worse models than other chromosomes. For example, chromosome 19 was a poor predictor of other chromosomes in all cell lines, and chromosome 14 was a poor predictor of all other chromosomes in K562. These performance differences are indicative of unique chromosomal features that are shared or unique to specific cell lines (chr14 in K562). Taken together, our analysis showed that HiC-Reg is able to successfully predict interaction counts in different cell lines using one-dimensional features and the MULTI-CELL feature is especially useful for cell lines with low-sequencing depth.

### Identifying key determinants of contact count prediction

**Fig. 4:**
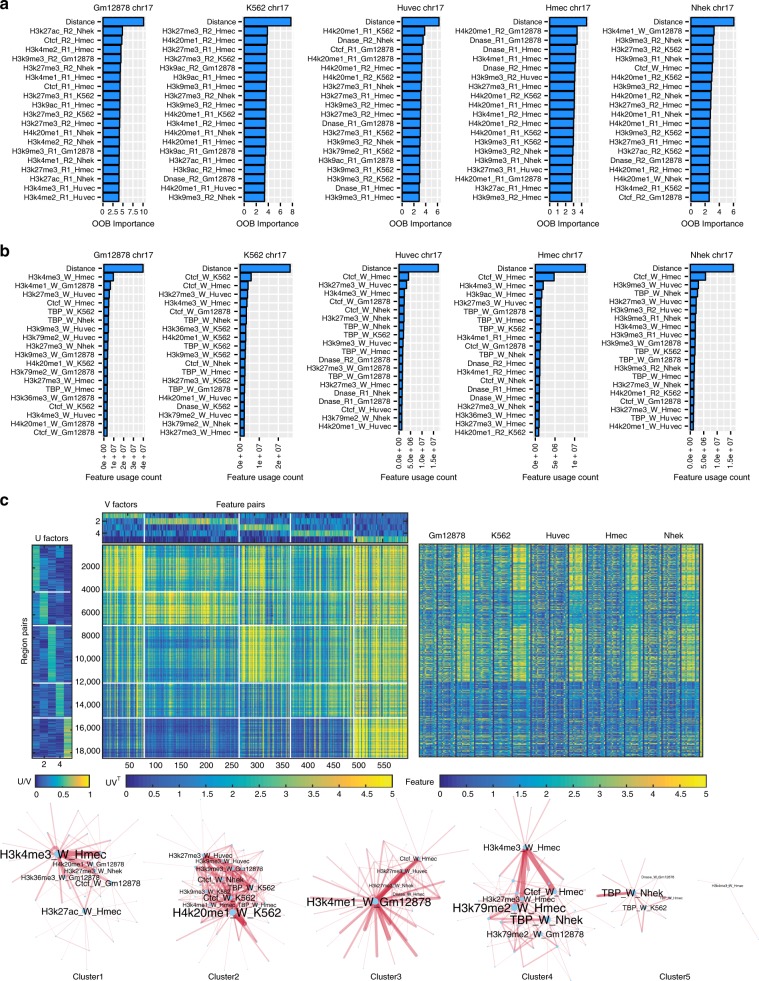
Analysis of features important for predicting Hi-C contact counts. **a** Shown are the top 20 MULTI-CELL features ranked based on Out of Bag (OOB) feature importance on chromosome 17 in all five cell lines. Each horizontal bar corresponds to one feature. The feature name includes the name of the histone mark, DNase I or TF, whether it is on one of the interaction regions (R1, R2) or in the intervening window (W), and the specific cell line from which this feature is extracted. **b** Shown are top 20 features ranked based on counting the number of times a feature is used for test set predictions. Feature rankings are for chromosome 17 for all five cell lines. **c** Non-negative matrix (NMF) factorization of region-pair by feature-pair matrix for Gm12878 chromosome 17. The ${\bf{U}}$ and ${\bf{V}}$ factors are the NMF factors to provide membership of region pairs or feature pairs in a cluster (white lines demarcate the region pair and feature pair clusters). The factorized feature count matrix is shown below the ${\bf{V}}$ factors and to the right of the ${\bf{U}}$ factors. The heatmap on the right are the features associated with each of the pairs, with rows corresponding to a pair of regions and columns corresponding to the feature values grouped by the cell line from which they are obtained. Bottom are Cytoscape network representation of important pairs of features. The node size is proportional to the number of times the specific feature co-occurs on a path in the regression tree. The thickness of the line is proportional to the number of times the pair of features is used on the path from root to the leaf for a test example pair. Font size of the node label is proportional to its size.

To gain insight into the relative importance of different one-dimensional regulatory signals, such as chromatin marks and transcription factor-binding signals for predicting contact counts, we conducted different types of feature analyses. We focused on the MULTI-CELL features as they had the best performance. We first ranked features using a standard feature importance score, Out of Bag (OOB) error (Methods, Fig. 4a and Supplementary Fig. 7). For a given cell line, the features from the cell line as well as features from the other four cell lines were identified as important (e.g., several Nhek, K562, Hmec, and Huvec features were important for the Gm12878 cell lines, Fig. 4a). The feature rankings across different chromosomes were similar (Spearman correlation 0.59–0.78, Supplementary Fig. 7D). Based on this ranking, the most important features included Distance, an elongation chromatin mark H4K20me1, DNase I, repressive marks H3k27me3 and H3K9me3, activating marks H3k9ac, and enhancer-associated marks H3K4me1 and H3k27ac, typically on one or both end point regions (R1 and R2) and rarely on the Window regions (W). We next used a complementary strategy of counting the number of times a feature was used for predicting the count of a test pair (Fig. 4b and Supplementary Fig. 8). This analysis also found Distance to be important but also other factors like CTCF, TBP, and other elongation marks (H3k36me3 and H3k79me2), and promoter activating marks (H3k4me3). Here too the features from both the training cell line and other four cell lines were important. Comparing across chromosomes, the feature rankings were very similar (Spearman correlation 0.89–0.97, Supplementary Fig. 8D). The two feature analysis methods agreed on the importance of elongation mark, H4K20me1, repressive marks H3K27me3 and H3k9me3, enhancer marks H3K4me1, DNase I, and CTCF, but there were some differences. The OOB method identified histone mark features in the region (R1 and R2) while the feature usage counting importance implicated CTCF and TBP Window signals to be the most important.

While the above approach identified the top features for all pairs of regions, it does not tell us whether there are different feature sets useful for different sets of pairs. In particular, some interacting pairs could be driven largely by chromatin marks, while another set of pairs could be driven by transcription factors. Furthermore, it does not inform us about dependencies among features that might be important for making these predictions. Therefore, we developed a novel feature analysis method, based on non-negative matrix factorization (NMF, Methods). Briefly, we obtained region pairs with the lowest 5% test error and counted the number of times a feature or a pair of features was used on the tree path traversed for these region pairs. Restricting to the pairs with the 5% lowest errors did not change individual feature rankings and was computational more tractable (Supplementary Figs. 9 and 10). We focused on chromosome 17 because the feature importances were similar across chromosomes (Supplementary Figs. 7 and 8). We created a region-pair by feature-pair matrix, with each entry of the matrix denoting the number of times the feature pair was used in the trees. We next applied NMF on this matrix to obtain clusters of region pairs associated with clusters of feature pairs (Fig. 4c). Such bi-clusters are indicative of different classes of region pairs and the most important features associated with them (Fig. 4c). For example, for Gm12878, one cluster (Cluster 1) was associated with H3K4me3 and H3k27ac, while another cluster (Cluster 2) was associated with elongation marks (H4K20me1) and CTCF together with other histone marks. A third cluster (Cluster 3) was associated with H3k4me1 in Gm12878, CTCF from Hmec and H3k27me3 from Huvec and Nhek. Cluster 4 was associated with H3k79me2 and CTCF in Hmec and TBP in Nhek and finally Cluster 5 was largely associated with TBP. We found similar behavior in other cell lines as well (Supplementary Figs. 11–14). While CTCF was important in all cell lines, some cell lines exhibited other types of important features like H3k9me3 (Nhek), DNase I (Huvec and Hmec) and H3k4me1 (Hmec). We applied the same procedure to the matrix of region-pairs by individual features (Supplementary Figs. 15 and 16), however, the groups were essentially driven by the type of region (R1, R2, or W). While this showed that NMF captures an important grouping structure, it was not unexpected and only served as a sanity check. Our feature analysis showed that there were largely CTCF-driven and chromatin mark driven clusters of interactions. CTCF was a key feature for predicting interactions and was associated with other chromatin marks. Furthermore, we found that elongation marks like H4K20me1 and H3K79me2 and repressive marks like H3K27me3 were often important as feature hubs.

As all 14 genomic datasets might be expensive to measure in a new context (e.g., a cell line or tissue), we next asked if we can reduce the number of datasets needed for HiC-Reg without substantial loss in performance. We used a two-step approach (MTG-RF) to select a minimal feature set (Methods), similar to a previous approach applied in a classification setting^14^. First, we applied a multi-task regression framework, based on Multi-task Group LASSO (MTG) to select a small number of features that were important for all five cell lines. Datasets spanning these features (e.g., from one or both regions) were used as input in the second step, which we iteratively refined using Random Forests (RF). We applied this approach on different subsamples of the data and generated a ranking of datasets based on the number of times it is selected as a contributor to an important feature. We averaged the rankings from all five cell lines and selected top six and eight datasets (Supplementary Fig. 17A). In addition to Distance, the top six datasets included CTCF, DNase I, H4K20me1, RAD21, TBP, and H3K9me3, while the top eight datasets additionally included H3K4me1 and H3K79me2.

We next compared HiC-Reg trained on these reduced datasets against HiC-Reg trained on all 14 datasets in a cross-validation (Supplementary Fig. 17B, C) and cross chromosome setting (Supplementary Fig. 18). In the cross-validation setting, models trained on the top six and eight datasets have a slightly diminished performance compared to using all 14 datasets (Supplementary Fig. 17B, C), which is expected. In the cross-chromosome experiments from Gm12878 (Supplementary Fig. 18), a model using the top six datasets has a diminished performance while a model using the top eight datasets is comparable to the full 14 genomic datasets models. For other cell lines, the top six and eight datasets models are able to perform comparably as the full 14 datasets, with top eight being slightly better than the top six datasets model.

### Predictions show hallmarks of true loops and identify TADs

**Fig. 5:**
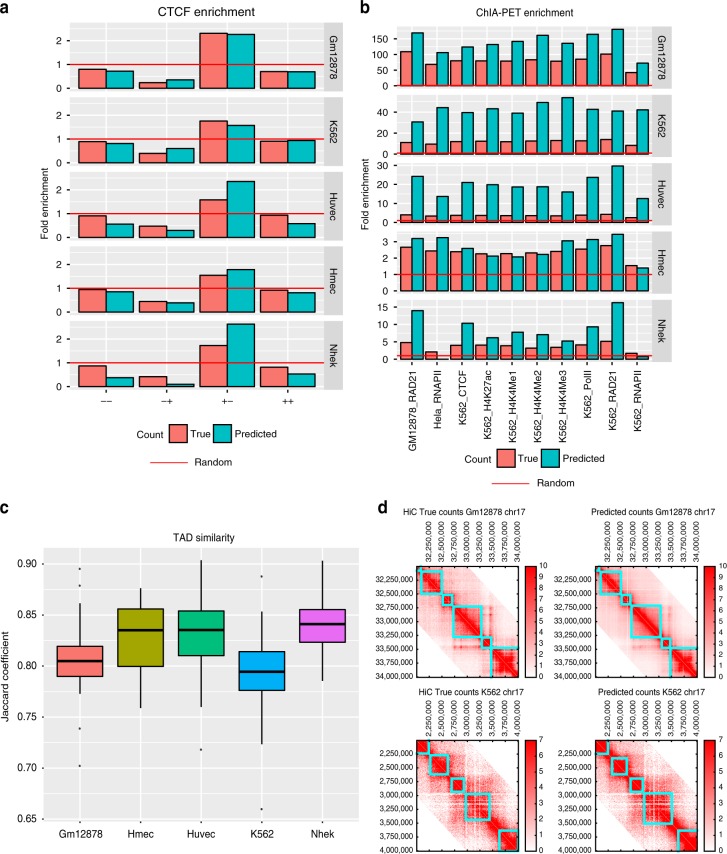
Assessing significant interactions and TADs from HiC-Reg predictions. **a** Fold enrichment of four configurations of CTCF motifs in significant interactions identified using true and predicted counts. A fold enrichment $$> $$1 (red horizontal line) is considered as enriched. Fold enrichment in all five cell lines is shown. **b** Fold enrichment of interactions identified using ChIA-PET experiments in significant interactions from HiC-Reg’s predictions and true counts. A fold enrichment $$> $$1 (red horizontal line) is considered as enriched. **c** Distribution of TAD similarity identified from true and predicted counts for all chromosomes. Each point in the box plot corresponds to the average Jaccard coefficient for a chromosome. The horizontal middle line of each plot is the median. The bounds of the box are 0.25 quantile ($${Q}_{1}$$) and 0.75 quantile ($${Q}_{3}$$). The upper whisker is the minimum of the maximum value and $${Q}_{3}+1.5* IQR$$, where $$IQR={Q}_{3}-{Q}_{1}$$. The lower whisker is the maximum of the minimum value and $${Q}_{1}-1.5* IQR$$. **d** TADs identified on true (left) and predicted (right) HiC count matrices for selected regions. Top: Gm12878 cell line, chr17:32–34 Mbp. Bottom: K562 cell line, chr17:2–4 Mbp). Source data are provided as a Source Data file.

We next assessed the quality of our predictions using several additional metrics based on specific properties of true interactions. One such property is the occurrence of bidirectional CTCF motifs^20^. Briefly, a pair can have one of four configurations of the CTCF motif, (+ +), (+ –), (– +), and (– –), where + corresponds to the motif on the forward strand and – corresponds to the motif being on the reverse strand. The pairs with the (+ –) configuration are most likely the true loops. This property of looping interactions was also used by Forcato et al.^19^ to compare different Hi-C peak-calling programs. To assess the occurrence of CTCF bidirectional motifs in HiC-Reg predictions, we identified significant interactions in both true and predicted counts (Methods). We used Fit-Hi-C to call significant interactions, however, there is substantial overlap among interactions when using another interaction caller (Supplementary Tables  1 and 2). Following Forcato et al.^19^, we only focus on pairs with at least one CTCF signed motif mapped to each pair of regions, but discarding a pair if one of the regions has the motif in both orientations. We quantified the tendency of CTCF bidirectional motifs to occur in the significant pairs versus all pairs using fold enrichment. Across all five cell lines, significant pairs called on both the true and predicted counts are enriched for the bidirectional motif (+ –) configuration (Fig. 5a). The level of enrichment is comparable for the interactions identified from the true and predicted counts and is sometimes better in the predicted counts. The majority of significant interactions identified using the predicted counts are identified from the true counts, although the number of interactions from true counts are higher (Supplementary Table 3). This suggests that the predicted interactions have high precision and are likely true positives. Furthermore, there is significant overlap between the top 5% pairs when ranked based on Fit-Hi-C *q*-values on the predicted or true counts (Supplementary Table 4).

As a second evaluation metric, we compared the significant interactions identified by Fit-Hi-C on the predicted and true counts with interactions identified using a complementary experiment, ChIA-PET (Methods). We obtained ten published datasets for different factors (RNA PolII, CTCF, and RAD21) and histone marks in multiple cell lines^22,23^. We estimated fold enrichment of the ChIA-PET interactions in the significant interactions compared to background. Interactions from both true counts and HiC-Reg predictions were enriched for ChIA-PET interactions, and these enrichments were often better for HiC-Reg predictions than those from the true counts (Fig. 5b). The CTCF directionality and ChIA-PET enrichment suggest that significant interactions from HiC-Reg predictions exhibit hallmarks of true looping interactions.

One of the advantages of a regression versus a classification framework is that the output counts can be examined with topologically associating domains (TAD) finding algorithms^19,24^. Hence, as a third validation metric, we asked to what extent the HiC-Reg counts identify structural units of chromosomal organization, such as TADs^25^. We applied the Directionality Index (DI) Domain Caller method^26^ to HiC-Reg predicted counts and true counts and compared the similarity of the identified TADs using a metric derived from the Jaccard coefficient. The Jaccard coefficient assesses the overlap between two sets of objects (e.g., regions in one TAD versus regions in another TAD) and is a number between 0 and 1, with 0 representing no overlap and 1 representing perfect overlap. We aggregated the Jaccard coefficient across all TADs identified on a chromosome into a single average Jaccard coefficient (Methods). Across all cell lines and chromosomes, the average Jaccard coefficient ranged between 0.79 and 0.83 indicating good agreement between TADs from true and predicted counts (Fig. 5c). This high agreement is visually shown for a selected region (2 Mb block of 5 kb regions on chr17: 32,000,000–34,000,000) where the identified TADs (cyan boxes) agree between the true and predicted count matrices (Fig. 5d).

Overall, these validation results show that HiC-Reg predictions can be used to study three-dimensional genome organization at the level of individual loops, as well as the level of higher-order structural units such as TADs.

### HiC-Reg can predict contact counts in new cell lines

**Fig. 6:**
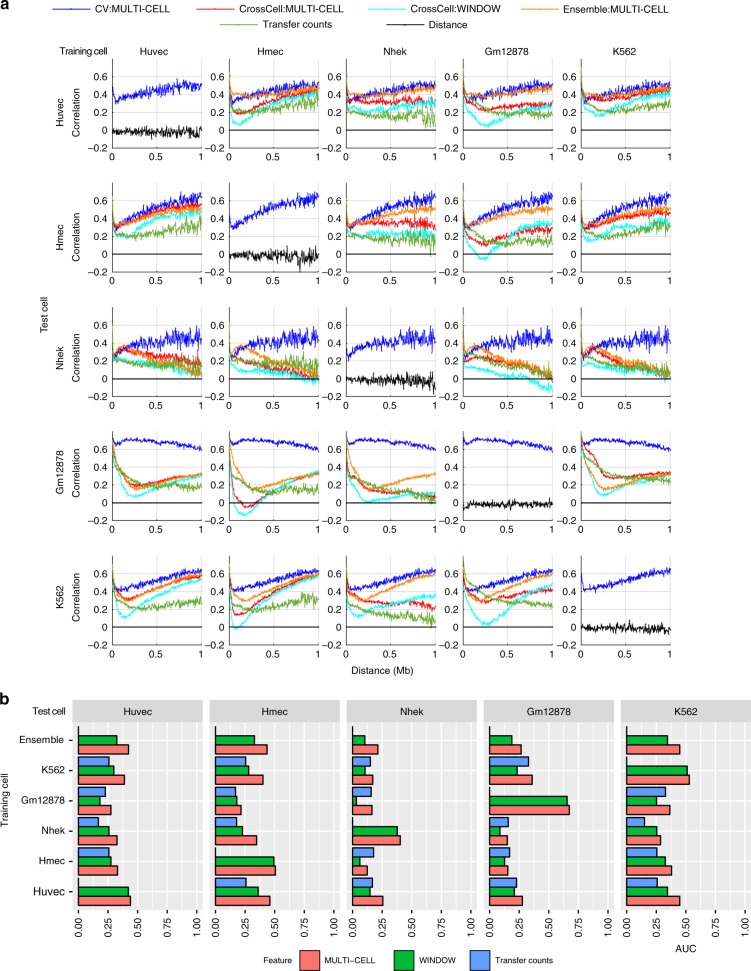
Assessing HiC-Reg predicted counts in new test cell lines. **a** Distance-stratified Pearson’s correlation plot using models trained on one cell line (columns) and tested on a different cell line (rows) for chromosome 17 considering all five cell lines: Huvec, Hmec, Nhek, Gm12878, K562. Each plot (except the ones on the diagonal) shows the distance-stratified correlation curves when training using the same cell line (blue), a different cell line using MULTI-CELL (red) and WINDOW (cyan) features, using the ensemble of all predictions from a different cell line (orange), and when simply transferring counts (green). The plots on the diagonal show the CV performance. **b** Area under the curve (AUC) for distance-stratified correlation in chromosome 17. Shown are the AUCs for the different training cell line models for both feature types (red and green), ensembles from both feature types, as well as transfer count when predicting in a new cell line. Source data are provided as a Source Data file.

**Fig. 7:**
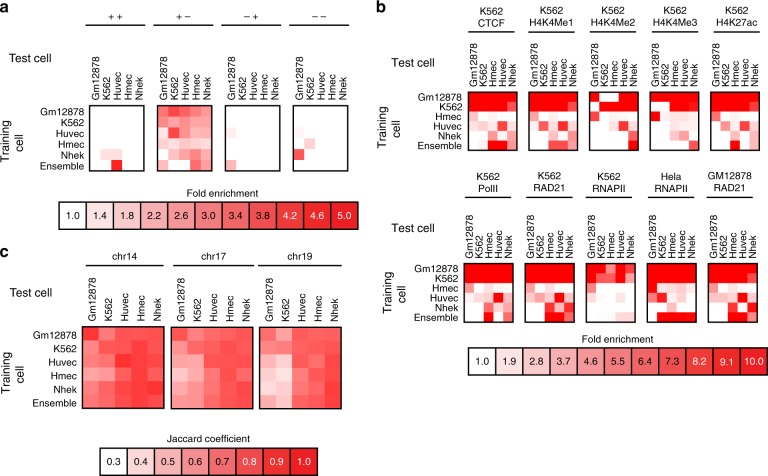
Ability of HiC-Reg to capture significant interactions and TADs in new cell lines. **a** Enrichment of CTCF motifs in four configurations for cross-cell predictions. Shown are the fold enrichments when testing using a model trained on a different cell line or an ensemble of models using MULTI-CELL features. The diagonal entries correspond to the fold enrichment obtained in the cross-validation setting. **b** Fold enrichment of ChIA-PET interactions in predicted interactions when training on a different cell line, as well as when using the MULTI-CELL ensemble. **c** Jaccard coefficient-based similarity of TADs called on true and predicted interaction counts when training on a different cell line. Shown are Jaccard similarity values for three chromosomes, 14, 17, and 19. Source data are provided as a Source Data file.

Our analysis so far demonstrates the feasibility of using regression to predict contact counts in cell lines with available Hi-C data for at least some chromosomes. We next asked if we could apply this approach to predict interactions in test cell lines different from the training cell line. This would enable us to study the utility of HiC-Reg in cell lines where Hi-C data are not yet available. We applied HiC-Reg trained on one cell line to predict counts for pairs from a different test cell line. We evaluated the quality of the predictions in the test cell line using the distance-stratified Pearson’s correlation (CrossCell, Fig. 6), the Area under the distance-stratified Pearson’s correlation curve (AUC) and additional validation metrics (CTCF directionality, ChIA-PET and TAD recovery, Fig. 7). These validation metrics were computed on the test cell line and compared against different models: (i) model trained on distance alone, (ii) model trained with cross-validation (CV) on the test cell line (CV, Fig. 6), and (iii) a new baseline model which simply transferred the count from the training cell line to the test cell line (TransferCount, Fig. 6).

A model trained on a cell line different from the test cell line is significantly better than a model trained on distance alone, but is often worse than a model trained on the same cell line (Fig. 6). For example, for chr17, the same cell line CV model has the best performance (blue line Fig. 6a) compared to all versions of cross cell line predictions. Here too we observe that the MULTI-CELL features (Fig. 6a, red line) are better or at least as good as the WINDOW features (Fig. 6a, cyan line). Compared to transferring counts (TransferCount, green line Fig. 6a), both MULTI-CELL and WINDOW have significant benefits at long distance relationships (green line is usually below the red and cyan lines after $\sim$250 kb). The AUC offers a concise summary of this behavior (Fig. 6b), with models using MULTI-CELL features being at least as good as TransferCount models in the majority of training-test cell line combinations. Overall, the Gm12878 cell line was the hardest to predict using a model from other cell lines.

When comparing the predictions in other chromosomes (Supplementary Figs. 19 and 20), we observe a similar behavior. Interestingly, the extent to which a cell line can be predicted from a model in a different cell line depends greatly on the test cell line. In particular, on Gm12878, which has the highest sequencing depth for Hi-C data, none of the models were able to come up to par with the model trained and tested on Gm12878 (Fig. 6a, fourth row, Fig. 6b, fourth column). In contrast, for Huvec, the K562 model is able to predict interactions nearly as well as the CV model especially when using the MULTI-CELL features. Similarly, for Hmec, Huvec-trained model was able to recapitulate the performance of the Hmec CV-trained model to a great extent (Fig. 6b). Previously, we have shown that an ensemble model of combining predictions from multiple models provided a robust performance in a new cell line^14^. Therefore, we combined the predictions from the models trained on each of the cell lines by taking the average of the predictions (Fig. 6, ENSEMBLE). We find that the ensemble predictions are at least as good as the predictions from the individual models (Fig. 6a orange line) and the ensemble for the MULTI-CELL features is better than that for the WINDOW features (Fig. 6b).

As additional validation we tested our predictions for enrichment of ChIA-PET interactions, CTCF bidirectional loops and TAD recovery (Fig. 7). We again applied Fit-Hi-C to the predicted counts and found that the predicted interactions in each of the cell lines tested were significantly enriched for bidirectional motifs (Fig. 7a), and were significantly enriched for ChIA-PET interactions (Fig. 7b). Furthermore, there was good agreement between TADs identified from true and predicted counts in each of these chromosomes based on the Jaccard coefficient score (Fig. 7c).

We also compared the cross-cell line predictive performance of a HiC-Reg model trained with the reduced set of datasets selected by the MTG-RF approach relative to a model trained on all datasets in a cross-cell line setting (Supplementary Fig. 21). The HiC-Reg model trained on the top eight datasets is better than the model using the top six datasets (Supplementary Fig. 21) and at par with the model trained on the full 14 datasets. Thus, for a new cell line, CTCF, DNase I, H3k9me3, H4k20me1, RAD21, TBP, would be useful to obtain competitive but slightly diminished performance, whereas additionally including H3k4me1 and H3k79me2 can provide at par performance as the full set of 14 datasets.

Overall, these results suggest that HiC-Reg can be used to predict interactions in new cell lines and it performs better than baseline approaches based on distance alone or simply transferring counts. There is a dependence on the training cell line and an ensemble approach offers a robust way to combine predictions from multiple training models.

### HiC-Reg recovers high-confidence manually curated loops

**Fig. 8:**
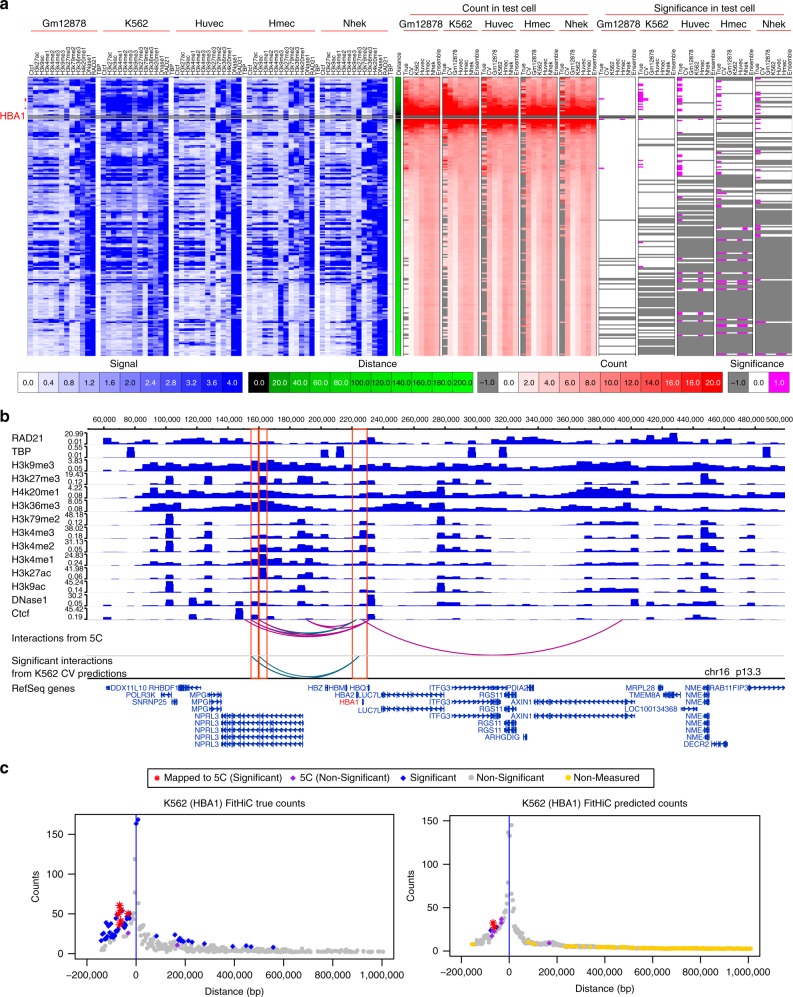
Examining HiC-Reg predictions at the *HBA1* locus. **a** Shown are the features, true and predicted counts and *q*-value for $\sim$225 kb before and 1 MB after the 5 kb bins spanning the *HBA1* gene promoter. Note, no features were measured for bins within 0 to 55 kb. The white-red heatmaps show the true counts for five cell lines and predicted counts, and the white-magenta heatmaps show *q*-value significance (0: *q*-value $\ge$ 0.05 or 1: *q*-value $<$ 0.05, *q*-values are assigned only to pairs that have a measured count in the original Hi-C data). Predicted counts are from five CV models, 20 cross-cell line models, and five Ensemble models. Test cell lines are mentioned above the red line, while the column names (vertical orientation) are for the training models. The white-blue heatmaps show the ChIP-seq and motif feature signals while the green column is for distance (colorscale: 0: 0 kb and 200:1 MB). The asterisks denote the 5 kb regions that interact with the 5 kb region spanning the *HBA1* gene promoter from 5C. Only the bin with significant predicted interactions is shown. **b** Visualization of feature signals using WashU Epigenome Browser for significant interactions obtained from a model trained in K562 and tested in K562. Shown are also overlapping interactions with 5C pairs (magenta and green). Red vertical lines demarcate the 5 kb bin overlapping the *HBA1* gene promoter and the distal region that interacts with it, as supported by 5C. The green arcs on both tracks depict the interactions predicted by HiC-Reg and supported by 5C. **c** Manhattan style plots of true and CV predicted counts around 1 MB radius of *HBA1* promoter in K562. Interactions associated with both bins spanning *HBA1* promoter are shown. The left part of each Manhattan plot is shortened because this region is towards the beginning of the chromosome. Blue diamonds are significant interactions, red stars are significant interactions that overlap a 5C interaction and purple diamonds are pairs overlapping a 5C interaction but not significant. Yellow dots are predictions for pairs that are not measured in K562. Gray dots denote pairs that are measured but are not significant. Source data for **a** and **c** are provided as a Source Data file.

To gain deeper insight into the features that drive interactions between two specific loci, we next focused on examples of well-characterized long-range interactions. Distal regulation of the *HBA1* gene by a regulatory element 33–48 kb away^27^ has been experimentally characterized using low-throughput^27^ and high-throughput methods such as 5C^28^. We focused on significant interactions associated with the 5 kb bins containing the *HBA1* promoter and the 225 kb before and 1 MB after these bins (*HBA1* gene is located towards the beginning of chr16 and the full 1 MB radius is not available). We first applied Fit-Hi-C on the true counts from each of the cell lines and found that K562 had among the largest number of significant interactions (Fig. 8c and Supplementary Table 7). This is consistent with this gene being specific to erythroid cells^27^. Next, we considered the predicted interactions using the CV, as well as cross-cell line models. Across all models, we found between 1 and 14 significantly interacting pairs associated with the *HBA1* promoter (minimum number of significant interactions is 1 and maximum is 14), with 2 significantly interacting pairs from the K562 CV model (Supplementary Table 7). Both of the significantly interacting pairs from the K562 CV model overlapped with 5C detected interactions (Fig. 8b, green arcs, Fig. 8c) at 27 kb and 32 kb, similar to the true counts. Similar number of significant interactions are called when using a different interaction caller by Duan et al.^29^, which is based on a binomial model, suggesting the identification of these interactions is not specific to Fit-Hi-C (Supplementary Fig. 22B). Visualization of the regulatory signals spanning the *HBA1* gene and its interacting regions in the WashU genome browser^30^, showed chromatin marks, including H3K9me3, H4K20me1, H3K36me3, H3K4me1, H3K4me2, H3K27ac as important features and CTCF and DNase I as additional contributors to these interactions (Fig. 8b). Examination of features based on their usage count in the significant interactions showed that the top features came primarily from the Window region of K562, Nhek, or Hmec (Supplementary Fig. 23A–C), and included features such as CTCF, DNase I, TBP, and chromatin marks, such as H3K4me1 and H3K27ac.

We next investigated the *PAPPA* gene locus, which is implicated in the development of mammary glands and is of interest in breast cancer studies^31,32^ and identified significant interactions in the 1 MB radius around the *PAPPA* gene using true and predicted counts. The rat ortholog of *PAPPA* is regulated by a 8.5 kb genomic region called the temporal control element (TCE) in rat mammary epithelial cells^32^. The TCE resides within the MCS5C genomic locus associated with breast cancer susceptibility and is conserved in human and mouse^32^. When analyzing true counts, we found the largest number of significant interactions in the Hmec cell line (Supplementary Fig. 22C). Of these, 16 interactions overlapped the MCS5C region and 3 overlapped the TCE region. The Hmec cell line is a primary mammary epithelial cell line, which indicates that these interactions are relevant to the breast tissue. A number of significant interactions were identified also in the true counts from Huvec and Nhek (Supplementary Table 8), suggesting some extent of shared interactions across cell lines. Based on the predicted counts using the CV and cross-cell line models, we found a greater number of significant interactions in models associated with Hmec, which is consistent with the behavior on the true counts (Supplementary Fig. 24 and Supplementary Table 8). We found two significant interactions that connected *PAPPA* to regions in the MCS5C locus when using the Hmec CV predictions (Supplementary Fig. 24C, left), and between 1 and 37 interactions in cross-cell line predictions when using models trained on the Hmec cell line (Supplementary Table 8). The CV predictions in Hmec and cross cell predictions in Huvec using models from Hmec also captured several of the finer TCE-*PAPPA* interactions (Supplementary Fig. 24C). Visualization of the signals (Supplementary Fig. 24B) and feature analysis on the significant interactions indicated that CTCF, TBP, and H3K4me1 measured in the Hmec cell line are important for this interaction (Supplementary Fig. 23D–F). In summary, HiC-Reg predictions provide computational support for the long-range regulation of the *PAPPA* gene in a relevant human cell line, which was originally studied in the rat mammary cells.

Beyond these two well-characterized examples, we examined other examples curated in the literature that were shown to have a distal enhancer regulating a gene^33^. Several enhancer-gene interactions have been studied in mouse embryonic stem (ES) cells^33^, which showed that CRISPR deletion of the implicated enhancer decreased the expression of the target gene. We examined the predicted counts in the 1 MB radius of ten such enhancers that could be mapped to the human genome (hg19) and identified significant interactions that overlapped genes. We found significant interactions around three genes *MACF1*, *MCL1*, and *KIAA1217* in the Hmec cell line (Supplementary Fig. 25). Moorthy et al.^33^ reported relatively lower decrease in expression of these genes (18–40%) compared to other genes (e.g., *Sall1*, *Tet1*), which suggests that these interactions may not be specific to embryonic stem cells (ESC) and could be detected in other contexts. We did not detect the interactions that reported 80–90% decrease in expression, likely because they are specific to ESCs.

Taken together, our fine-grained analysis of these curated loci known to be involved in long-range regulatory interactions provide further support of our predictions, highlight potentially important features that facilitate these interactions and serve as case studies of how HiC-Reg could be used to characterize a particular locus of interest. In many of these cases we found additional loci that are predicted to interact with these genes, which can be followed with future experiments.

## Discussion

The three-dimensional organization of the genome can affect the transcriptional status of a single gene locus, as well as larger chromosomal domains, both of which can have significant downstream consequences on complex phenotypes. Although high-throughput chromosome capture conformation assays are rapidly evolving, measuring cell line-specific interactions on a genome-wide scale and at high resolution is a significant challenge. In this work, we described a novel computational approach, HiC-Reg that can predict the contact count of two genomic regions from their one-dimensional regulatory signals, which are available for a large number of cell lines and experimentally more tractable to generate than Hi-C datasets. As HiC-Reg directly predicts counts, instead of classifying interactions from non-interactions as has been commonly done^14,15,18^, the output from HiC-Reg can be used to identify significant interactions using peak-calling algorithms (e.g., Fit-Hi-C^21^), as well as examine more large-scale organizational properties using domain finding algorithms (e.g., DomainCaller^26^, TopDom^34^, HiCSeg^35^).

A key challenge we addressed using HiC-Reg was to generate high-resolution interaction counts in a new chromosome or cell line of interest. The former is relevant to predict interactions among regions that might not have been experimentally assayed. The latter is useful for cell types and developmental stages that may not be amenable to large-scale high-throughput 3C experiments and computational predictions could prioritize regions for targeted experimental studies. Our cross-chromosome experiments showed that the performance decreases when training on one chromosome and testing on another. The features identified as important across different chromosomes are very similar (Supplementary Figs. 7 and 8), suggesting that the overall properties governing chromosomal contact are similar across chromosomes, however, there may be fine-grained differences that are not being captured by the Random Forests regression model. Incorporation of additional measurements from transcription factor binding could be beneficial for capturing these differences. Our cross-cell line prediction shows that the performance can vary from one training cell line to another, making the choice of the training cell line non-trivial. Our ensemble approach that aggregated predictions from multiple predictive models was better or comparable to the best performance from a model trained on any one cell line, however, more systematic approaches to combine shared information across different cell lines such as considering different weighting strategies will be an important direction of future work.

A second issue we considered was determining the most important genomic datasets for predicting contact counts. Our predictive framework recapitulated known players of long-range gene regulation such as CTCF and cohesin^36^, together with additional components of the transcription machinery such as chromatin marks and general transcription factors, several of which have not been thoroughly characterized in the context of long-range interactions. We examined the importance of these features globally for all pairs, for sets of pairs, and for individual pairs. Our analysis showed that in addition to CTCF, elongation and repressive marks can also be important for predicting counts. The importance of elongation marks such as H3K36me3 in predicting contact count is consistent with existing work^9,37^, which showed that H3K36me3 and elongation-related signals are enriched in regions participating in long-range interactions^9^, and higher-order genome organization^37^. The identification of repressive marks such as H3K27me3 could be explained by their association in large-scale transcriptional units such as compartments^11^, TADs^12^ and intra-TAD loops^38^, and the specific pairs with these repressive marks could be specifically transcriptionally silenced or be in a poised state^11^. Finally, we determined that a total of eight datasets, including cohesin, histone marks and accessibility should be sufficient to predict interaction counts of comparable performance as the full set of 14 datasets. These results should be informative for feasibly generating models and predictions in new cell lines and cell types.

A third important issue in the count prediction problem is feature representation of a pair of genomic loci. We studied different ways of modeling pairwise information and found that incorporating the signal between the interacting regions (WINDOW) is important for generalization to new chromosomes and cell lines. Our results are consistent with the finding of Whalen et al.^15^, who showed that the WINDOW features were informative in the classification setting. Furthermore, integrating regulatory genomic datasets from other cell lines as features (MULTI-CELL) can further improve performance in a cross-chromosome or cell line setting, likely because it can capture additional variation in the Hi-C interaction profile in new cell lines. However, the MULTI-CELL features might be difficult to generate due to unavailability of data in other cell lines and might be more computationally intensive to train models. The WINDOW features provide a good balance between performance and computational resources needed to train a model.

We evaluated the predictions from HiC-Reg using different validation metrics, globally using measurements from complementary assays, as well as, at specific loci that have been studied in the literature through high-quality, albeit low-throughput experiments. We demonstrated the utility of HiC-Reg in studying the long-range regulatory landscape of two loci, including the well-studied *HBA1* locus, as well as a relatively less studied locus, *PAPPA*. Our analysis of the *PAPPA* locus provided support of long-range regulation of *PAPPA*, originally identified in rat, in a relevant human cell line. Compared to a classification-based approach, our HiC-Reg predictions have greater sensitivity and are able to rank interactions better (Supplementary Fig. 26).

HiC-Reg exploits the widely available chromatin mark signals that are experimentally easier to measure compared to the Hi-C experiment. HiC-Reg however relies on the availability of these marks in new contexts, which may not always be available. Several groups have started to explore imputation strategies of chromatin marks^39,40^. An important direction of future work would be to examine how HiC-Reg performs with imputed marks as this would greatly increase the impact of a predictive modeling framework such as HiC-Reg. Another direction of future work would be to examine high-throughput contact counts from other types of Hi-C experiments, for example, Capture-Hi-C^9^, and test if integrating data from multiple platforms can improve the performance of the regression model.

In summary, we have developed a regression-based framework to predict interactions between pairs of regions across multiple cell lines by integrating published Hi-C datasets with one-dimensional regulatory genomic datasets. As additional chromatin mark signals and Hi-C data become available, our method can take advantage of these datasets to learn better predictive models. This can be helpful to systematically link genes to enhancers, as well as to interpret regulatory variants across diverse cell types and diseases.

## Methods

### Random Forests regression model in HiC-Reg

HiC-Reg is based on a regression model to predict contact counts measured in a Hi-C experiment using features derived from various regulatory genomic datasets (e.g., ChIP-seq datasets for histone modifications, transcription factor occupancies, Fig. 1). HiC-Reg uses Random Forests as its main predictive algorithm. Random Forests are a powerful tree ensemble learning approach that have been shown to have very good generalization performance^41^, and have been applied to a variety of predictive problems in gene regulation^42–44^. The Random Forests model is learned using the bagging algorithm with random feature selection from Brieman et al.^41^. Each tree in our Random Forests is a regression tree, trained on a bootstrap sample of the training set. To learn the tree, we start with all examples in the bootstrap sample at the root node. Next, for each leaf node that can be split, we randomly selected one third of the features and searched for the best split for each feature. We split a node into two children nodes based on the feature and a threshold value for the feature. The quality of the split is based on the difference in prediction error of training examples before and after the split. We trained Random Forests on the different feature encodings and Hi-C SQRTVC normalized contact counts downloaded from Rao et al.^20^. However, HiC-Reg can be used on datasets generated using other normalization schemes (see Section Testing HiC-Reg at multiple normalizations and resolutions). We experimented with different number of trees (Supplementary Fig. 1D) and found that beyond 20 trees there was no significant improvement in performance. Hence we performed all subsequent experiments with 20 trees. We compared the Random Forests regression model to a linear regression model using the different feature encodings of a pair, namely, WINDOW, PAIR-CONCAT, MULTI-CELL (See Feature extraction and representation). We found that the non-linear regression model based on Random Forests performs significantly better than a linear regression approach (Supplementary Fig. 1A–C). We next describe the training and test generation and different feature representations of a pair of regions.

### Generation of training and test sets

To generate training and test datasets for our regression models, we first binned each chromosome into 5 kb non-overlapping regions. We randomized the regions and split them into five sets. Each time, we select one of the five sets as the test set of regions and the remaining four as training, repeating this for all five sets of regions. Within each training or test set of regions we generate all pairs of interactions that are within a 1 MB radius. As the Hi-C matrix is symmetric, we need to only predict the upper triangle of the matrix and hence our pairs are not redundant. In each pair, the region with the smaller coordinate is referred to as the R1 region and the region with the larger coordinate as the R2 region. We conducted three different types experiments to evaluate the performance of HiC-Reg: (i) same cell line, same chromosome cross-validation (CV), (ii) same cell line cross chromosome comparison, (iii) different cell line same chromosome comparison.

For the same cell line same chromosome setting, HiC-Reg was trained and tested using fivefold cross-validation using training and test pairs generated as described above. In each fold, we trained Random Forests regression models on four folds, and predict contact counts for the left-out fold. We concatenated predictions from five folds and assessed performance using distance-stratified Pearson’s correlation of true and predicted counts. As each training/test example is a pair of regions, we need to consider two types of examples: those that share a region with the training data (easy examples) and those that do not share a region with the training data (hard examples). Our same cell line same chromosome cross-validation results are generated using hard pairs only. The cross-validation experiments were done in all autosomal chromosomes.

For the same cell line cross chromosome setting, we used the five Random Forests regression models trained on each fold from the training chromosome to predict contact counts for all pairs in a test chromosome. Each pair in the test chromosome had five predictions and we took the average of these predictions as the final predicted count. We note that in this setting, all test pairs are hard. Cross-chromosome experiments were done on five chromosomes, 9, 11, 14, 17, and 19.

For different cell line same chromosome setting, we again used the Random Forests regression models trained on the training data from each fold in one cell line and generated predictions for all pairs in the test cell line. Next, we took the average of these predictions as the final predicted count. When using MULTI-CELL features, we excluded the features derived from the regulatory signals measured in the test cell line. In this setting as well, all test pairs are hard pairs. Our ensemble approach took a further average of the predictions from all four training cell lines as the predictions in a test cell line. We made cross-cell line predictions in five chromosomes: 9, 16, 14, 17, and 19. Cross-cell predictions from chromosomes 14, 17, and 19 were examined using enrichment for ChIA-PET interactions and TAD recovery. Cross-cell line predictions in chromosomes 9 and 16 were used for analyzing the interaction profile for the *PAPPA* and *HBA1* loci.

### Feature extraction and representation

To extract features for HiC-Reg’s regression framework, we used datasets from the ENCODE project for five cell lines: K562, Gm12878, Huvec, Nhek, and Hmec^45^, learning a separate model for each cell line. We selected 14 datasets that were measured in all five cell lines. These 14 datasets included ChIP-seq datasets for ten histone marks and CTCF, DNase I-seq and DNase I-seq-derived motifs of RAD21 and TBP, which we had previously found to be helpful for predicting enhancer-promoter interactions in a classification setting^14^. A ChIP-seq signal is represented as the average read count aggregated into a 5 kb non-overlapping bin. We obtained the raw fastq files from the ENCODE consortium^45^, aligned reads to the human hg19 assembly using bowtie2^46^, retrieved reads aligned to a locus using SAMtools^47^ and applied BEDTools^48^ to obtain a base pair level read count. We next aggregated the read counts of each base pair in a 5 kb region. Next, we normalized aggregated signal by sequencing depth and collapsed replicates by taking the median. As TBP and RAD21 ChIP-seq data are not available in Huvec, Nhek  and Hmec cell lines, we predicted the binding sites using PIQ^49^ on the DNase I data and used the sum of purity scores for all motifs mapped to the same 5 kb bin as the signal value. We performed a simple depth normalization on the counts to enable comparison across cell lines. The depth normalization does not affect the overall performance based on the Area under Pearson’s correlation curve, but could affect evaluation metrics such as distance-stratified mean-square error if they were used to compare different predictions. This could be particularly an issue for cross-cell line predictions that simply transfers counts (one of our baseline models).

We represented features of a region as a 14-dimensional feature vector, each dimension corresponding to one of the 14 genome-wide datasets (Fig. 1). To generate a feature vector for a pair of regions, we used different strategies: PAIR-CONCAT, WINDOW, and MULTI-CELL. In the PAIR-CONCAT case, we concatenated the 14-dimensional feature vectors of the two regions to obtain a feature vector of size 28. In the WINDOW case, we concatenated the 14-dimensional feature vectors of the two regions together with the feature vectors of the intervening region between the two regions to obtain a feature vector of size 42. We call this the WINDOW feature following Whalen et al.^15^. The feature with the intervening region is a mean signal value of the feature in the region. In the MULTI-CELL case, we concatenated the 42-dimensional feature vectors of the two regions from all five cell lines to obtain a merged feature vector of size 210. Finally, for all these feature representations, we included genomic distance between the two regions of a pair as an additional feature.

We benchmarked HiC-Reg on different datasets from different cell lines. The runtime and memory usage of HiC-Reg greatly depends upon the number of features and the depth of the data (Supplementary Data 1). For most practical applications of HiC-Reg, we believe the WINDOW features are most relevant. On average, HiC-Reg needs between 2 and 6 GB of memory and 2–6 min to train a tree with $\sim$2–4 million pairs using the WINDOW features. The memory and run time are higher for MUTLI-CELL features, which is expected as the number of features are roughly five times more.

### Identification of a minimal dataset for training HiC-Reg

We applied a two-step approach to identify the fewest number of datasets needed to train HiC-Reg. Our first step used Multi-task Group-LASSO (MTG-LASSO) on all five cell lines simultaneously to select features predictive of contact counts in all five cell lines. Our second step used a greedy approach, based on Random Forests (RF) to iteratively refine the feature set selected by MTG-LASSO.

The MTG-LASSO approach is a regularized regression approach that is applicable to a problem with multiple predictive tasks. In our problem setting, the different tasks are different cell lines. The objective function for MTG-LASSO is defined as:

1 $$\min_{{\bf{W}}} \frac{1}{2}\sum _{c=1}^{K}| | {{\bf{X}}}_{c}{\bf{W}}(:,c)-{{\bf{Y}}}_{c}| {| }_{2}^{2}+\lambda | | {\bf{W}}| {| }_{{l}_{1}/{l}_{2}}$$

Here, the first term is the sum of the least squares loss for cell line $c$ added across all cell lines. ${\bf{W}}(:,c)$ is the column of regression weights for the ${c}^{\mathrm{th}}$ cell line and ${\bf{W}}$ is the $n \, \times K$ matrix of regression weights across $K$ cell lines. The second term is the Group LASSO norm penalty, defined as $| | {\bf{W}}| {| }_{{l}_{1}/{l}_{2}}={\sum }_{f}| | {\bf{W}}(f,:)| {| }_{2}$, where $f$ indexes different features. This enables the selection of a small number of groups (according to the L1 norm) and encourages smoothness among the weights within each group (according to the L2 norm). In our setting each group is one feature corresponding to the row of ${\bf{W}}$. The parameter $\lambda$ controls the tradeoff between the loss and the regularization term. MTG-LASSO selects or deselects an entire group (rows), and hence selects the same feature for all cell lines. We used the implementation of this regression framework in the Sparse Learning with Efficient Projections package for MATLAB (SLEP v4.1, https://github.com/jiayuzhou/SLEP).

To perform feature selection with MTG-LASSO, we used the WINDOW feature set for each of the five cell lines for chromosome 17. We considered a range of regularization parameters from $\lambda \,$= 0.01 to $\lambda \,$= 1 and conducted fivefolds cross-validation for each value of $\lambda$. Beyond $\lambda =0.2$ only Distance is selected as feature, hence we set the upper limit to be $\lambda =0.2$. We computed the distance-stratified Pearson’s correlation curve for the test set at each $\lambda$ and the area under the curve (AUC) to assess the overall predictive performance. The model at each $\lambda$ included features that were identified in all folds. Based on the AUC, we set $\lambda =0.04$, which resulted in seven datasets (CTCF, Distance, Dnase, H3k9me3, H4k20me1, RAD21, TBP) and the AUC did not improve substantially at a lower $\lambda$ (more features). We started with this feature set as the initial set that we next refined using our greedy feature refinement approach.

The greedy feature refinement uses Random Forests as the regression model and works as follows. Starting with an initial set of features and one of the folds for training and testing, we train a Random Forests model on the training data and generate predictions for the test dataset. For each subsequent iteration, we randomly pick between adding or removing a feature, retrain the Random Forests model on the training data considering every candidate feature for addition (or removal) and select the best feature based on the change in prediction error on the test set. We add (or remove) the feature if there is gain in test error performance and do nothing if there is no feature with an improvement in test error. We repeat this procedure until a max number of iterations have been executed or the error does not change substantially. After convergence, we tabulate the features selected. We repeat this entire procedure for all five folds. As this process is compute intensive, we subsample 10% of the training data so that the pairs retain their distance distribution and select a feature set. We repeat the training data subsampling ten times for each fold resulting in a total of 50 feature sets. Finally, we rank a feature based on the fraction of times (out of 50) it is selected (Supplementary Fig. 17A). We repeat this for all five cell lines, rank the features based on their average selection frequency and select the top six or top eight datasets. The cross-validation performance for the full feature set and the top features are shown in Supplementary Fig. 17B. However, because the test set is used to do feature selection, these performance numbers are likely over-estimates. Hence we further assess the performance of the different feature sets in cross-chromosome (Supplementary Fig. 18) and cross-cell line settings (Supplementary Fig. 21).

### Testing HiC-Reg at multiple normalizations and resolutions

To examine the impact of different normalization methods on learning regression models for Hi-C data, we trained HiC-Reg models using input counts from different normalization methods: Knight-Ruiz matrix balancing (KR), Iterative Correction and Eigen vector decomposition (ICE), and Square Root Vanilla Coverage (SQRTVC). The normalized counts using the KR and SQRTVC methods are downloaded from the Rao et al. paper^20^. The ICE normalization was performed on the raw Hi-C data using the ICE algorithm implementation in the HiC-Pro package^50^. The performance of HiC-Reg under different normalization methods is very similar for the cross-validation experiments within the same chromosome for all five cells (Supplementary Fig. 27). Next, we performed cross-chromosome experiments in two cell lines: Gm12878 and K562. The performance of HiC-Reg using counts from KR and ICE are very similar and slightly worse than using SQRTVC in Gm12878 (Supplementary Fig. 28) and slightly better for some chromosomes in K562 (Supplementary Fig. 29). Finally, we also compared performance in a cross cell line setting and see that the AUC values of KR and ICE normalization are slightly better than SQRTVC, but overall HiC-Reg performs comparably well when used with different normalization methods (Supplementary Figs. 30 and 31). Thus, the usage of different normalization methods does not impact the overall performance of our Random Forests models.

In parallel, we examined HiC-Reg model training on data generated at different resolutions: 5 kb, 10 kb, 25 kb, and 50 kb in selected cell lines of varying depth (Gm12878, Huvec and Hmec), and different chromsomes (chromosome 14 and 17). As expected, the hardest task is to learn models at the highest resolution (5 kb), and the CV performance gets better with decreasing resolution (Supplementary Fig. 32A). We also asked if a model trained on a higher resolution can be used to predict counts at a lower resolution (e.g., from 5 kb bins to 10 kb bins, Supplementary Fig. 32B, C). Predictions between two regions of size 10 kb are made by summing the predicted counts from the corresponding 2-by-2 matrix of the constituent 5 kb regions. Similarly predictions at 25 kb are from the sum of the predicted counts of a 5-by-5 matrix and predictions at 50 kb are the sum from a 10-by-10 matrix of predicted Hi-C matrices of 5 kb regions. Interestingly, the performance of aggregated counts from the 5 kb bins to lower resolution bins is better than predictions using models trained at the lower resolution. This is especially striking for the cross-chromosome performance (Supplementary Fig. 32B, C), where we trained a model on one chromosome and tested performance on a different chromosome. Hence a model trained at a high resolution can predict counts at a lower resolution, however the converse is likely not true.

### Calling significant interactions on HiC-Reg predictions

The output of HiC-Reg can be analyzed using a peak-calling method such as Fit-Hi-C^21^. Fit-Hi-C uses spline models to estimate expected contact probability at a given distance. The input to Fit-Hi-C is a raw count interaction file and an optional bias file calculated by the ICE method^51^. Fit-Hi-C estimates the statistical significance of interactions using a Binomial distribution and corrects for multiple testing using the Benjamini–Hochberg method and outputs the *p*-value and corrected *q*-value for each pair of interactions. We adopted the two-phase spline fitting procedure and used a *q*-value$\,<\,$0.05 to define significant pairs. As our predictions are based on normalized contact counts, we directly used our predicted counts as input for Fit-Hi-C without a bias file. For the CV-based predictions for a cell line, we first concatenated the predictions for measured pairs across all chromosomes and conducted Fit-Hi-C analysis on these pairs. We applied the same procedure on true counts to find significant pairs. When generating significant interactions in a cross-cell line setting, we applied Fit-Hi-C in a per chromosome manner as we did not have predictions for all chromosomes. As Fit-Hi-C is recommended to be applied with ICE normalized matrices, and our predicted interactions produce the analog of SQRTVC normalized counts, we compared Fit-Hi-C outputs to those when applied to ICE normalized true matrices. We performed these comparisons both using the significant interactions defined by a *q*-value threshold ($q \, < \, 0.05$, Supplementary Table 5), as well as based on comparing the top 1, 5, and 10% interactions (Supplementary Table 6). The significant interactions identified by Fit-Hi-C on the SQRTVC and ICE normalized matrices have a significant overlap (Supplementary Tables  5 and 6). Controlling for the number of interactions indicates a good Jaccard index score (mean 0.44 $\pm$ 0.1 (standard deviation)) across different cell lines and number of pairs considered (Supplementary Table 6).

We also verified if a different interaction caller would affect our results by implementing the binomial test-based interaction caller from Duan et al.^29^ (Supplementary Tables  1 and 2). Briefly, all pairs of intra-chromosomal regions are first stratified into different distance bins. A Binomial distribution-based *p*-value is calculated for each distance bin $i$ as follows. Let ${M}_{i}$ denote the total number of intra-chromosomal pairs measured at distance bin $i$, that is, have a non-zero count. Assuming that the probability of observing any particular interaction at any given distance is uniform, the probability of observing an interaction is ${m}_{i}=\frac{1}{{M}_{i}}$. Let ${n}_{i}$ denote the total number of observed intra-chromosomal counts at distance bin $i$. For any given pair of regions with read count $k$, the *p*-value is the probability of observing $k$ or more counts and is calculated as follows:

2 $${p}{\hbox{-}}{\mathrm{value}}\ =\sum _{j=k}^{{n_{i}}}\left(\begin{array}{c} {n}_{i}\\ j\end{array}\right){m}_{i}^{j}{(1-{m}_{i})}^{{n}_{i}-j}$$

We find significant overlap between interactions called by Fit-Hi-C and the Duan et al. approach^29^, using *q*-value-based threshold (Supplementary Table 1), as well as when comparing the top 1, 5, and 10% interactions (Supplementary Table 2).

### Recovering high-confidence manually curated interactions

To test if HiC-Reg can recover well-characterized long-range interactions, we focused on significant interactions associated with several well-studied loci: (a) the *HBA1* promoter, (2) *PAPPA* promoter, (3) enhancers involved in enhancer-gene interactions validated with CRISPR^33^. The *HBA1* promoter spans two 5 kb bins on chromosome 16, the *PAPPA* promoter spans two 5 kb bins on chromosome 9, while the enhancers span between 2 and 9 bins. We generated predictions for all pairs spanning the 1 MB radius around the bins overlapping our locus of interest. For the *HBA1*, which is situated at the beginning of chromosome 16, we had 225 kb on the left of the gene. In parallel, we applied Fit-Hi-C to the true and predicted counts for each chromosome to find significant interactions, where predicted counts were generated from CV models, as well as models trained in the cross-cell line setting. To enable comparison between true and predicted counts, we restricted Fit-Hi-C only to pairs that had measured counts and therefore have *q*-values associated with only these pairs. Next, we extracted significant interactions associated with the 5 kb bins containing the locus of interest and the 1 MB radius around the promoter. For *HBA1*, we checked if these significant interactions overlap with interactions measured from 5C experiments^28^. For *PAPPA*, we checked if the significant interactions overlap with the TCE or MCS5C genomic locus on one end, and the *PAPPA* gene on the other end. For the enhancers from Moorthy et al.^33^, we extracted significant interactions associated with the 5 kb bins containing the enhancer. Finally, we checked to see if the other end of a significant interaction overlapped the 5 kb bin containing the promoter of the target gene of interest.

### Evaluation metrics

We used different metrics for assessing the quality of our predictions for contact counts. The distance-stratified Pearson’s correlation was used to directly measure the accuracy of the predicted counts. The other metrics were used to assess the quality of results after further downstream analysis of HiC-Reg predictions and compare them to similar results obtained from actual measured data. In particular, enrichment of CTCF bidirectional motifs and ChIA-PET datasets enabled us to study the quality of significant interactions identified from HiC-Reg predictions, while the TAD similarity enabled us to study the ability of HiC-Reg predictions to capture structural units of organization.

*Distance-stratified Pearson’s correlation:* to assess the quality of predicted counts from HiC-Reg, we used Pearson’s correlation of predicted contact counts and true contact counts as a function of genomic distance. We grouped pairs of regions based on their genomic distance and calculated the Pearson’s correlation of predicted and true contact counts for pairs that fall into each distance bin. We considered all pairs upto a distance of 1 MB in distance bin intervals of 5 kb. To easily compare the performance between different methods, chromosomes and cell lines, we summarized the distance-stratified Pearson’s correlation curve into the area under the curve (AUC) using the MATLAB trapz function as trapz$({\bf{f}})$, where ${\bf{f}}$ is an $n$-dimensional vector, each entry ${\bf{f}}(i)$ specifying the Pearson’s correlation between true and expected counts for distance bin $i$. This version of trapz function uses the Trapezoidal rule with unit spacing between points to calculate the area under the curve specified by ${\bf{f}}$ as follows:

3 $${\text{AUC}}\,=\frac{1}{n-1}{\mathtt{trapz}}({\bf{f}})=\frac{1}{2(n-1)}\sum _{i=1}^{n-1}({\bf{f}}(i)+{\bf{f}}(i+1))$$

Here, $i$ indicates the distance bin and $n$ is the number of distance bins. We divide the output of trapz by the number of intervals across the distance bins, $n-1$ to get a number between –1 and 1. The higher the AUC, the better the performance.

*Enrichment of bidirectional CTCF motifs*: to obtain the coordinates and orientation of the CTCF motifs, we applied the PIQ tool^49^ on cell line-specific DNase I-seq fastq files from ENCODE (http://hgdownload.soe.ucsc.edu/goldenPath/hg19/encodeDCC/wgEncodeOpenChromDnase/). PIQ gives a score from 0.5 to 1, which is proportional to the true occurrence of the motif. We selected a threshold of 0.9 to identify high-confidence CTCF motifs. We use R1 to denote the region with the smaller starting coordinate, and R2 to denote the region with the larger starting coordinate. Following Rao et al.^20^, an interaction is labeled as having a convergent CTCF orientation if R1 contains CTCF motifs on the forward strand (+ orientation) and R2 contains CTCF motifs on the reverse strand (– orientation). We only focus on pairs with at least one CTCF motif mapped to R1 and at least one CTCF motif mapped to R2. A pair can have one of the four configurations: (i) (+ +) configuration where both R1 and R2 have the motifs in the + orientation, (ii) (+ –) configuration where R1 has CTCF motifs in + orientation and R2 has CTCF motifs in the – orientation, (iii) (– +) configuration, where R1 has motifs in the – orientation and R2 has motifs in the + orientation, (iv) (– –), where both R1 and R2 have motifs in the – orientation. We counted the total number of pairs with each of these configurations and compared this with the number of significant pairs called by Fit-Hi-C using the Hypergeometric test and fold enrichment. Briefly, assume we are testing the enrichment for the (+ –) configuration. Let the total number of possible pairs in the background be $S$. Let $k$ be the total number of significant interactions with any of the configurations of CTCF motifs, let $m$ to be the total number of interactions with the (+ –) configuration of CTCF motifs and $q$ be the number of significant interactions with the (+ –) configuration of CTCF motifs. Using Hypergeometric test, we test the probability of observing $q$ or more interactions out of $k$ interactions to have the (+ –) configuration, given that there are $m$ out of $S$ total interactions that have the (+ –) configuration. Fold enrichment is computed as $\frac{q}{k}\!/\!\frac{m}{S}$ and must be >1 to be considered as significant enrichment over background.

*Comparing HiC-Reg interactions with published ChIA-PET data*: we downloaded ten published ChIA-PET datasets: PolII in HeLa and K562, and CTCF in the K562 cell line^22^, and seven ChIA-PET datasets from Heidari et al.^23^, which included RNA PolII, CTCF, RAD21, and multiple chromatin marks in K562 and Gm12878 cell lines. Our metric for evaluating these genome-wide maps is fold enrichment, which assesses the fraction of significant interactions identified from HiC-Reg that overlapped with experimentally detected measurements, compared to the fraction of interactions expected by random chance. We mapped ChIA-PET interactions onto the pairs of regions used in HiC-Reg by requiring one region of an interaction from the ChIA-PET dataset to overlap with one region of a HiC-Reg pair (e.g., R1), and the other ChIA-PET region to map to the second region (e.g., R2). Fold enrichment is defined as $\frac{{n}_{1}/{n}_{2}}{{m}_{1}/{m}_{2}}$, where ${n}_{1}$ is the number of significant interactions from HiC-Reg that overlap with an interaction in the ChIA-PET dataset, ${n}_{2}$ is the total number of HiC-Reg significant interactions, ${m}_{1}$ is the total number of interactions in the ChIA-PET dataset that can be mapped to any of the HiC-Reg pairs, and ${m}_{2}$ is the total number of possible pairs in the universe. The observed overlap fraction of interactions is ${n}_{1}/{n}_{2}$ and the expected overlap fraction of interactions is ${m}_{1}/{m}_{2}$. A fold enrichment >1 is needed in order to be considered significant.

*Assessing TADs identified from true and predicted counts*: to identify topologically associating domains (TADs), we applied the directionality index (DI) method described in Dixon et al.^26^. The method is based on Hidden Markov Model (HMM) segmentation of the DI. The DI is a score for a genomic region to measure the bias in the directionality of interactions for that region as measured in a Hi-C dataset. It is determined by the difference in the number of reads between the region and a genomic window (e.g., 2 MB) upstream of the region and the number of reads between the region and a window downstream of the region. The window is user-defined. The DI score is segmented into three states of upstream, downstream or no bias. A TAD is then defined by a contiguous stretch of downstream biased states.

We transformed our upper triangle predicted matrix at 5 kb resolution into a symmetric interaction matrix and gave this to DI as input. We used the default parameters of the package with a window size of 2 Mb for defining the Directionality Index. For comparison, we applied the same procedure to the true count matrices to identify TADs. We compared the similarity of TADs identified from the HiC-Reg predicted counts and true counts for each chromosome using a Jaccard coefficient-based score. The Jaccard coefficient measures the overlap between two sets, $A$ and $B$ and is defined as the ratio of the size of the intersection to the size of the union, $\frac{| A\cap B| }{| A\cup B| }$. The Jaccard coefficient ranges from 0 to 1, with 1 denoting complete overlap. We matched a TAD found in the true count data to a TAD found in the predicted counts based on the highest Jaccard coefficient. The Jaccard coefficient for each match was averaged across all TADs from the true counts. We repeated the matching procedure for each TAD from the predicted counts to a TAD in the true counts and averaged the Jaccard coefficient across all TADs from the predicted counts. The overall similarity between TADs from the true and predicted counts was then the average of these two averages.

### Feature analysis for single feature

To assess the importance of individual features we used the Out of Bag (OOB) Variable Importance measure^41^, which computes importance of a feature based on the change in error on Out of Bag example pairs when the feature values are permuted. We used MATLAB’s implementation of this measure. In addition we devised another importance measure, feature usage count, which counted the number of times a particular feature was used to predict the count for an example pair when it is part of the test set. Briefly, for each test example $i$ and each tree $t$ in the ensemble, let ${n}_{i}^{t}(f)$ denote the number of times feature $f$ is used on the path from the root to the leaf in tree $t$ for example $i$. The overall importance of feature $f$ is $\sum_{t\in \mathcal{T}}\sum_{i\in \mathcal{D}}{n}_{i}^{t}(f)$, where $\mathcal{T}$ stands for the ensemble of regression trees and $\mathcal{D}$ is the dataset of examples. We computed these counts on all test example pairs, as well as examples with the top 5% lowest errors. The feature importances were very similar (Supplementary Figs. 9 and 10). A key difference between MATLAB’s OOB importance and our feature usage count importance is that the OOB is computed on left out examples from the training set, which includes the easy examples. The feature usage count is computed only on the hard pairs, which do not share a region with the training pairs.

### Feature analysis for pairwise feature

To assess the importance of pairs of features, we considered all pairs of features that occur on the tree path from root to a leaf for a test example (similar to above). This approach is similar to the Foresight method^52^, which counts the number of times a pair of features co-occur on the path from the root to the leaf. The main difference between our approach and that of Foresight is that we estimate these counts on the test examples, while Foresight estimates these on the examples in each leaf node in the tree identified during training. By using the counts on the test examples, our approach is less prone to overfitting. As the feature rankings of individual features are similar when using all pairs and pairs with the top (smallest) 5% errors, we computed these counts only for the top 5% error pairs.

### NMF for identifying feature sets associated with pair sets

We developed a novel feature analysis method to identify feature sets associated with sets of region pairs based on NMF. The input to this approach is a $n \, \times m$ matrix ${\bf{X}}$ with $n$ rows corresponding to test pairs and $m$ columns corresponding to pairs of features and each entry ${X}_{ij}$ denotes the number of times feature pair $j$ is used to make a prediction for pair $i$. NMF decomposes the matrix ${\bf{X}}$ into two lower rank non-negative matrices ${\bf{U}}$ and ${\bf{V}}$, where ${\bf{X}}={\bf{UV}}$, ${\bf{U}}$ is $n \, \times k$, ${\bf{V}}$ is $k \, \times m$, and $k$ is the rank. The ${\bf{U}}$ and ${\bf{V}}$ matrices are chosen to minimize the squared error $| | {\bf{X}}-{\bf{UV}}| {| }_{2}^{2}$ of the lower dimensional reconstruction. We use MATLAB’s non-negative matrix factorization function nnmf, with $k=5$ factors to perform this factorization, which uses the alternating least squares algorithm to estimate ${\bf{U}}$ and ${\bf{V}}$. The ${\bf{U}}$ and ${\bf{V}}$ matrices provide a low-dimensional representation of the interaction pairs and feature pairs respectively. For ease of interpretation, we normalized the ${\bf{U}}$ matrix to make each row sum to unity (denoted as $\overline{{\bf{U}}}$) and normalized the ${\bf{V}}$ matrix to make each column sum to unity (denoted as $\overline{{\bf{V}}}$). To identify sets of features associated with sets of examples, we used the rows and columns of the $\overline{{\bf{U}}}$ and $\overline{{\bf{V}}}$ matrices. Specifically, we assigned example $i$ to cluster $c$ if $c=\arg {\max }_{c^{\prime} }\overline{{\bf{U}}}(i,c^{\prime} )$. Similarly a feature pair $j$ is assigned to cluster $c$ if $c=\arg {\max }_{c^{\prime} }\overline{{\bf{V}}}(c^{\prime} ,j)$. The rows of ${\bf{U}}$ and columns of ${\bf{V}}$ have one-to-one correspondence, thus providing a natural bi-clustering output. We were able to successfully factor the matrix ${\bf{X}}$ showing there are groups of pairs clearly associated with groups of feature pairs. To further interpret these feature pairs, we visualized the pairwise interactions as networks in Cytoscape^53^, with node size proportional to the number of feature pairs they are associated with and edge weights corresponding to the strength of the association. The NMF-based feature analysis enabled us to extract groups of examples associated with sets of feature pairs.

### Reporting summary

Further information on research design is available in the Nature Research Reporting Summary linked to this article.

## Supplementary information

Supplementary Information

Reporting Summary

Description of Additional Supplementary Files

Supplementary Data 1

## Data Availability

Hi-C SQRTVC normalized contact counts were downloaded from Rao et al.^20^, Gene Expression Omnibus dataset GSE63525. ChIP-seq datasets of histone marks in five cell lines were downloaded from http://hgdownload.cse.ucsc.edu/goldenPath/hg19/encodeDCC/wgEncodeBroadHistone/. DNase I-seq datasets in five cell lines were downloaded from http://hgdownload.cse.ucsc.edu/goldenPath/hg19/encodeDCC/wgEncodeOpenChromDnase/. The source data underlying Figs. 5a–c and 7a–c are provided as a Source Data file. The source data underlying Figs. 2, 3, 6, 7, and 8a, c and Supplementary Figs. 1–10, 19, 20, 22, 24–26 are provided at 10.5281/zenodo.3525514 and additionally described in the Source Data File. Examples of trained models and predictions for different experiments performed are provided in 10.5281/zenodo.3525432 and 10.5281/zenodo.3525510.

## Source Data

Source data

## Code availability

The HiC-Reg code and associated MATLAB and R scripts to compute various validation metrics are available at https://github.com/Roy-lab/HiC-Reg.
