# Supplementary material for: In silico prediction of high-resolution Hi-C interaction matrices
Source: Nat Commun. 2019 Dec 6;10:5449. doi: 10.1038/s41467-019-13423-8 (PMC6898380; doi:10.1038/s41467-019-13423-8)
Supplement: Supplementary file 1 — Supplementary Information [file 41467_2019_13423_MOESM1_ESM.pdf]

Supplementary Information

In silico prediction of high-resolution Hi-C interaction matrices

Zhang et al.

Table of Contents

List of Figures

1 Linear versus non-linear regression 5

2 Cross-chromosome generalization performance of HiC-Reg in the Gm12878 cell line 6

3 Cross-chromosome generalization performance of HiC-Reg in the K562 cell line 7

4 Cross-chromosome generalization performance of HiC-Reg in the Huvec cell line 8

5 Cross chromosome generalization performance of HiC-Reg for the Hmec cell line 9

6 Cross chromosome generalization performance of HiC-Reg for the Nhek cell line 10

7 Feature ranking based on out-of-bag feature importance across multiple chromosomes . . . 11

8 Feature ranking based on counting feature usage across multiple chromosomes 12

9 Comparison of feature rankings using all pairs versus pairs with lowest 5% errors 13

10 Comparison of top 20 features ranked using all pairs vs pairs with lowest 5% errors 14

11 NMF analysis of pairs of features in K562 15

12 NMF analysis of pairs of features in Huvec 16

13 NMF analysis of pairs of features in Hmec 17

14 NMF analysis of pairs of features in Nhek 18

15 NMF analysis of individual feature usage in Gm12878 19

16 NMF analysis of individual feature usage in Huvec 20

17 Building a minimal model using features selected by MTG-RF 21

18 Cross-chromosome performance of MTG-RF reduced dataset vs full dataset 22

19 Generalization of HiC-Reg to new cell lines on chromosome 14 23

20 Generalization of HiC-Reg to new cell lines on chromosome 19 24

21 Cross-cell performance of MTG-RF reduced feature set vs full feature set. 25

22 Manhattan plots showing significant interactions associated with *HBA1* and *PAPPA* genes for true counts and predicted counts identified by different interaction calling methods 26

23 Feature analysis of loci participating in well-known long-range interactions 27

24 Visualization of interactions associated with the *PAPPA* gene 28

25 Manhattan plots showing significant interactions associated with other loci from the literature involved in long-range interactions 30
26 Predicting interactions associated with *HBA1* and *PAPPA* genes using classification 31
27 CV performance of HiC-Reg on data from different normalization methods 32
28 Cross-chromosome performance using Hi-C data processed using different normalization methods in the Gm12878 cell line 33
29 Cross-chromosome performance using Hi-C data processed using different normalization methods in the K562 cell line 34
30 Comparing cross-cell line performance of Knight-Ruiz normalized counts vs SQRTVC normalized counts 35
31 Comparing cross-cell performance of ICE normalized counts vs SQRTVC normalized counts 36
32 Performance of HiC-Reg at different resolutions 37

List of Tables

1 The Jaccard Index comparing the significant interactions identified from Fit-Hi-C and Duan et al on true counts 38

2 The Jaccard Index comparing the top 1%, 5% and 10% interactions identified from Fit-Hi-C and Duan et al on true counts 39

3 The overlap of Fit-Hi-C interactions selected at a cutoff of q-value < 0.05 called on true and predicted interaction counts 40

4 The overlap of top 5% interactions ranked using Fit-Hi-C q-values on the true and predicted counts 41

5 The overlap of Fit-Hi-C interactions selected at a cutoff of q-value < 0.05 between ICE and SQRTVC normalized counts 42

6 The overlap of top 1%, 5%, 10% interactions ranked using Fit-Hi-C q-value scores between ICE and SQRTVC normalized counts 43

7 Number of significant interactions associated with *HBA1* gene and how many of them overlapped with 5C dataset identified by Fit-Hi-C in different prediction settings and true counts 44

8 Number of significant interactions associated with *PAPPA* gene and how many of them overlapped with MCS5C region and TCE region identified by Fit-Hi-C in different prediction settings and true counts 45

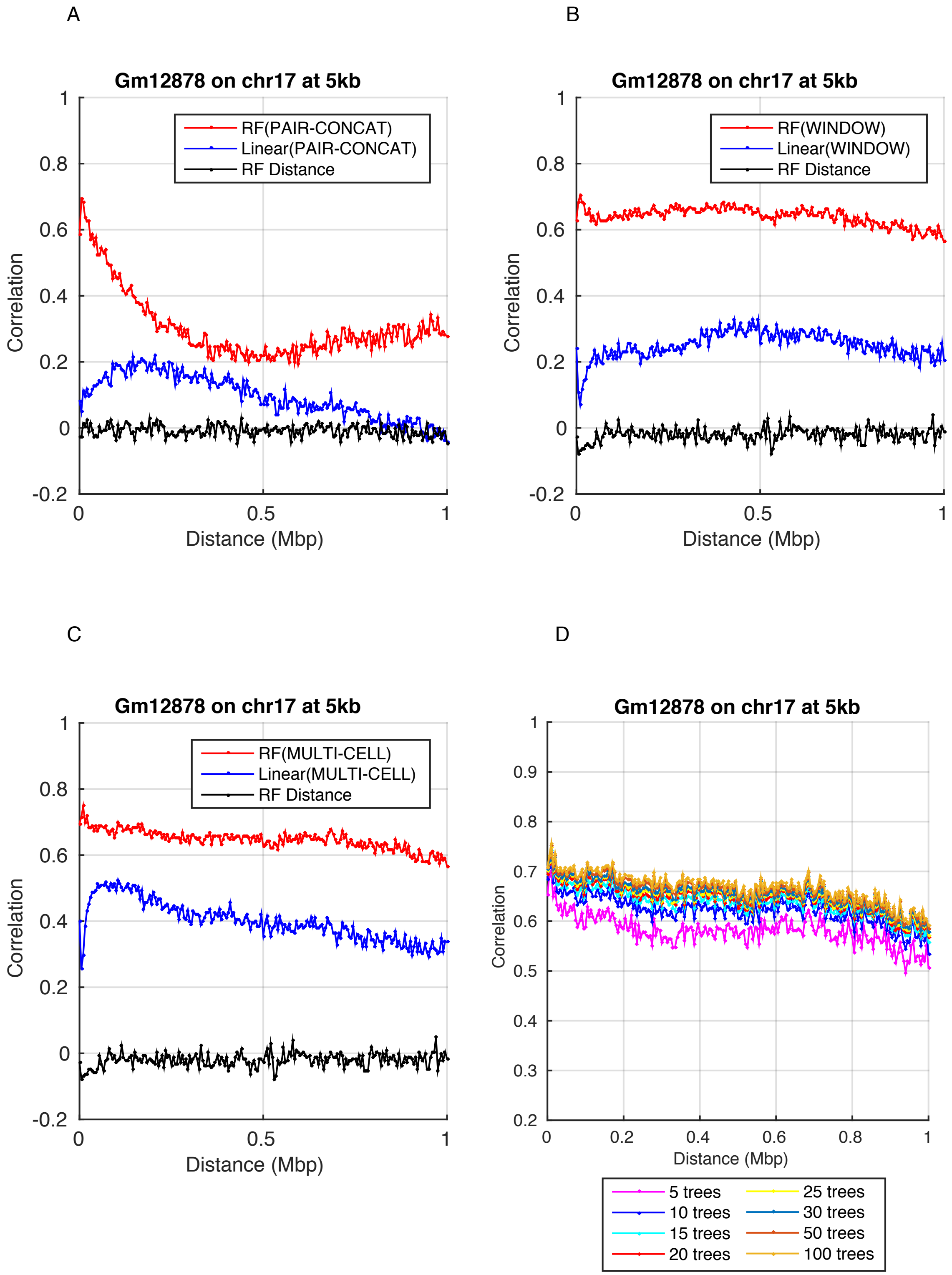

Supplementary Fig 1. Comparison of Random Forests versus linear regression models for predicting Hi-C interaction matrices using different feature encodings for a pair. **A.** PAIR-CONCAT features, **B.** WINDOW features, **C.** MULTI-CELL features. **D.** Performance of HiC-Reg with different number of trees using the MULTI-CELL features.

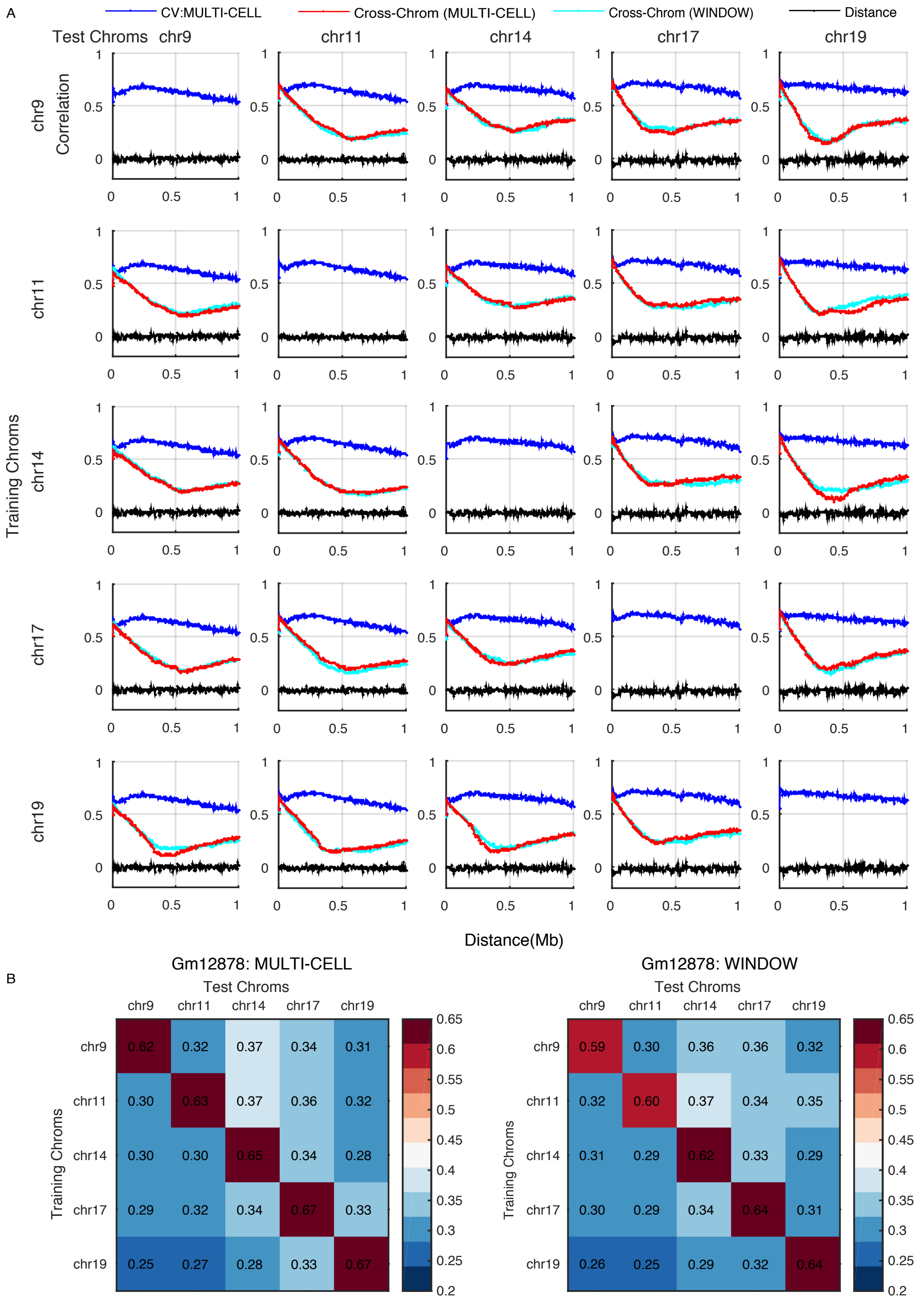

Supplementary Fig 2. Cross-chromosome performance in Gm12878. **A.** Distance stratified Pearson's correlation plot for same-cell cross-chromosome performance for models trained and tested on one of chromosome 9, 11, 14, 17, 19. The rows correspond to the training chromosome and the columns correspond to the test chromosome. Random Forests models use either the WINDOW (cyan) or the MULTI-CELL (red) feature. For comparison, the cross-validation plots (blue lines) are shown in all plots and is the same for a column. **B.** Heatmap of AUC values for cross-chromosome performance in Gm12878. The diagonal entries in the heatmap correspond to the AUC value when HiC-Reg is trained and tested on the same chromosome in CV mode.

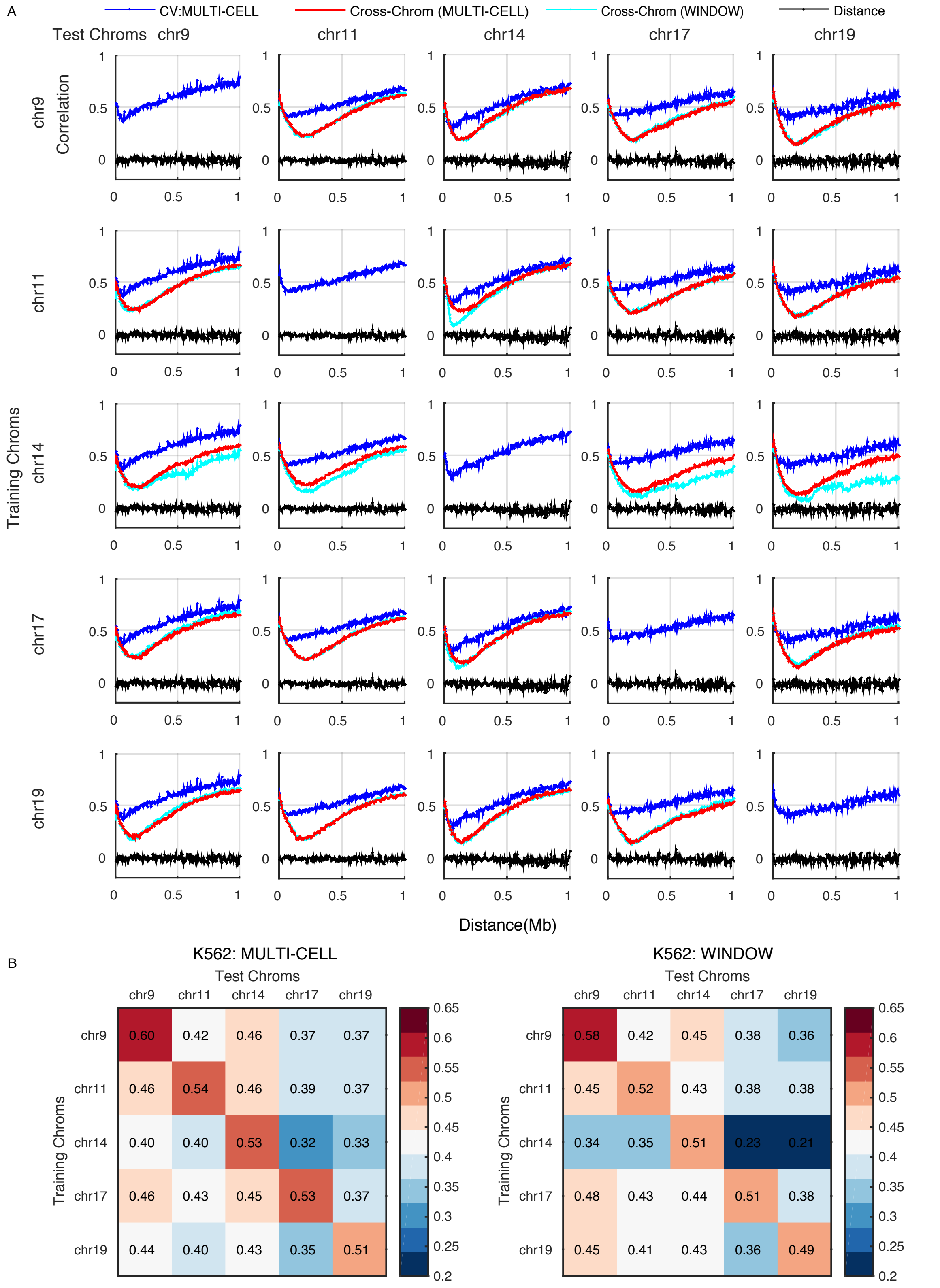

Supplementary Fig 3. Cross-chromosome performance in K562. The figure panels follow the same legend as **Supplementary Fig 2**.

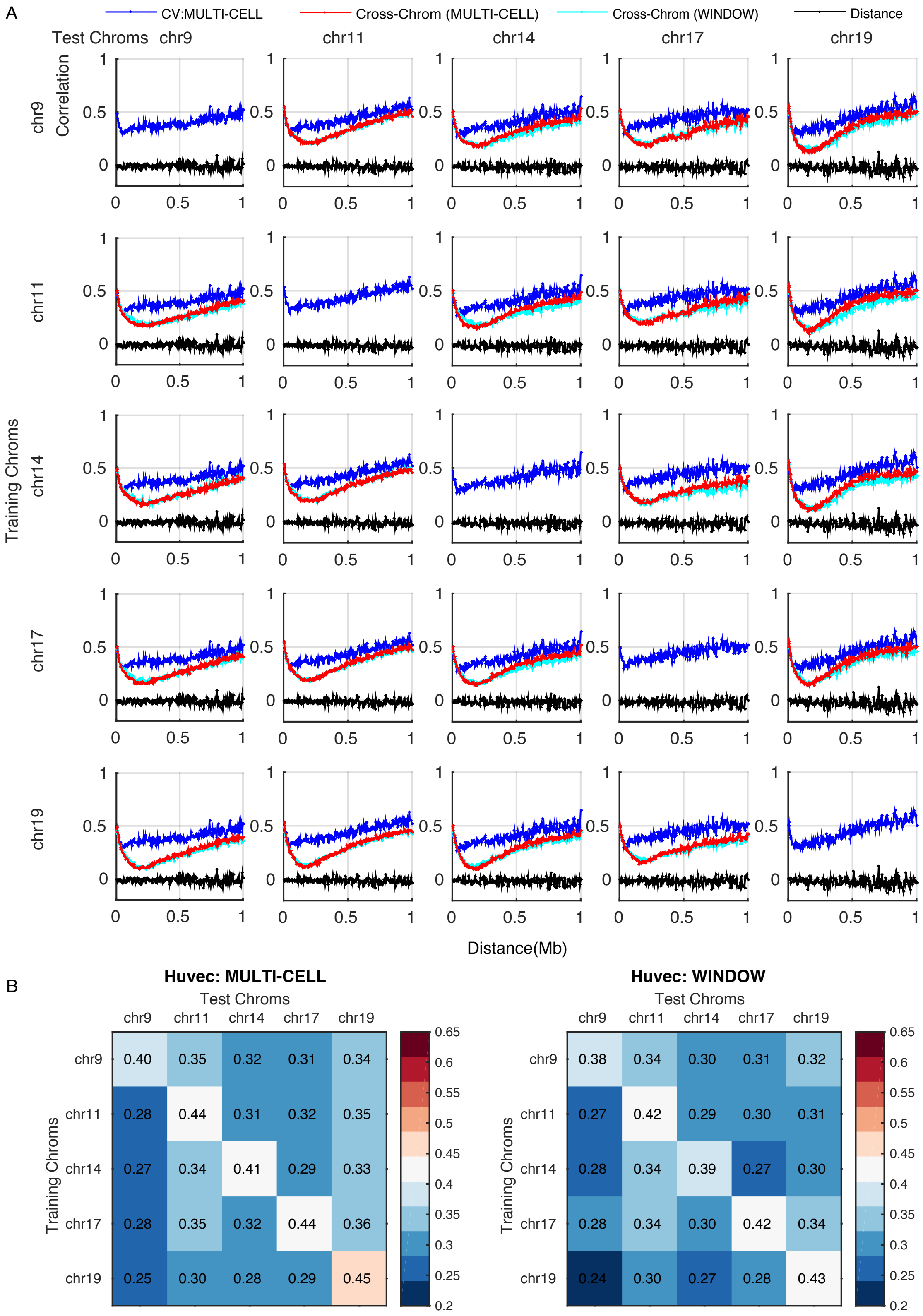

Supplementary Fig 4. Cross-chromosome performance in Huvec. The figure panels follow the same legend as **Supplementary Fig 2**.

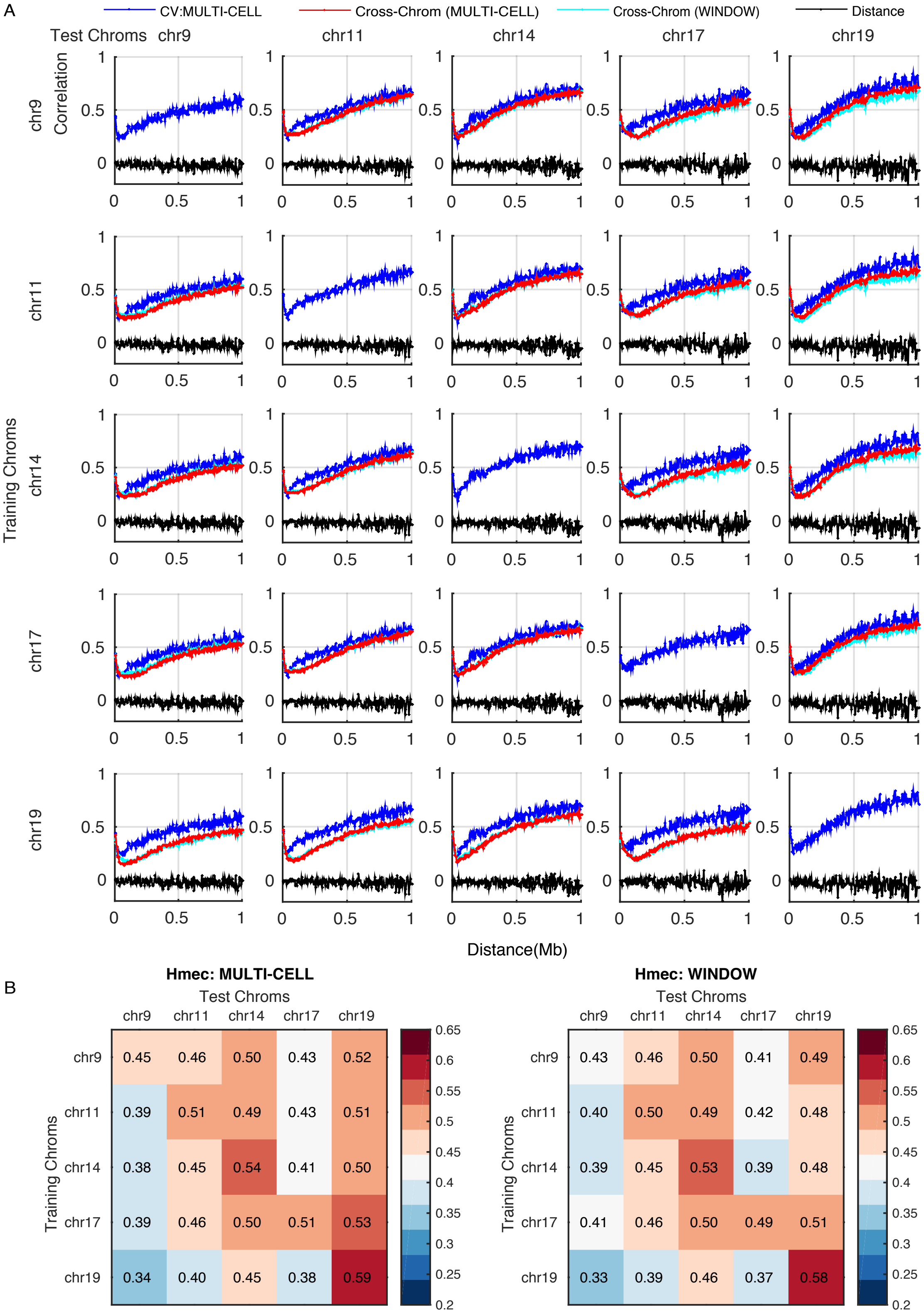

Supplementary Fig 5. Cross-chromosome performance in Hmec. The figure panels follow the same legend as **Supplementary Fig 2**.

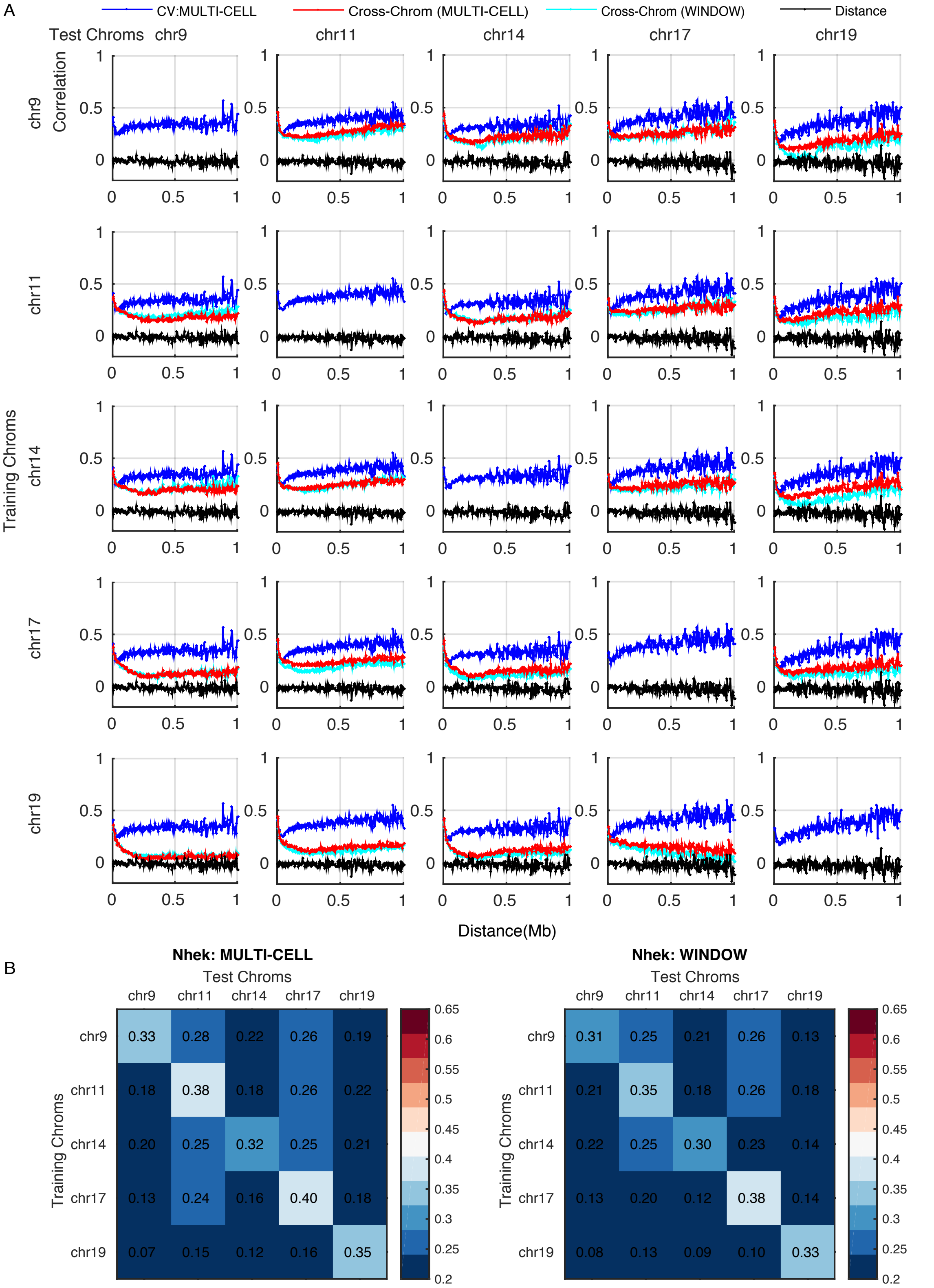

Supplementary Fig 6. Cross-chromosome performance in Nhek. The figure panels follow the same legend as **Supplementary Fig 2**.

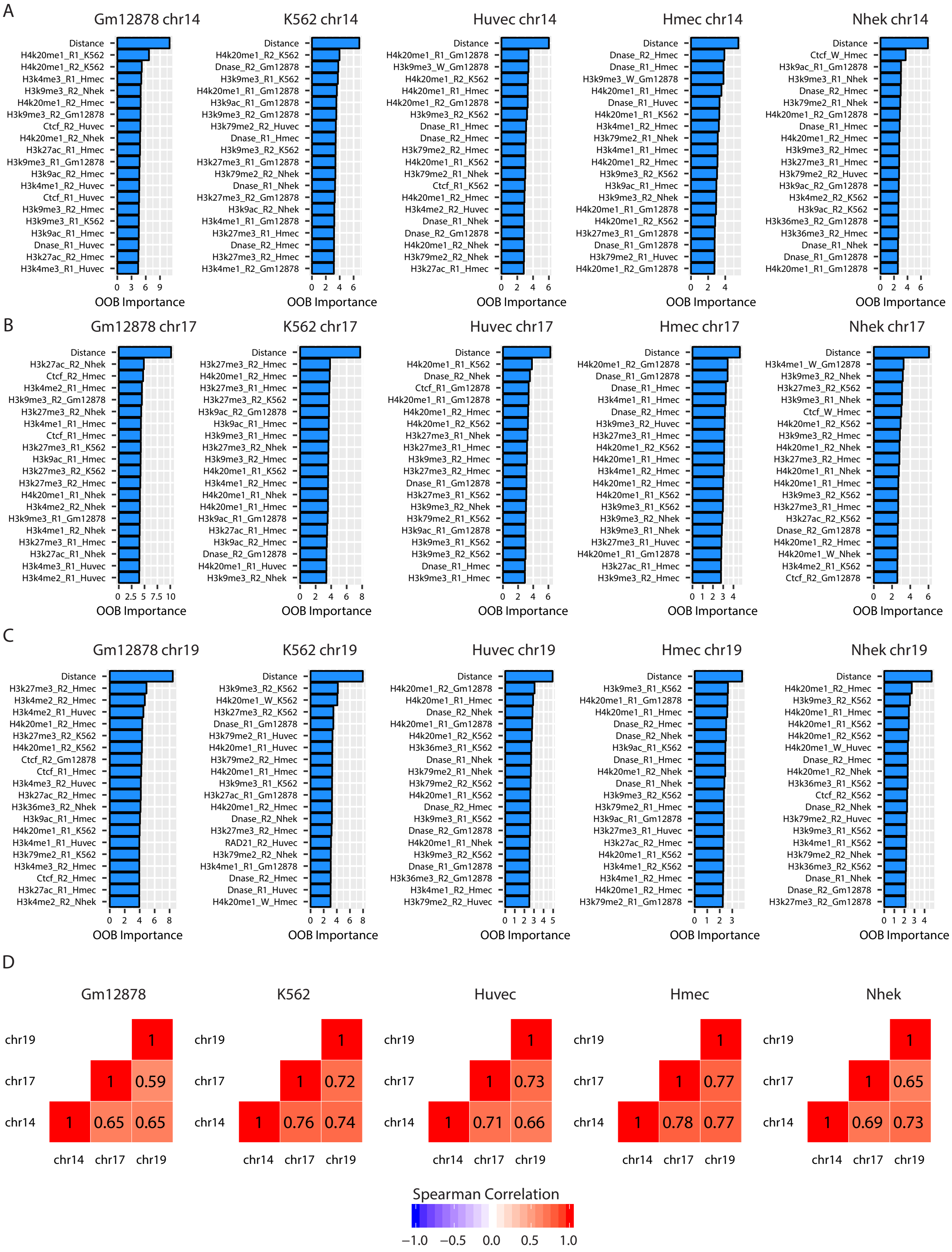

Supplementary Fig 7. Shown are the top 20 features ranked using Out-of-Bag Feature Importance on MULTI-CELL features learned in all five cell lines. Feature importances are computed on three chromosomes: **A.** chromosome 14 **B.** chromosome 17 **C.** chromosome 19. **D.** Heatmap of Spearman's Rank-Order Correlation for feature rankings across chromosome 14, 17 and 19 .

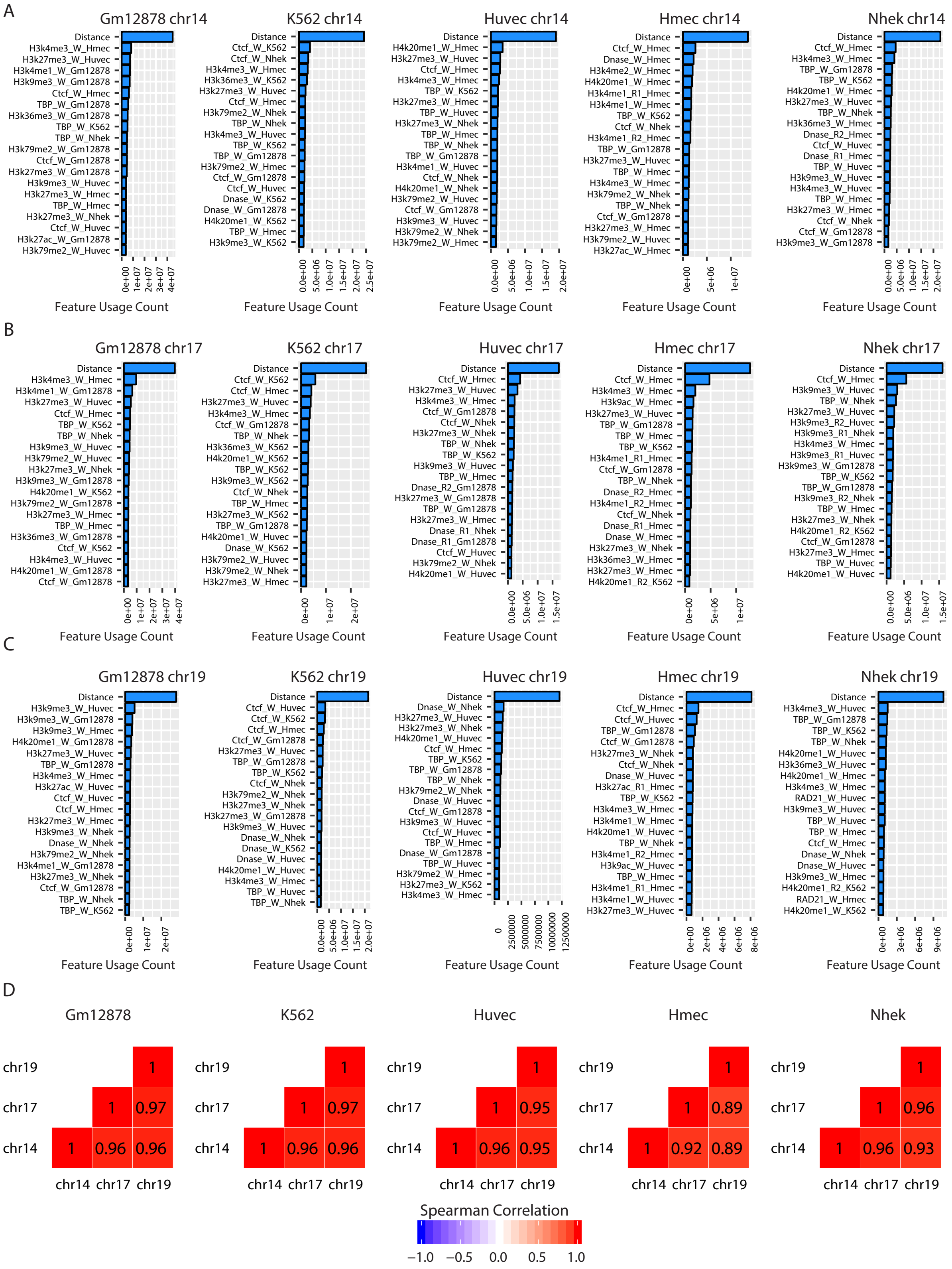

Supplementary Fig 8. Feature ranking of individual features from the MUTLI-CELL feature representation based on counting the number of times a feature is used for predicting test pairs. Shown are top 20 features ranked for all five cell lines on three different chromosomes: **A** chromosome 14, **B** chromosome 17, **C** chromosome 19. **D** Heatmap of Spearman's correlation for feature rankings across chromosome 14, 17 and 19.

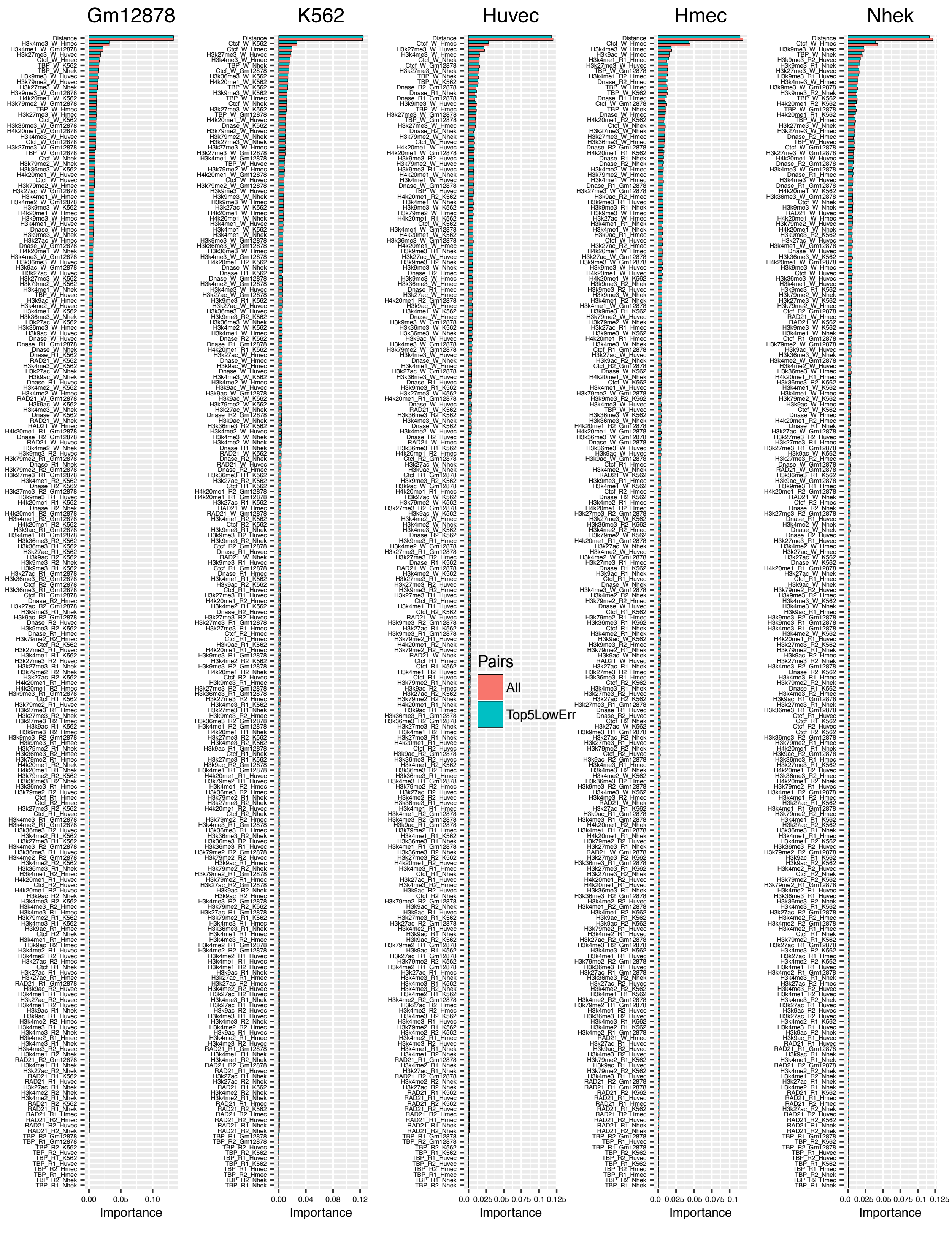

Supplementary Fig 9. Feature ranking based on feature usage counting for all pairs vs top 5% lowest error pairs for all five cell lines. Shown are results for chromosome 17.

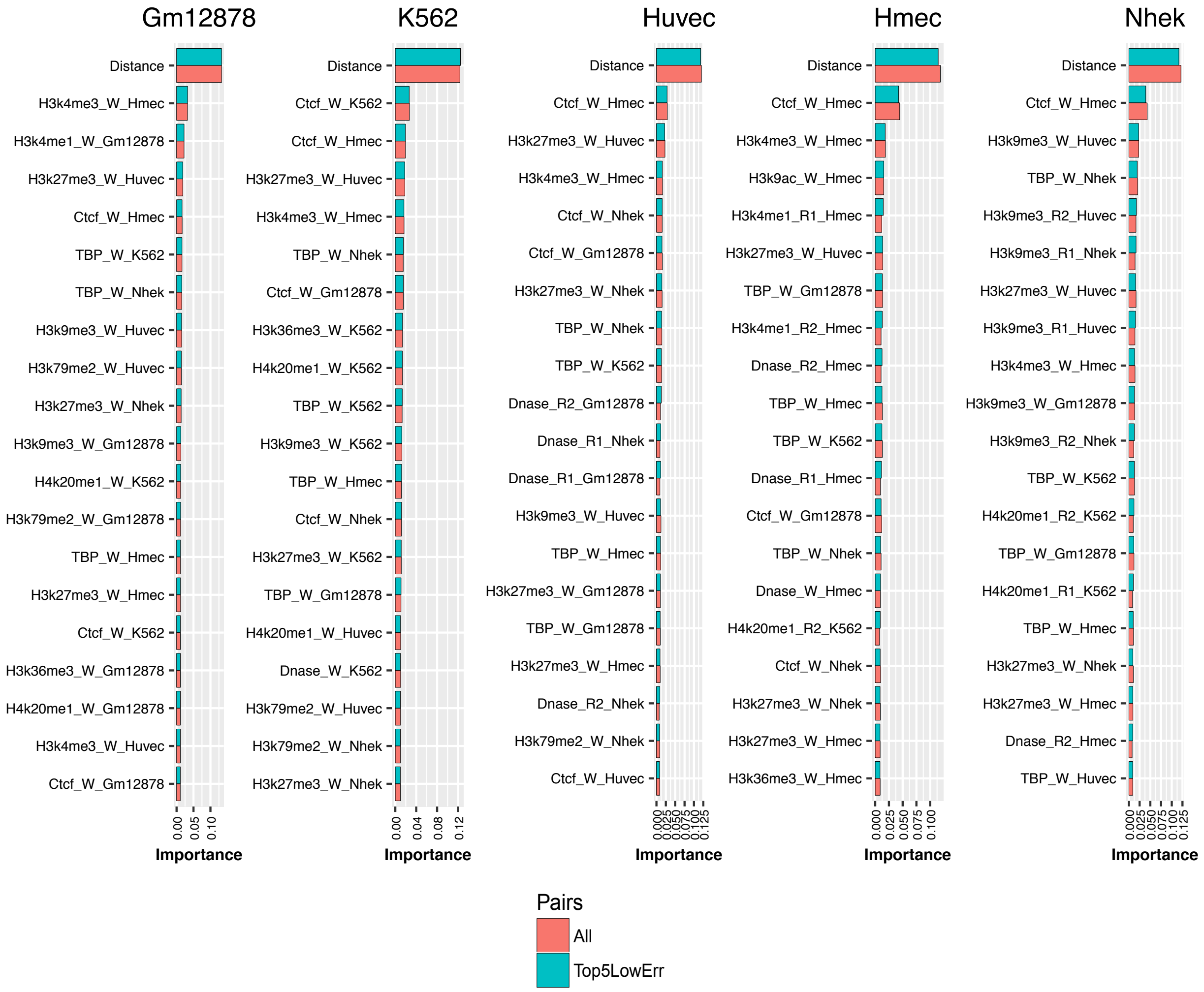

Supplementary Fig 10. Feature ranking of top 20 features identified based on feature usage counting on all pairs vs top 5% pairs on chromosome 17.

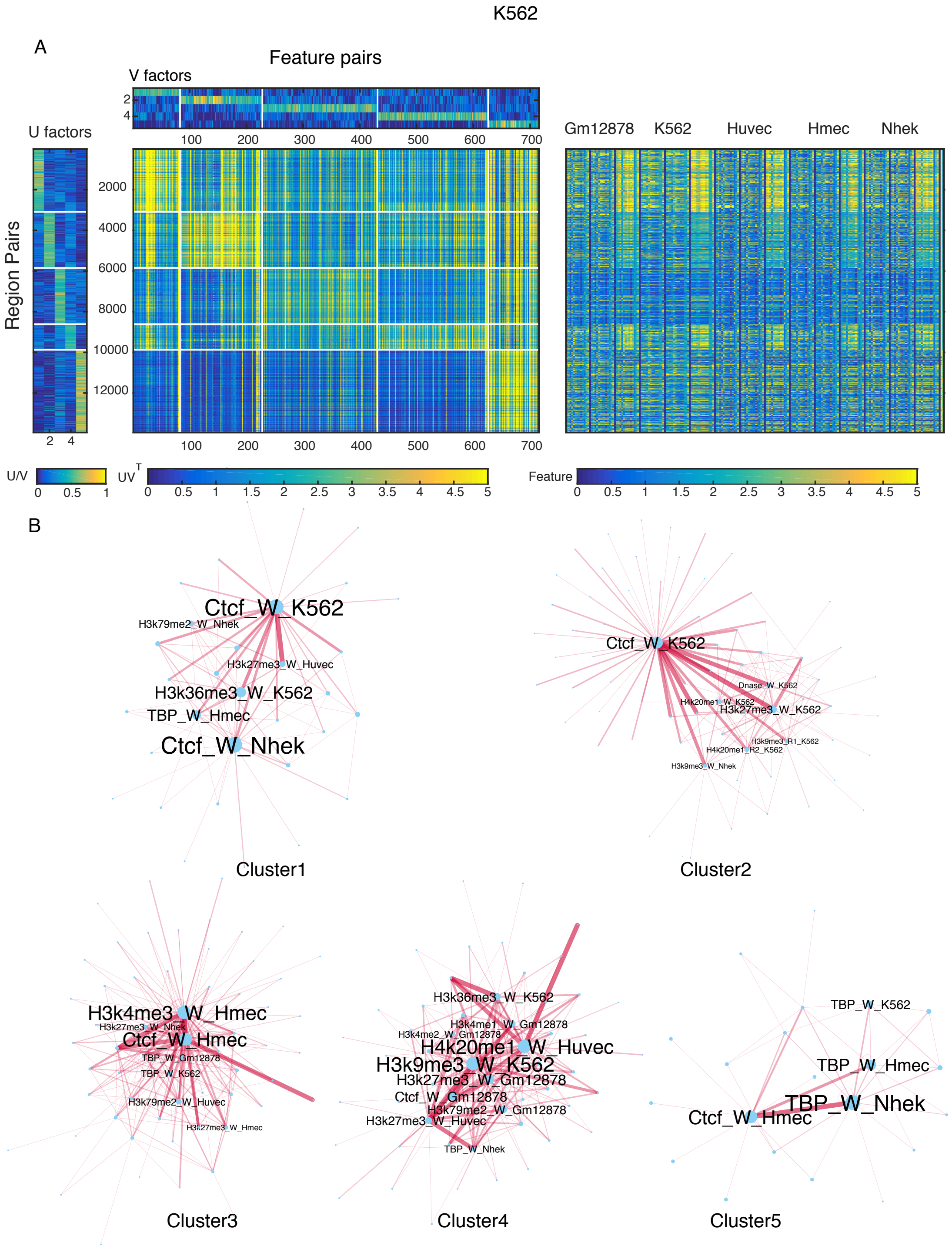

Supplementary Fig 11. NMF analysis of pairs of features in K562. **A.** Non-negative matrix (NMF) factorization of region-pair by feature-pair matrix for K562 chromosome 17. The **U** and **V** factors are the NMF factors to provide membership of region pairs or feature pairs in a cluster (white lines demarcate the region pair and feature pair clusters). The entries in these factors range from 0-1. The factorized feature count matrix is shown below the **V** factors and to the right of the **U** factors. The heatmap on the right are the features associated with each of the pairs, with rows corresponding to a pair of regions and columns corresponding to the feature values grouped by the cell line from which they are obtained. **B.** Cytoscape network representation of important pairs of features for each cluster. The node size is proportional to the number of features the specific feature co-occurs on a path in the regression tree. The thickness of the line is proportional to the number of times the pair of features is used on the path from root to the leaf for a test example pair. Font size of the node label is proportional to its size.

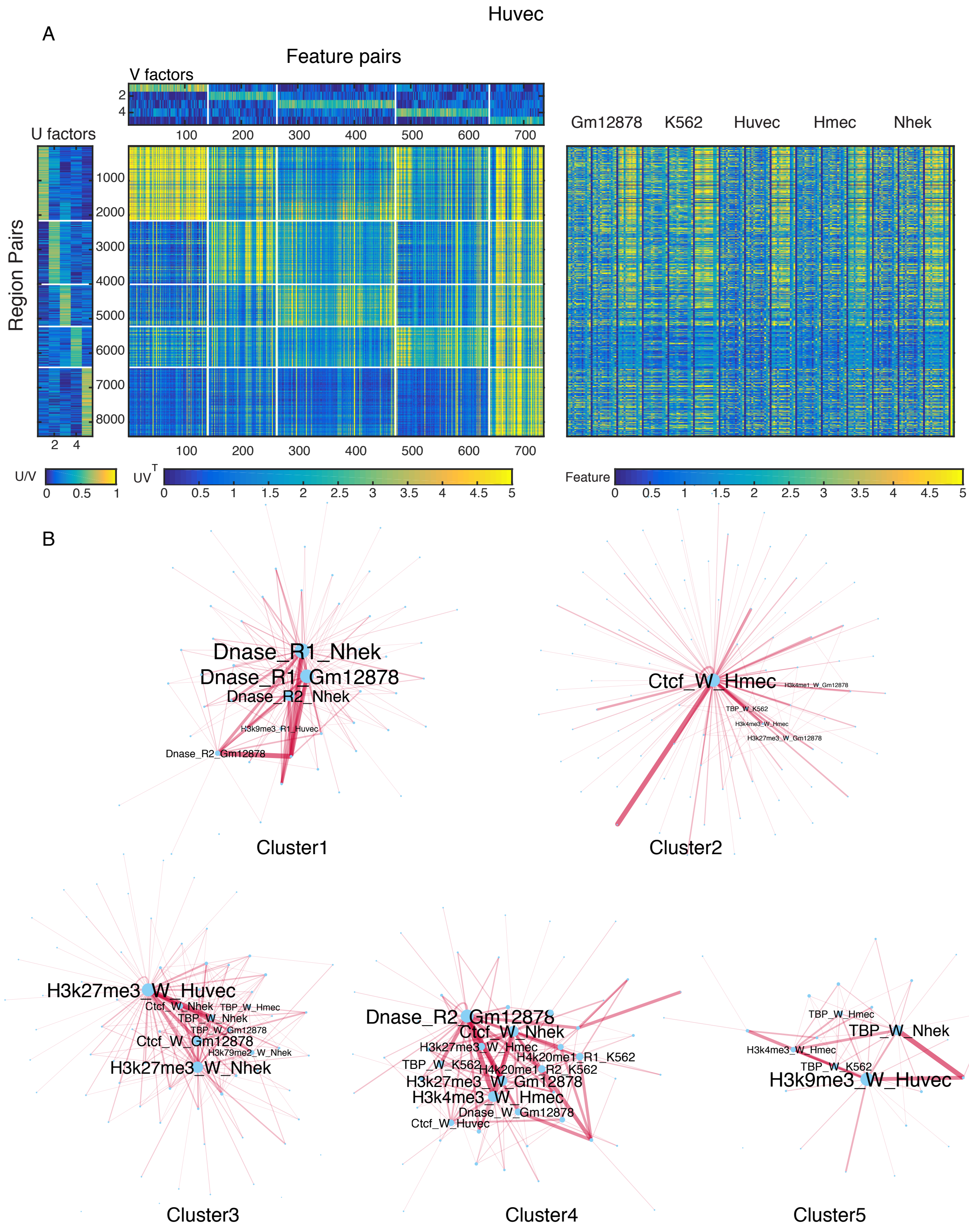

Supplementary Fig 12. NMF analysis of pairs of features in Huvec. The panels follow the same legend as **Supplementary Fig 11.**

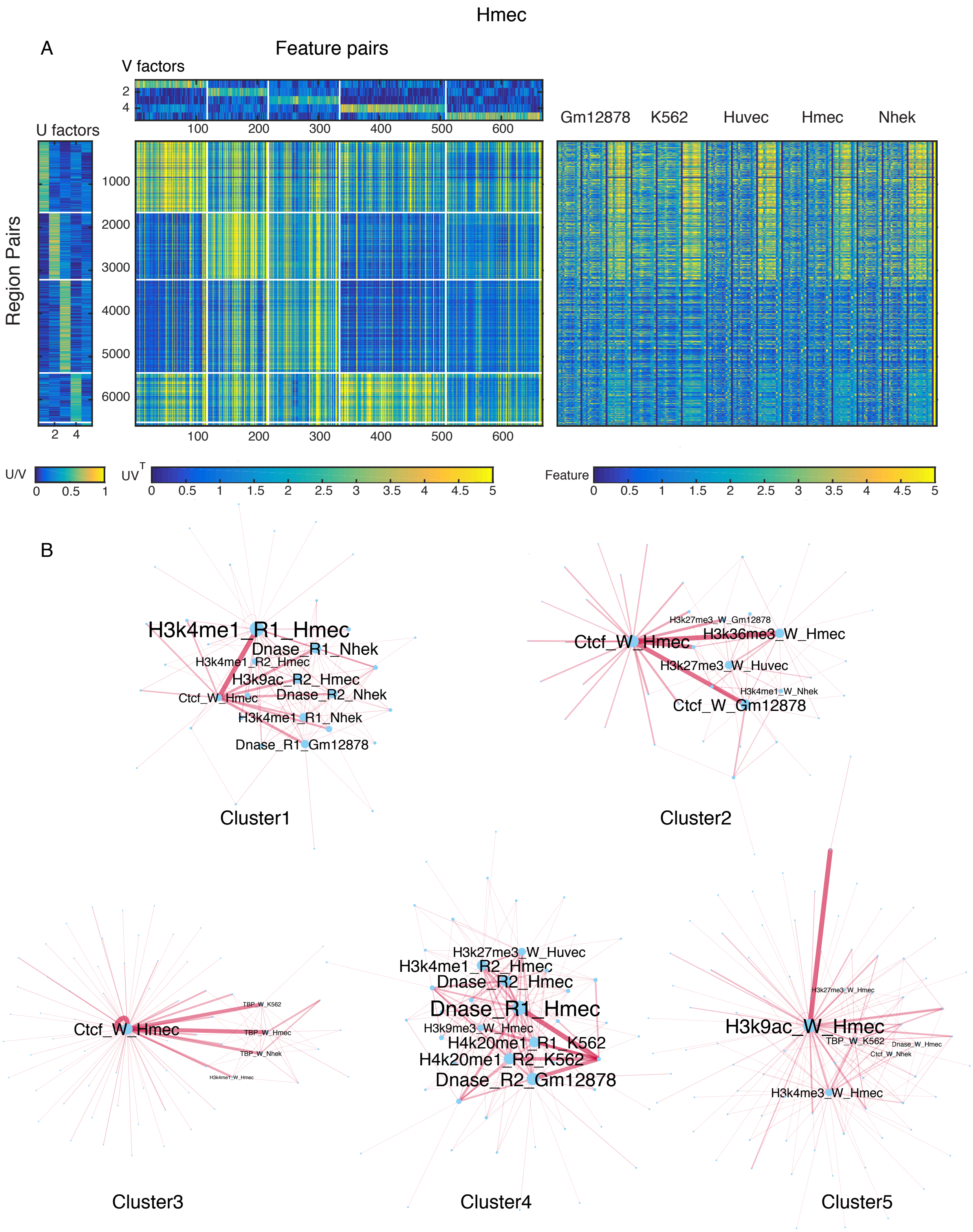

Supplementary Fig 13. NMF analysis of pairs of features in Hmec. The panels follow the same legend as **Supplementary Fig 11.**

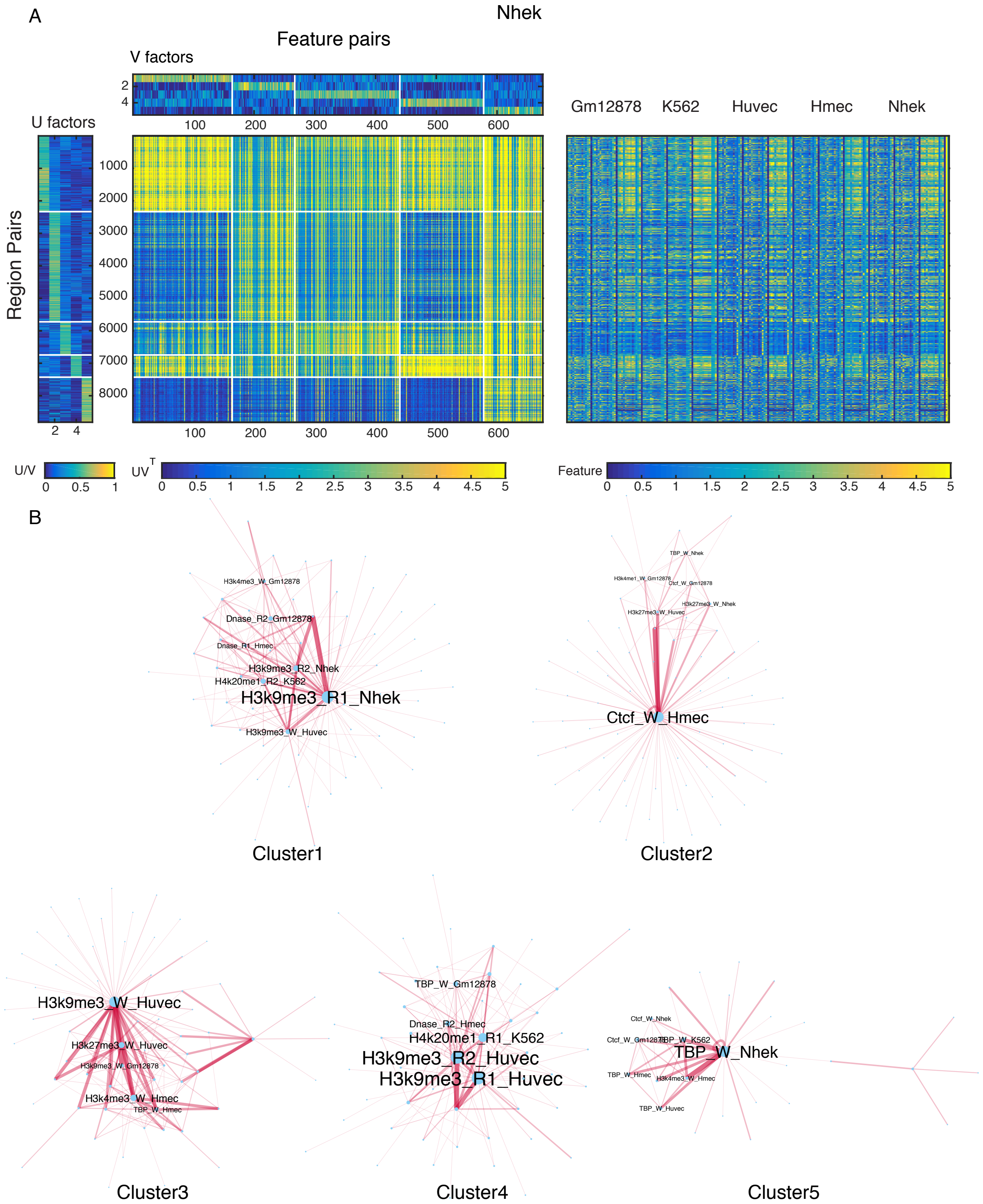

Supplementary Fig 14. NMF analysis of pairs of features in Nhek. The panels follow the same legend as **Supplementary Fig 11.**

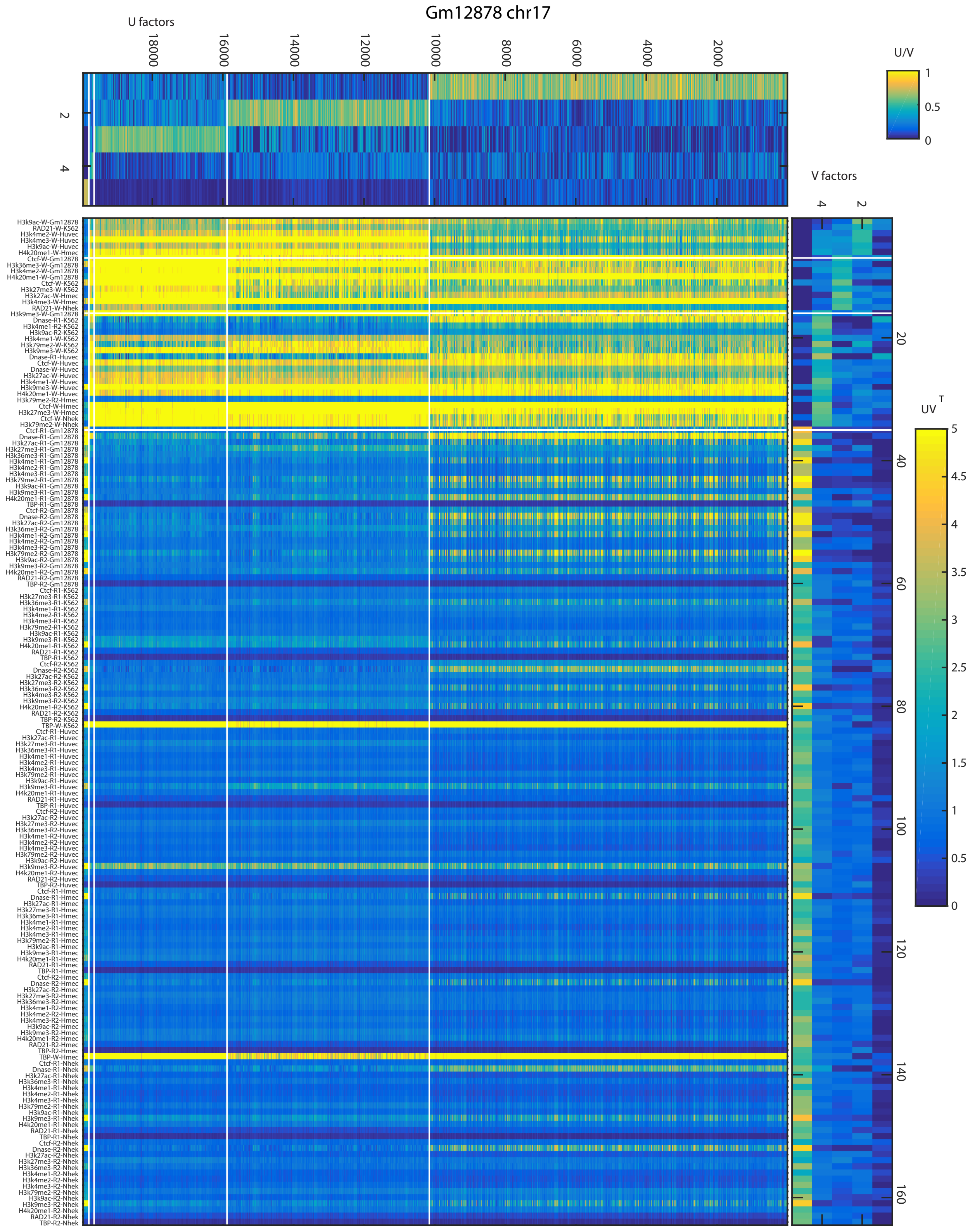

Supplementary Fig 15. NMF analysis of individual feature usage for test pairs in Gm12878, chromosome 17. Shown is the NMF factorized matrix of features (row) by genomic pair (columns). Only pairs with the lowest 5% errors were considered. The **U** and **V** factor heatmaps have values in the range of 0-1 (**U**/**V** colormap). The matrix of feature usage has values in the range of 0-5.

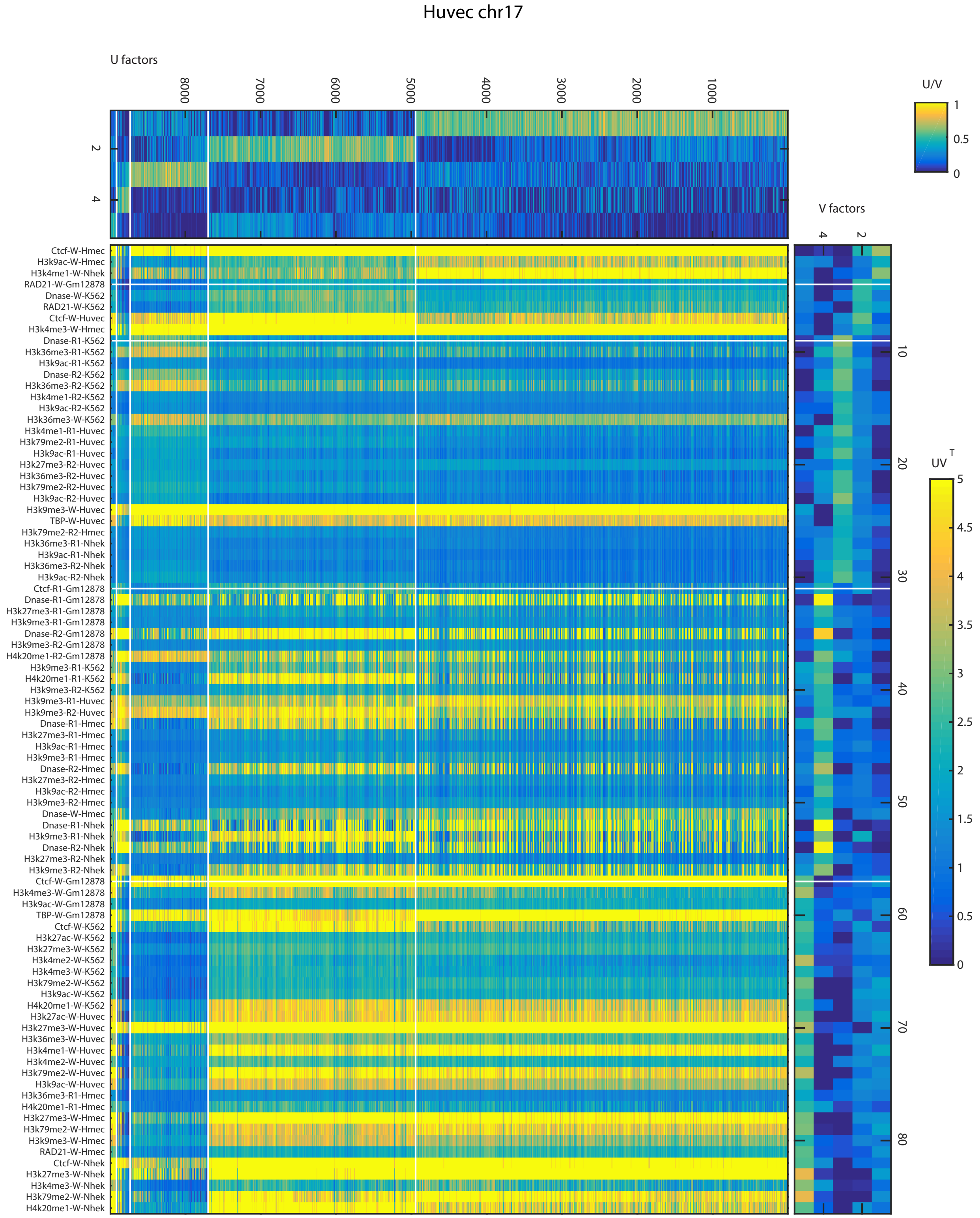

Supplementary Fig 16. NMF analysis of individual feature usage for test pairs in Huvec, chromosome 17. Shown is the NMF factorized matrix of features (row) by genomic pair (columns). Only pairs with the lowest 5% errors were considered. The $\mathbf{U}$ and $\mathbf{V}$ factor heatmaps have values in the range of 0-1 ($\mathbf{U}/\mathbf{V}$ colormap). The matrix of feature usage has values in the range of 0-5.

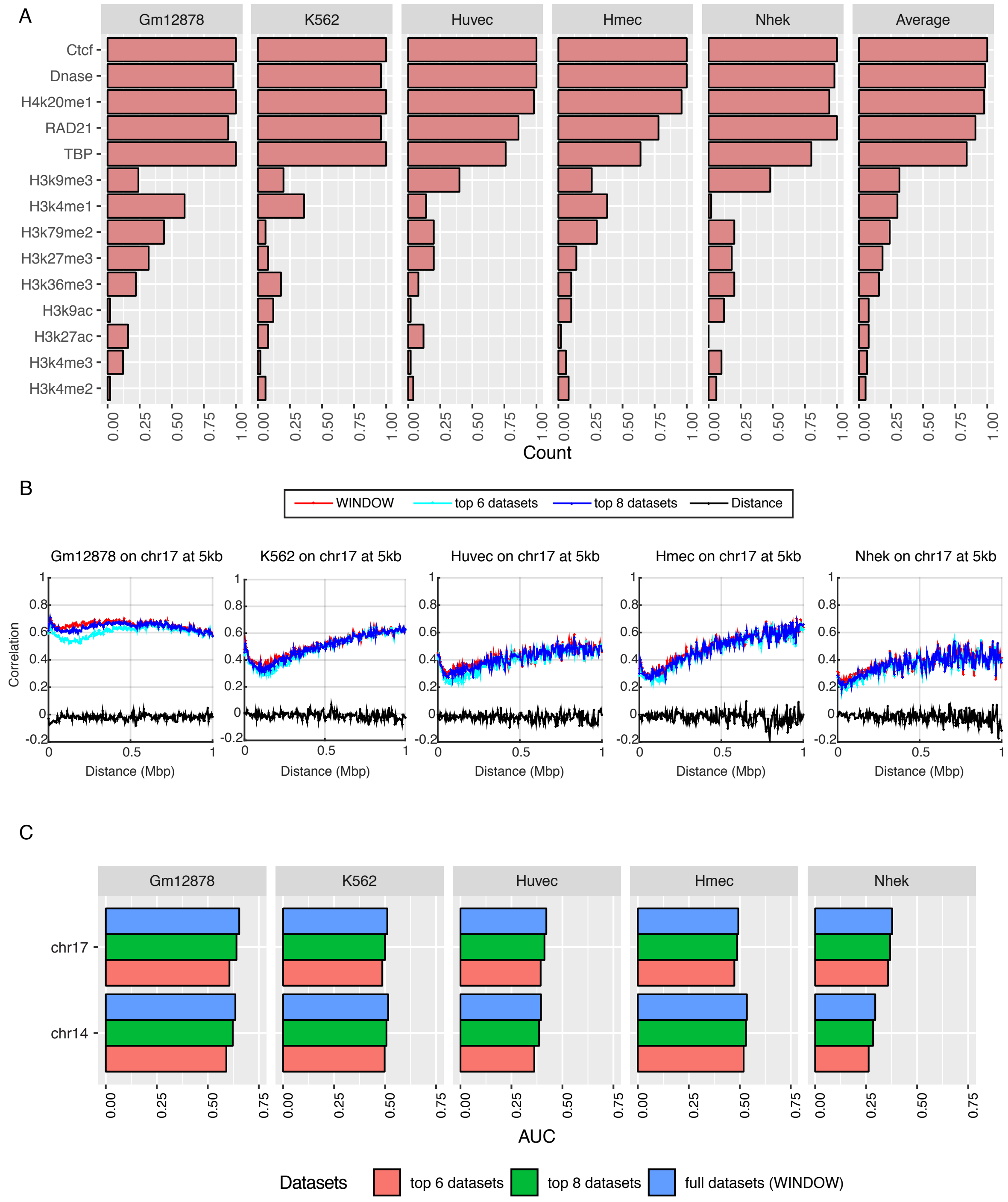

Supplementary Fig 17. Feature selection by MTG-RF comprising Multi-task Group LASSO (MTG-LASSO) followed by a greedy feature refinement approach using Random Forests (RF). **A.** Feature ranking based on the number of times a dataset is selected after applying MTG-RF to all five cells (left) and the average frequency across five cells (right). **B.** The distance-stratified Pearson's correlation plots of chromosome 17 test data when training on the same cell line. Results for all five cell lines is shown for three datasets: top 6 datasets, top 8 datasets and the full set of 14 datasets (WINDOW). **C.** Area under the curve (AUC) of the distance-stratified Pearson's correlation on chromosome 14 and 17 for each of the five cell lines using top 6, top 8 and all 14 (full WINDOW) datasets.

A

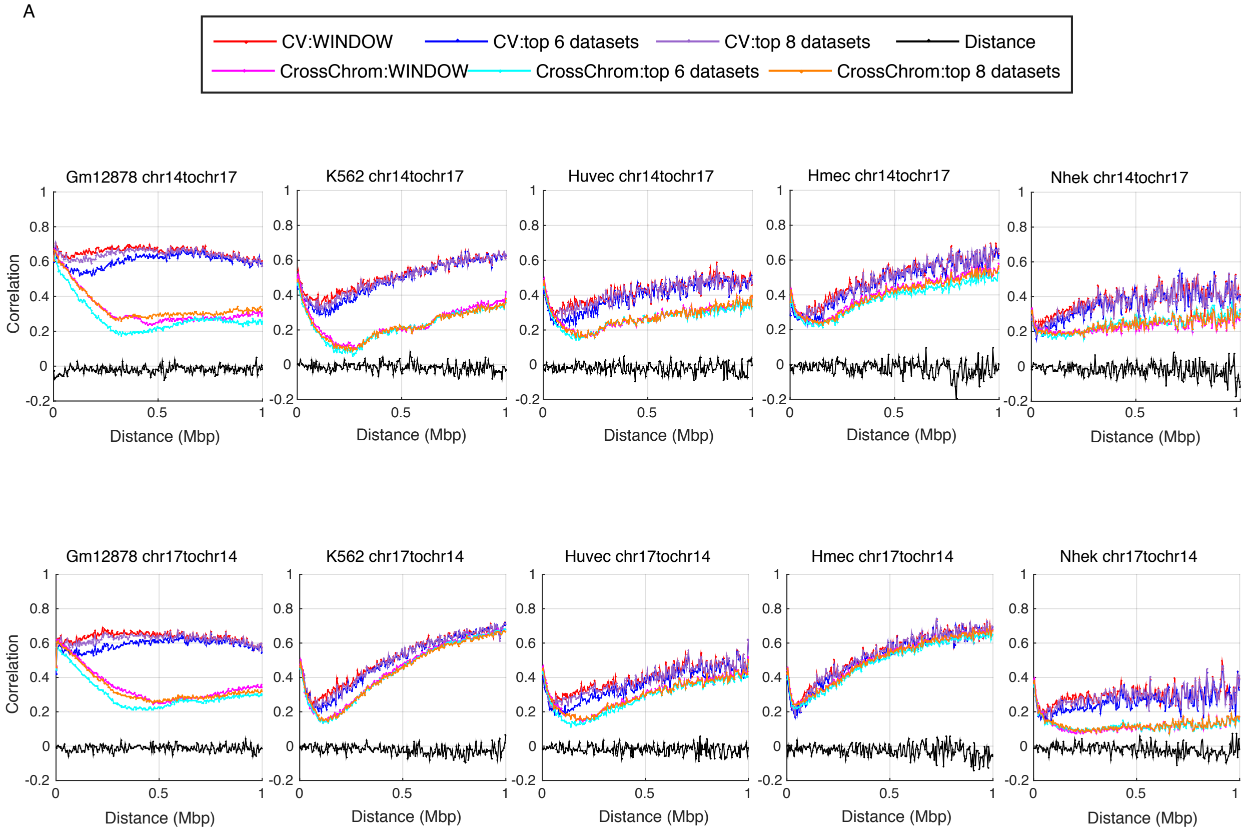

B

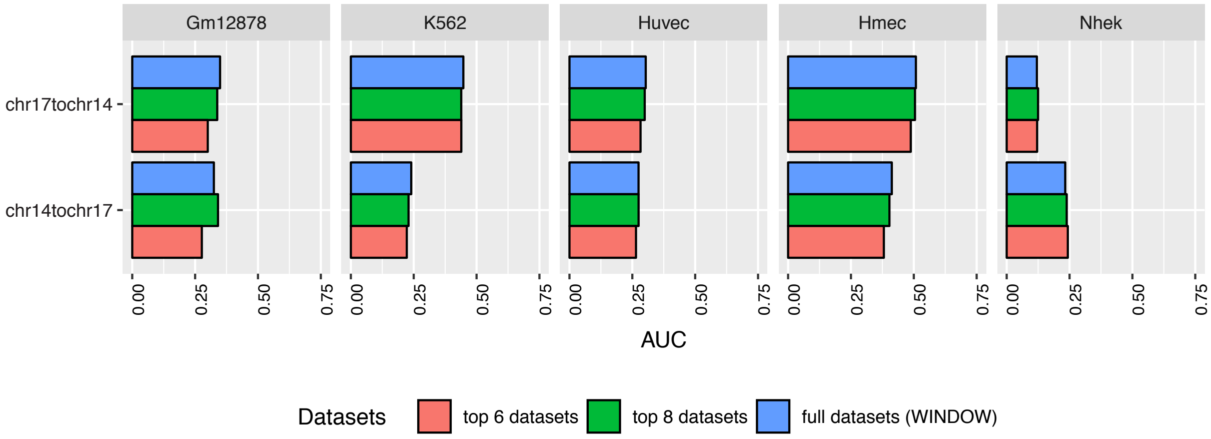

Supplementary Fig 18. Cross-chromosome performance of reduced dataset vs full dataset. **A.** Distance stratified Pearson's correlation plots for same-cell cross-chromosome performance for models trained and tested on one of chromosome 14, 17 at 5kb in five cell lines for three feature sets: top 6 datasets, top 8 datasets and the full set of 14 datasets. **B.** Barplot of AUC for tested cross-chromosome experiments for each of the five cell lines using top 6 datasets, top 8 dataset and full WINDOW dataset.

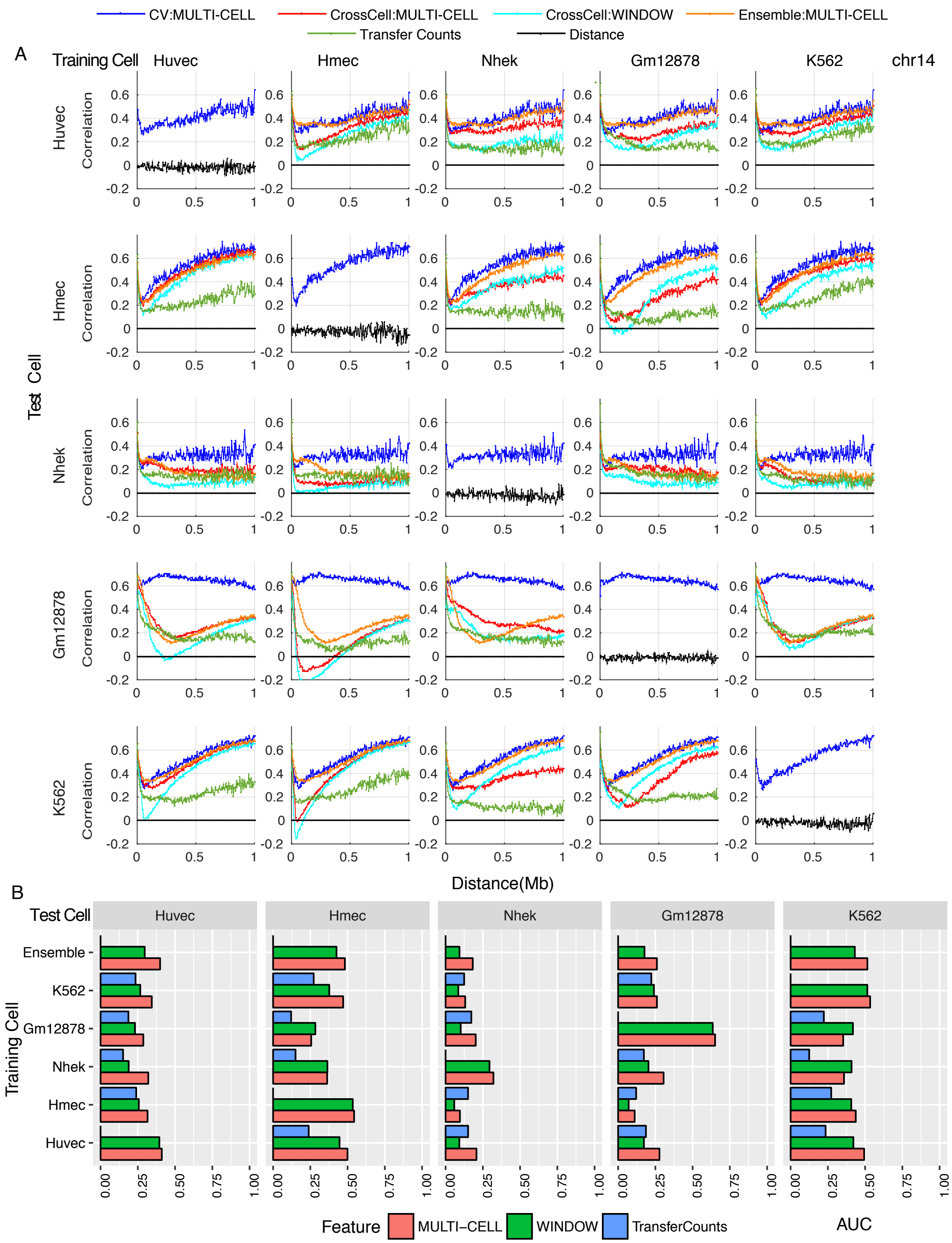

Supplementary Fig 19. Cross-cell performance of HiC-Reg on chromosome 14. **A.** Distance stratified Pearson's correlation plot for cross-cell predictions on chromosome 14. Each row corresponds to the test cell line and the column corresponds to a training cell line. In each sub panel, the lines depict performance using different feature encodings, CV performance using MULTI-CELL features, cross-cell performance using MULTI-CELL and WINDOW features, ensemble performance using MULTI-CELL features, and when transferring counts (TransferCounts). The CV and MULTI-CELL ensemble remain the same for each test cell line (row). **B.** The bar plots show the Area under the distance stratified correlation plot (AUC) for each test cell line using different training cell lines and the ensemble. Both MULTI-CELL and WINDOW features are shown. As an additional baseline we consider the AUC when transferring counts from the training to the test cell line (blue bars).

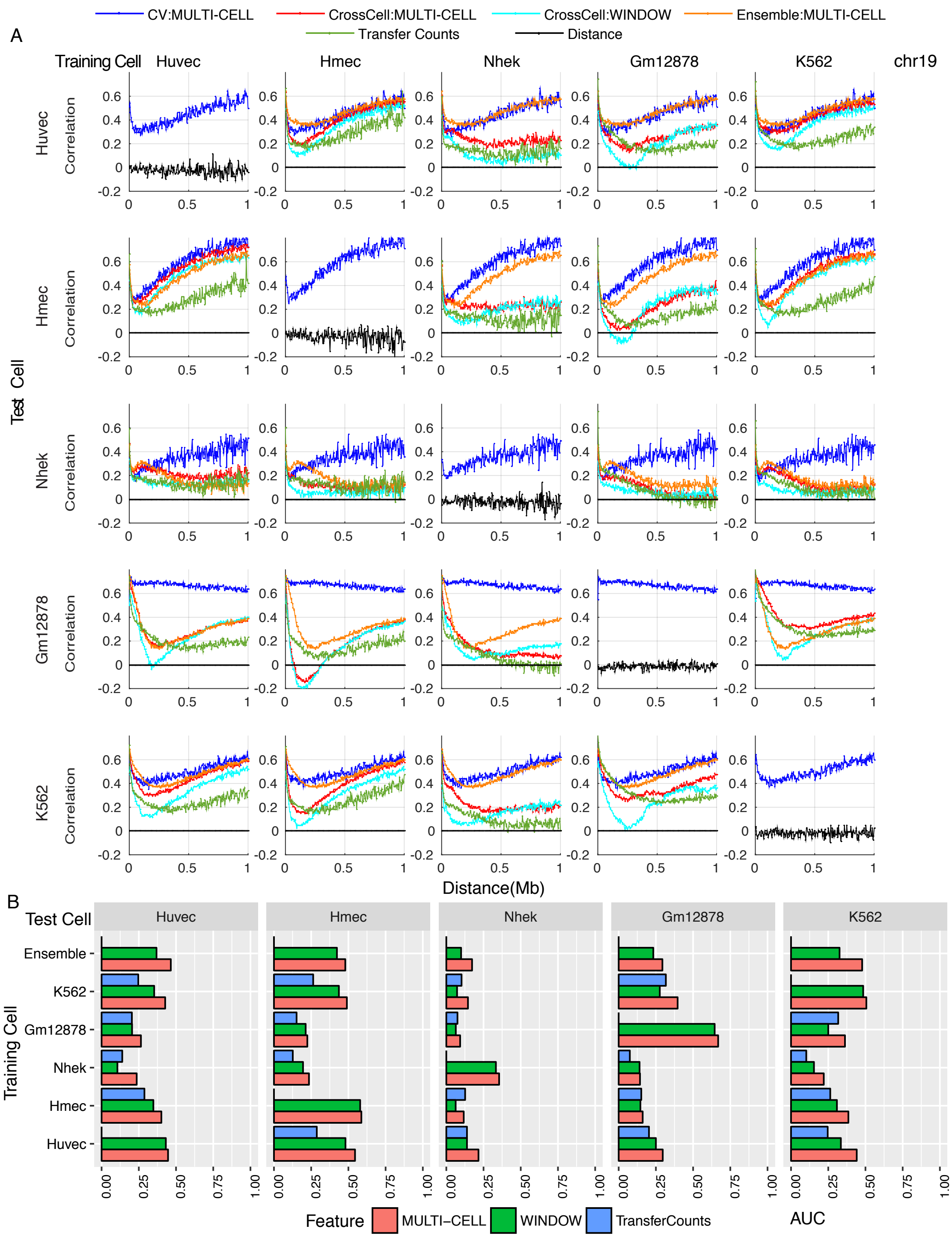

Supplementary Fig 20. Cross-cell performance of HiC-Reg on chromosome 19. **A.** Distance stratified Pearson's correlation plot for cross-cell predictions on chromosome 19. Each row corresponds to the test cell line and the column corresponds to a training cell line. In each sub panel, the lines depict performance using different feature encodings, CV performance using MULTI-CELL features, cross-cell performance using MULTI-CELL and WINDOW features, ensemble performance using MULTI-CELL features, and when transferring counts (TransferCounts). The CV and MULTI-CELL ensemble remain the same for each test cell line (row). **B.** The bar plots show the Area under the distance stratified correlation plot (AUC) for each test cell line using different training cell lines and the ensemble. Both MULTI-CELL and WINDOW features are shown. As an additional baseline we consider the AUC when transferring counts from the training to the test cell line (blue bars).

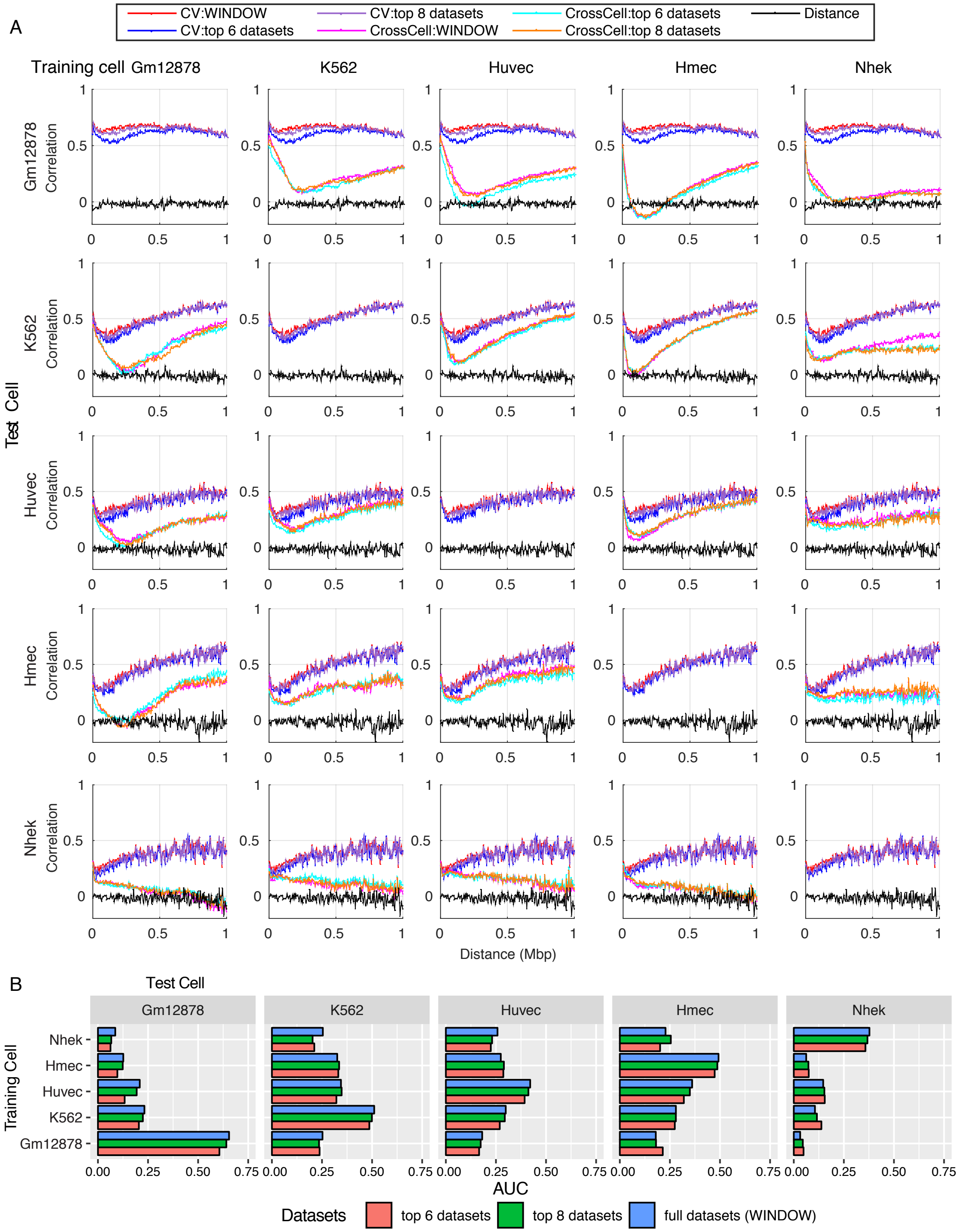

Supplementary Fig 21. Cross-cell performance of features from the reduced dataset vs the full dataset. **A.** Distance stratified Pearson's correlation plots using models trained on one cell line (columns) and tested on a different cell line (rows) for chromosome 17 and all five cell lines: Gm12878, K562, Huvec, Hmec, Nhek. Each sub panel (except the ones on the diagonal) show the distance stratified correlation curves of CV and Cross-Cell performance using WINDOW features for three datasets: top 6, top 8 and the full set of 14 datasets. The plots on the diagonal show the CV performance for all three datasets. **B.** The bar plots show the AUCs for different training cell line models in chromosome 17 for three datasets: top 6, top 8 and the full 14 datasets.

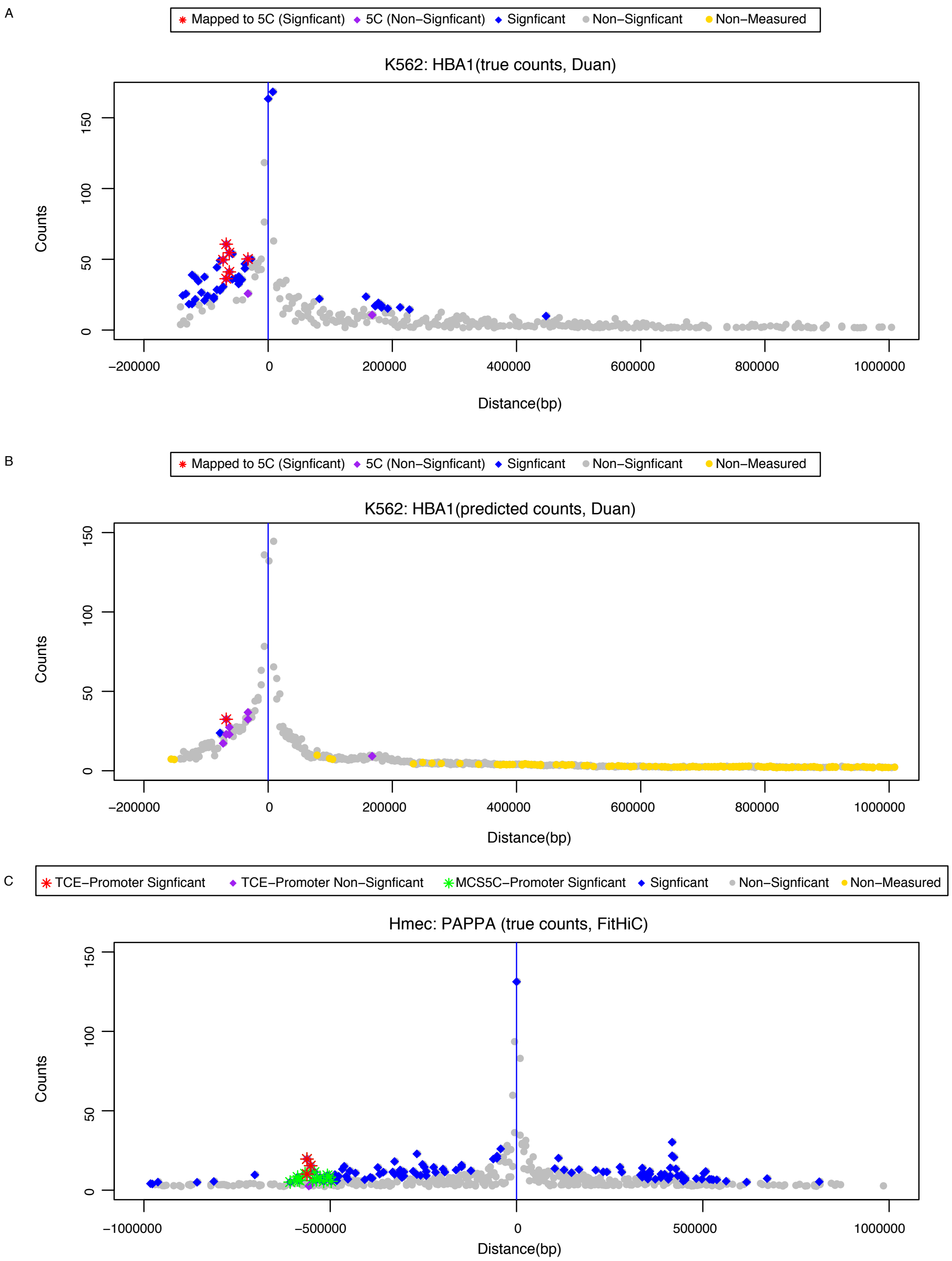

Supplementary Fig 22. Shown are Manhattan plots of significant interactions identified when using true and predicted counts around the *HBA1* and *PAPPA* loci. **A.** Manhattan style plots of true interaction counts in K562 centered around the *HBA1* gene promoter. Interactions associated with both bins spanning *HBA1* promoter are shown. The red asterisk corresponds to significant interactions overlapping 5C regions. Blue diamonds denote significant interactions identified by Duan et al. Purple diamonds correspond to non-significant pairs overlapping 5C regions. **B.** Manhattan style plots of predicted counts using K562 CV model centered around the *HBA1* gene promoter. The color of each marker type follows the same legend as **A**. See **Fig 8C** for comparison to Fit-Hi-C. **C.** Manhattan style plots of true counts in Hmec around the *PAPPA* gene locus. Interactions associated with both bins spanning *PAPPA* promoter are shown. Red asterisk corresponds to significant interactions between the TCE and *PAPPA* promoter. Green asterisk corresponds to significant interactions between the MCS5C and *PAPPA* promoter. Blue diamonds correspond to significant interaction counts identified using Fit-Hi-C.

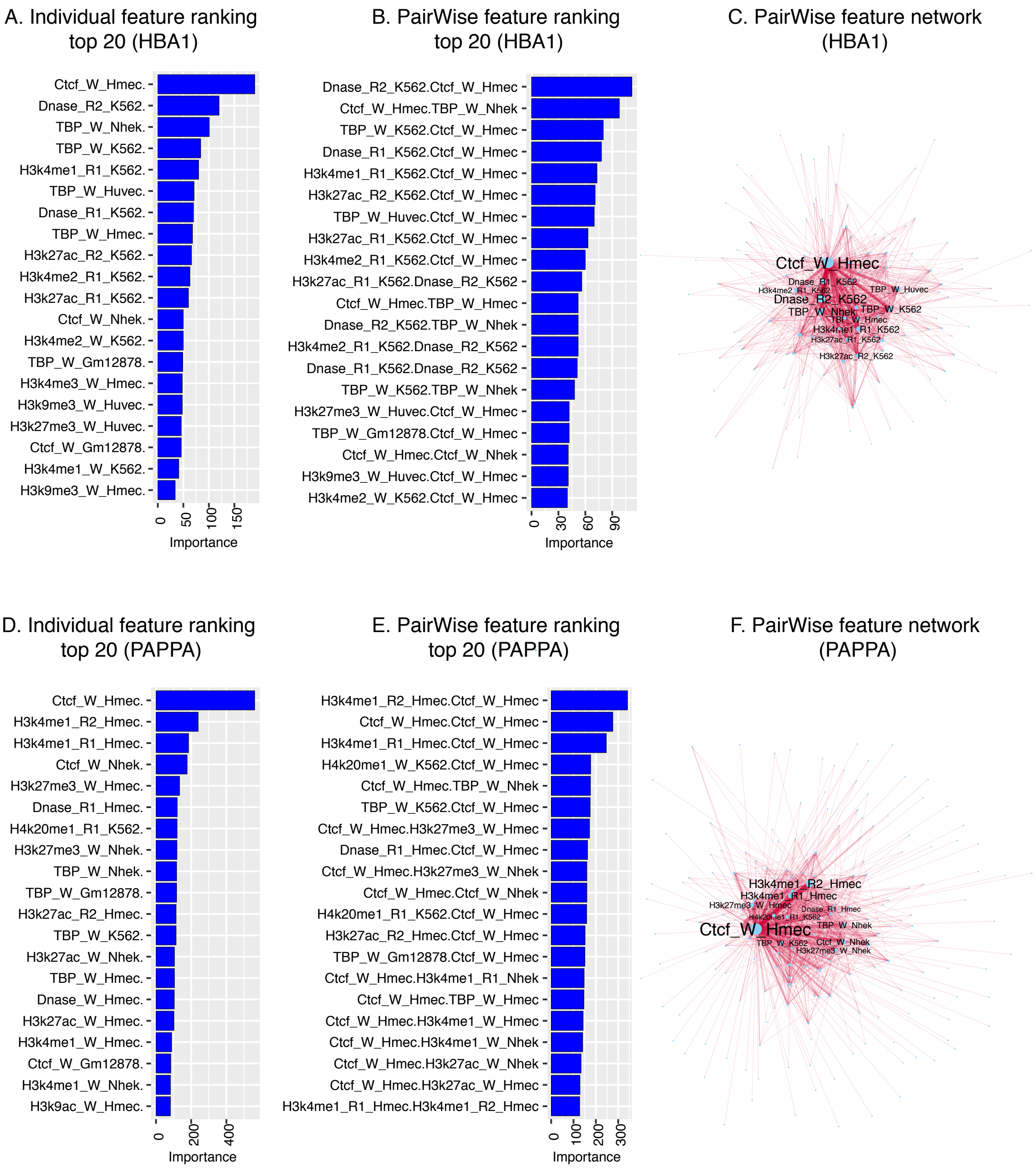

Supplementary Fig 23. Feature ranking based on counting the usage of individual and pairs of features in significant interactions associated with well-studied loci. **A.** Top 20 features identified with significant interactions of the *HBA1* locus. **B.** Top 20 pairs of features for significant interactions with the *HBA1* locus. **C.** Network representation of pair-wise features for significant interactions with the *HBA1* locus. **D.** Top 20 features associated with significant interactions for the *PAPPA* gene. **E.** Top 20 pairs of features associated with the *PAPPA* gene. **F.** Network representation of pair-wise features for significant interactions with the *PAPPA* gene.

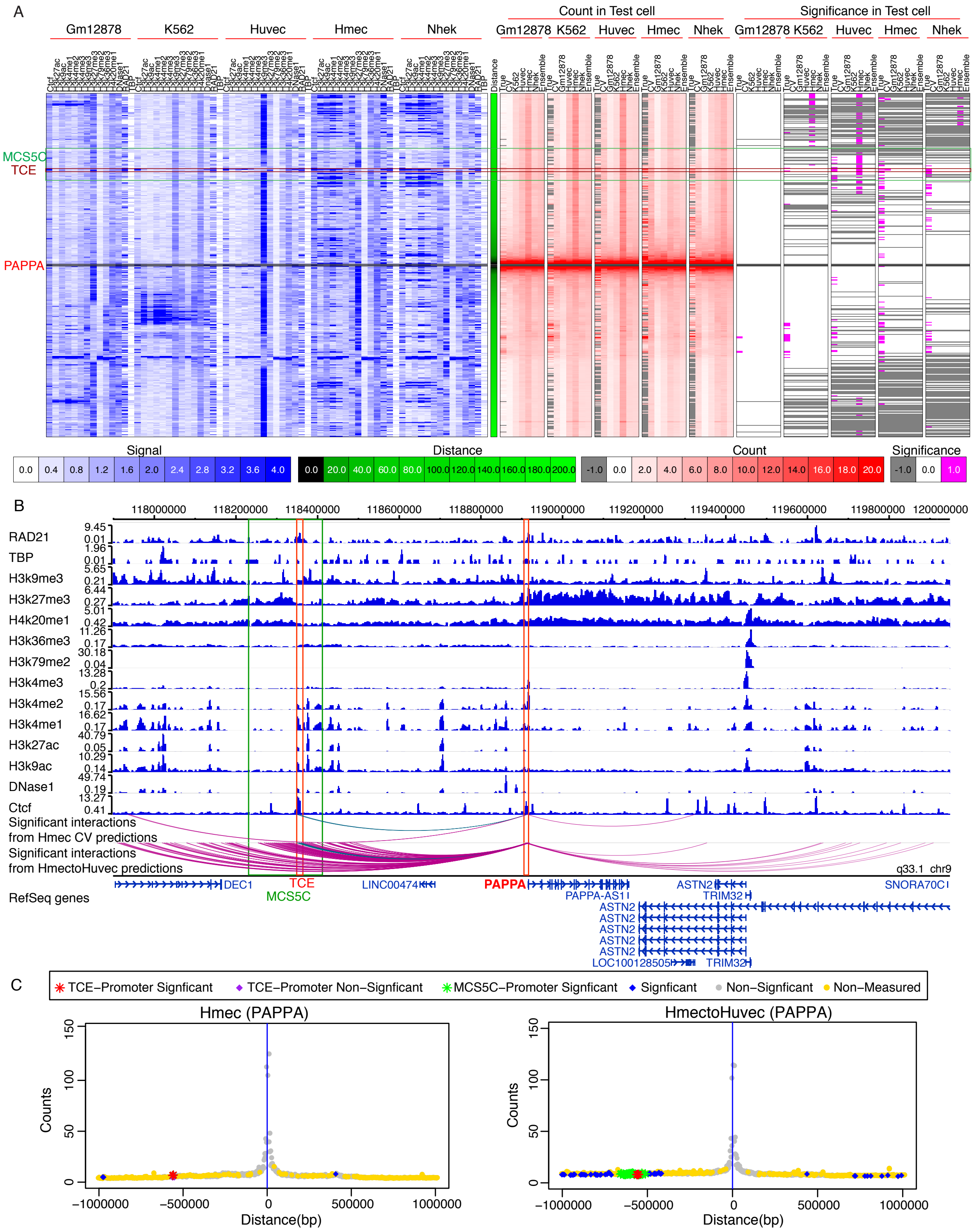

Supplementary Fig 24. Visualization of interactions associated with *PAPPA* gene. **A.** Visualization of

feature values, true counts as well as predictions from 30 different models for regions in the 1Mb radius of the *PAPPA* gene promoter (Similar to **Figure 8**). The features are shown in the white-blue heatmap with Distance shown as a separate column (colormap 0:0kb and 200:1Mb), the predicted counts are in the red-white heatmap and the significance is depicted by the white-magenta heatmap. Gray entries in the white-magenta heatmap corresponds to pairs that do not have a measured count in the original Hi-C data. The MCS5C region is demarcated by the green horizontal lines, the TCE is depicted by dark red lines and the *PAPPA* gene is shown in red. The test cell lines are mentioned above the red line, while the column names (vertical orientation) are for the training models. **B.** Visualization of feature signals using WashU Epigenome Browser for significant interactions. The displayed feature signals are from the Hmec cell line. Majority of the interactions were from the Hmec cell line. The interactions in teal are those spanning the TCE locus, while the magenta interactions are spanning other region bins. **C.** Manhattan plots of predicted counts using the Hmec CV model and predicted counts in Huvec using a model trained on Hmec. Red and green asterisks denote interactions of *PAPPA* promoter with the TCE or the MCS5C region, respectively. Blue diamonds denote significant interactions. Yellow dots do not have measured counts in the original Hi-C data. Significant interactions were called using Fit-Hi-C. Interactions associated with both bins spanning *PAPPA* promoter are shown.

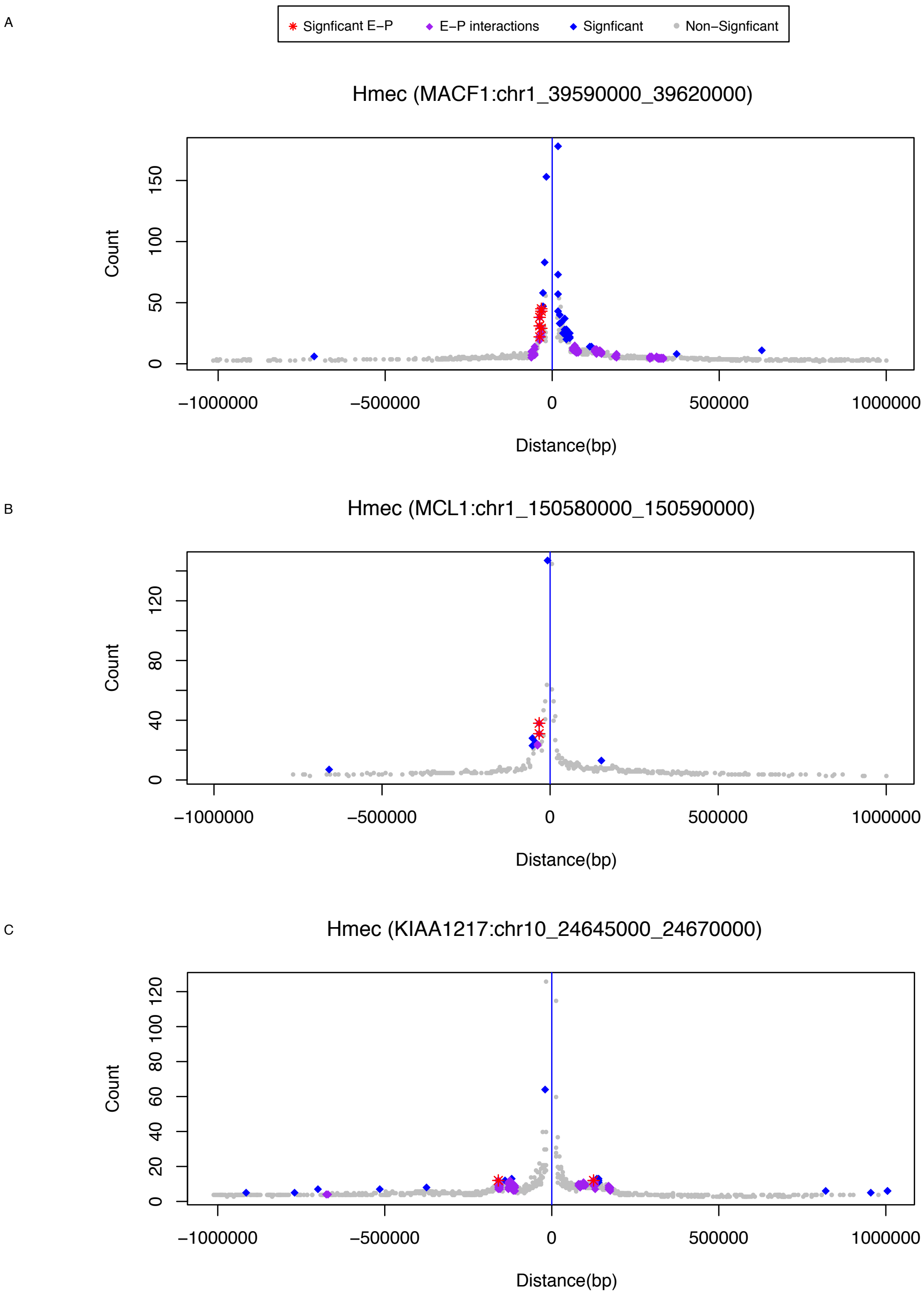

Supplementary Fig 25. Shown are Manhattan style plots of predicted counts around enhancer coordinates that have been shown to regulate the expression of a specific gene. The coordinates of the enhancer and the gene name is listed on top of each Manhattan plot. A red asterisk denotes a significant interaction between a promoter bin and the 5kb bins associated with the enhancer. In some cases (e.g., *MCL1*), the enhancer spans more than one 5kb bin, but this is not depicted in the plot. Purple diamonds denote all 5kb bins that contain a TSS and have a non-zero interaction with one of the 5kb enhancer bins. The blue diamonds depict significant interactions associated with the enhancer, but not necessarily overlapping a TSS bin. Gray dots are not significant. Only predictions from CV trained models were considered and cell lines with significant interactions are shown. **A.** *MACF1*. **B.** *MCL1*. **C.** *KIAA1217*.

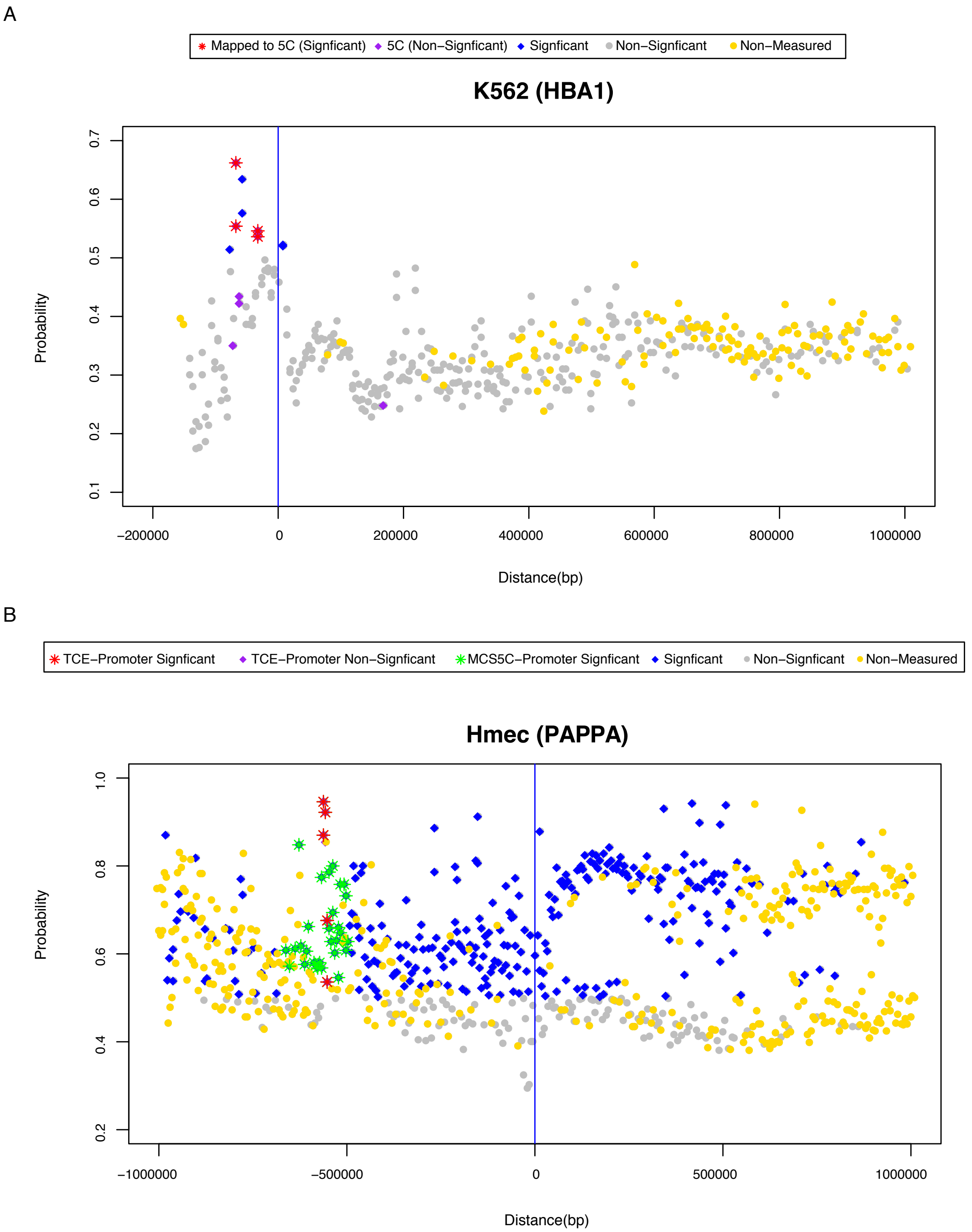

Supplementary Fig 26. Application of a classification approach to identify interactions with the *HBA1* and *PAPPA* genes. Shown are Manhattan style plots centered around the 5kb bins spanning the *HBA1* and *PAPPA* gene promoter. The y-axis corresponds to probability of an interaction. **A.** Predicting interactions with *HBA1* using a classifier trained in K562. Red asterisk denotes an interaction of *HBA1* promoter with one of the 5C regions. Blue diamonds denote significant interactions (probability >0.5). Yellow dots do not have measured counts in the original Hi-C data. **B.** Predicting interactions for the *PAPPA* gene using a classifier trained in the Hmec cell line. Red asterisks correspond to significant interactions between TCE and the *PAPPA* promoter. Green asterisks are significant interactions between the MCS5C bins and *PAPPA* promoter. Blue diamonds denote significant interacting pairs (probability >0.5). Yellow dots do not have measured counts in the original Hi-C data.

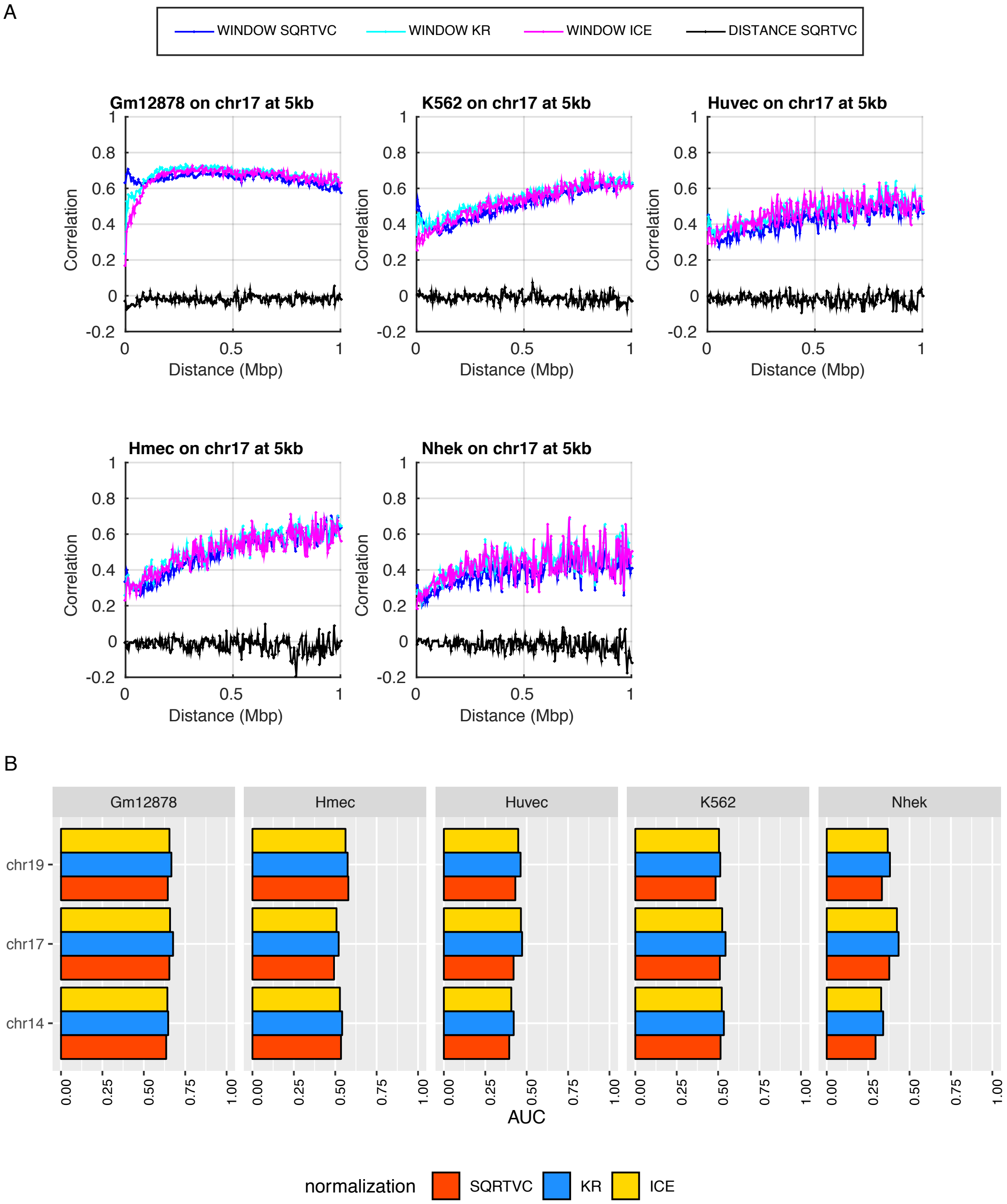

Supplementary Fig 27. Cross-validation (CV) performance of HiC-Reg when trained and tested in the same chromosome (chr17) in different cell lines based on WINDOW features using Hi-C matrix normalized with three different methods: Square root of Vanilla coverage (SQRTVC), Iterative Correction and Eigen vector decomposition (ICE) and Knight-Ruiz (KR). **A.** Distance stratified Pearson's correlation curve assessing predictive performance of HiC-Reg trained on data normalized with three different normalization methods. **B.** Area under the distance stratified correlation curve (AUC) for three different chromosomes using the different normalization methods.

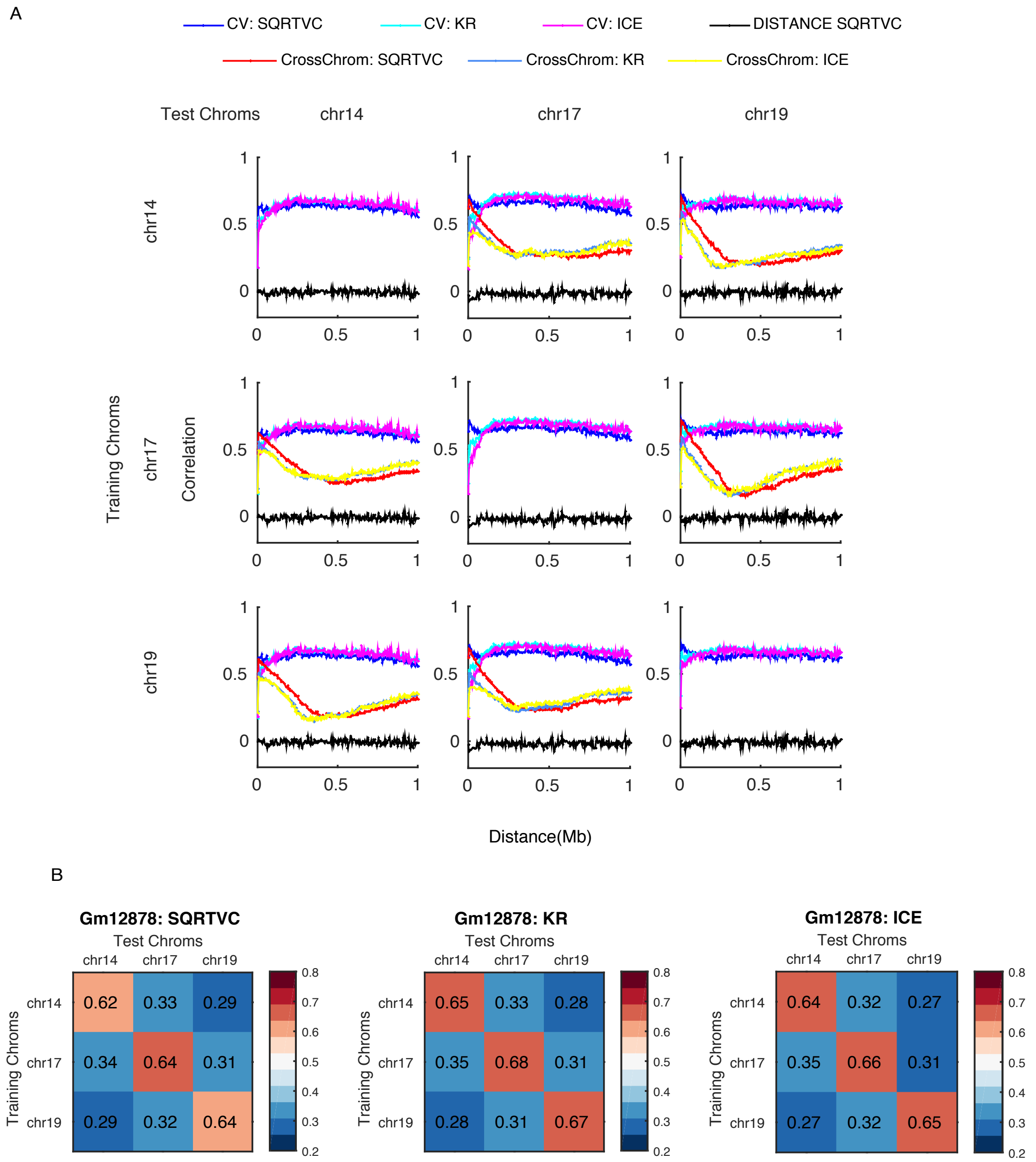

Supplementary Fig 28. Cross-chromosome performance across three chromosomes in the Gm12878 cell line based on WINDOW features when using Hi-C data from three different normalization methods: Square root of Vanilla coverage (SQRTVC), Iterative Correction and Eigen vector decomposition (ICE) and Knight-Ruiz (KR). **A.** Shown are the distance stratified Pearson's correlation curves when training on the row chromosome and testing on the column chromosome. The curves in the diagonal positions correspond to CV performance. The CV lines are the same in each column. **B.** Heatmap of Area under the distance stratified correlation curve (AUC) for the CV and cross-chromosome experiments across the three chromosomes in the Gm12878 cell line.

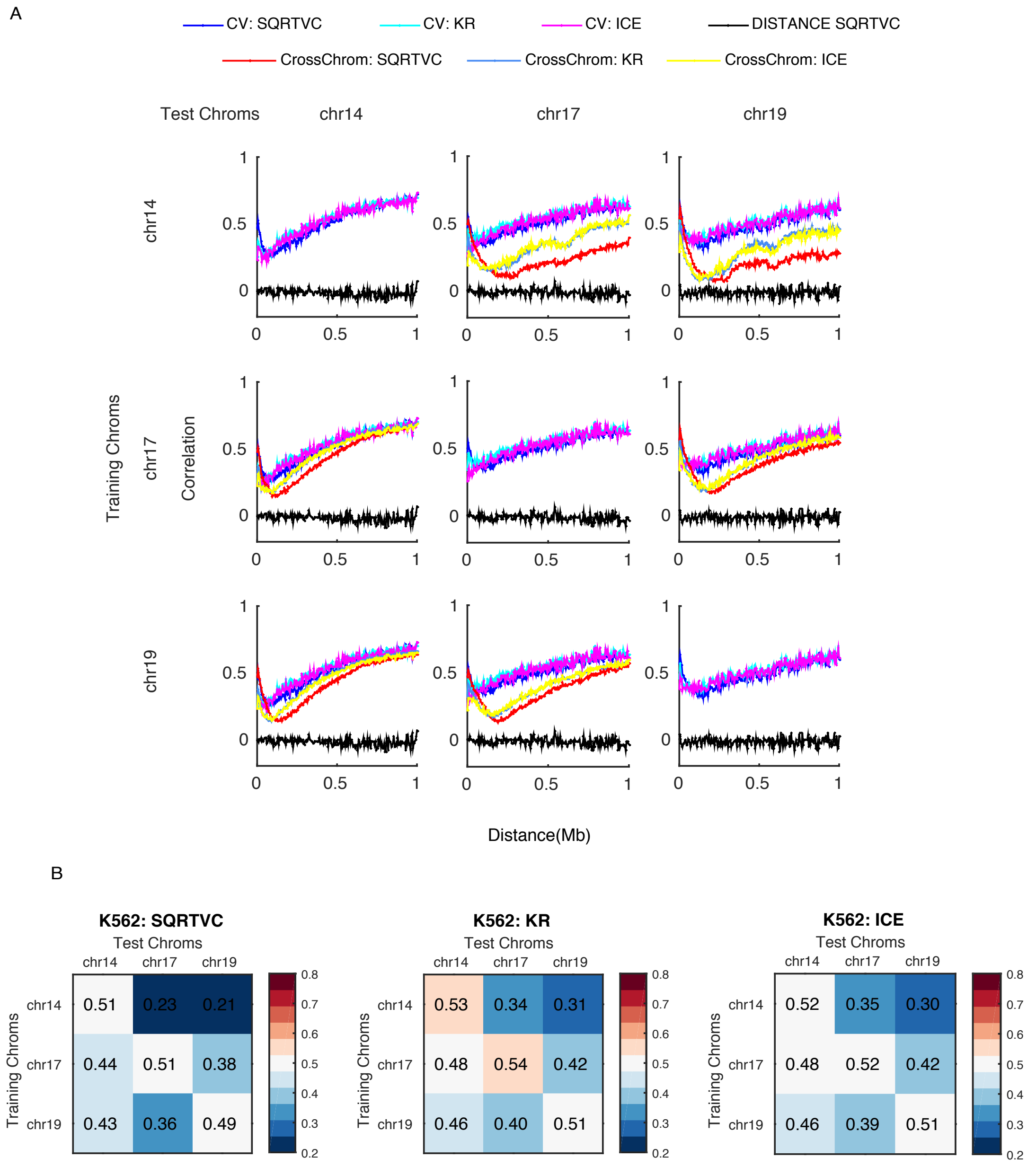

Supplementary Fig 29. Cross-chromosome performance across three chromosomes in the K562 cell line based on WINDOW features when using Hi-C data from three different normalization methods: Square root of Vanilla coverage (SQRTVC), Iterative Correction and Eigen vector decomposition (ICE) and Knight-Ruiz (KR). **A.** Shown are the distance stratified Pearson's correlation curves when training on the row chromosome and testing on the column chromosome. The curves in the diagonal positions correspond to CV performance. The CV lines are the same in each column. **B.** Heatmap of Area under the distance stratified correlation curve (AUC) for the CV and cross-chromosome experiments across the three chromosomes in the K562 cell line.

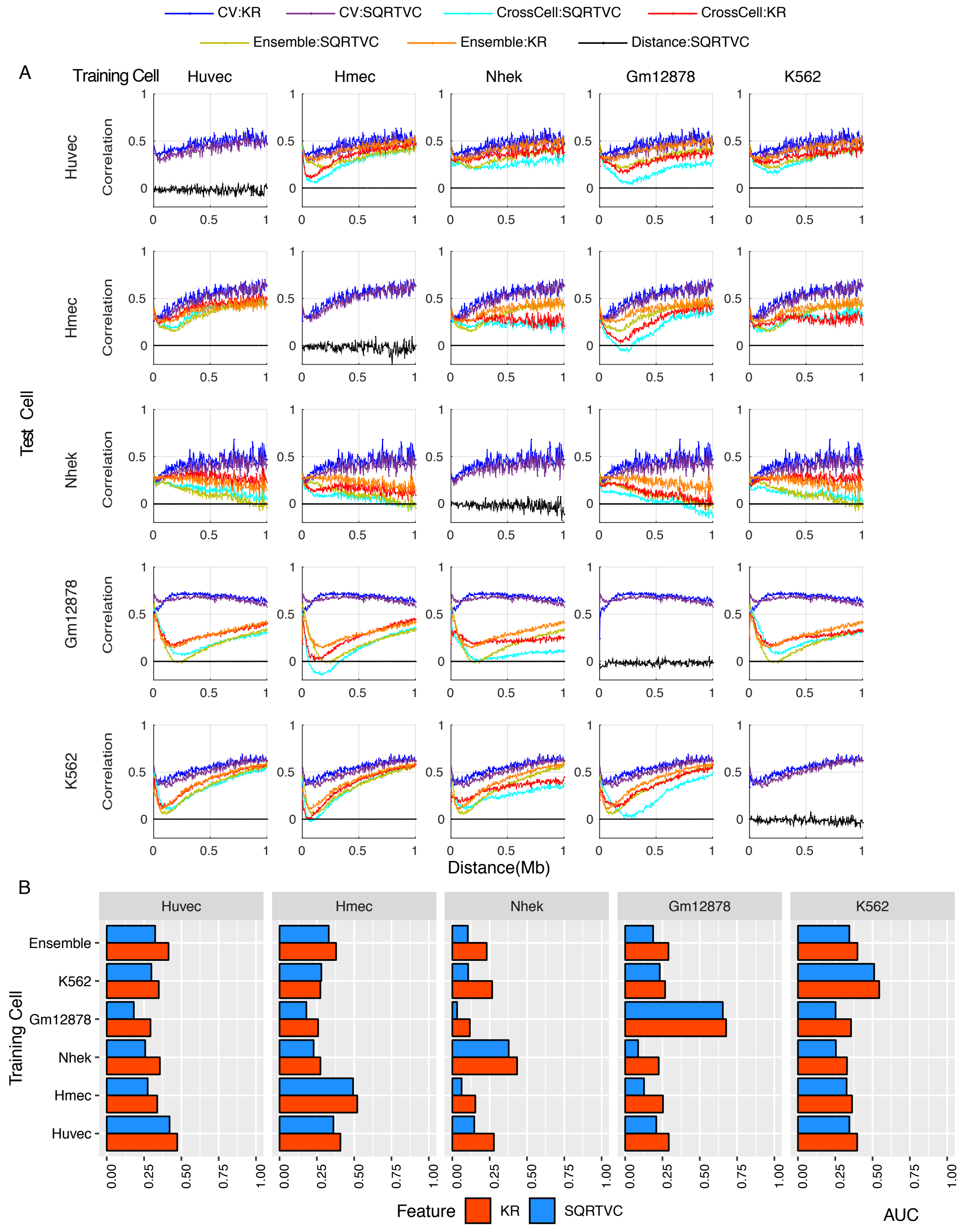

Supplementary Fig 30. Comparing cross-cell line performance based on WINDOW features using data normalized with the KR normalization versus the SQRTVC normalization on chromosome 17. **A.** Shown are distance stratified Pearson's correlation curve when training in the column cell line and testing in the row cell line. The diagonal plots show the same cell line cross-validation performance. The CV curves in each row are the same. **B.** Bar plots of AUCs for predicting KR normalized and SQRTVC normalized counts in each of the five cell lines. For each test cell line, the bar plots show the AUC obtained when training a model using either the SQRTVC or the KR normalized data or when using the Ensemble.

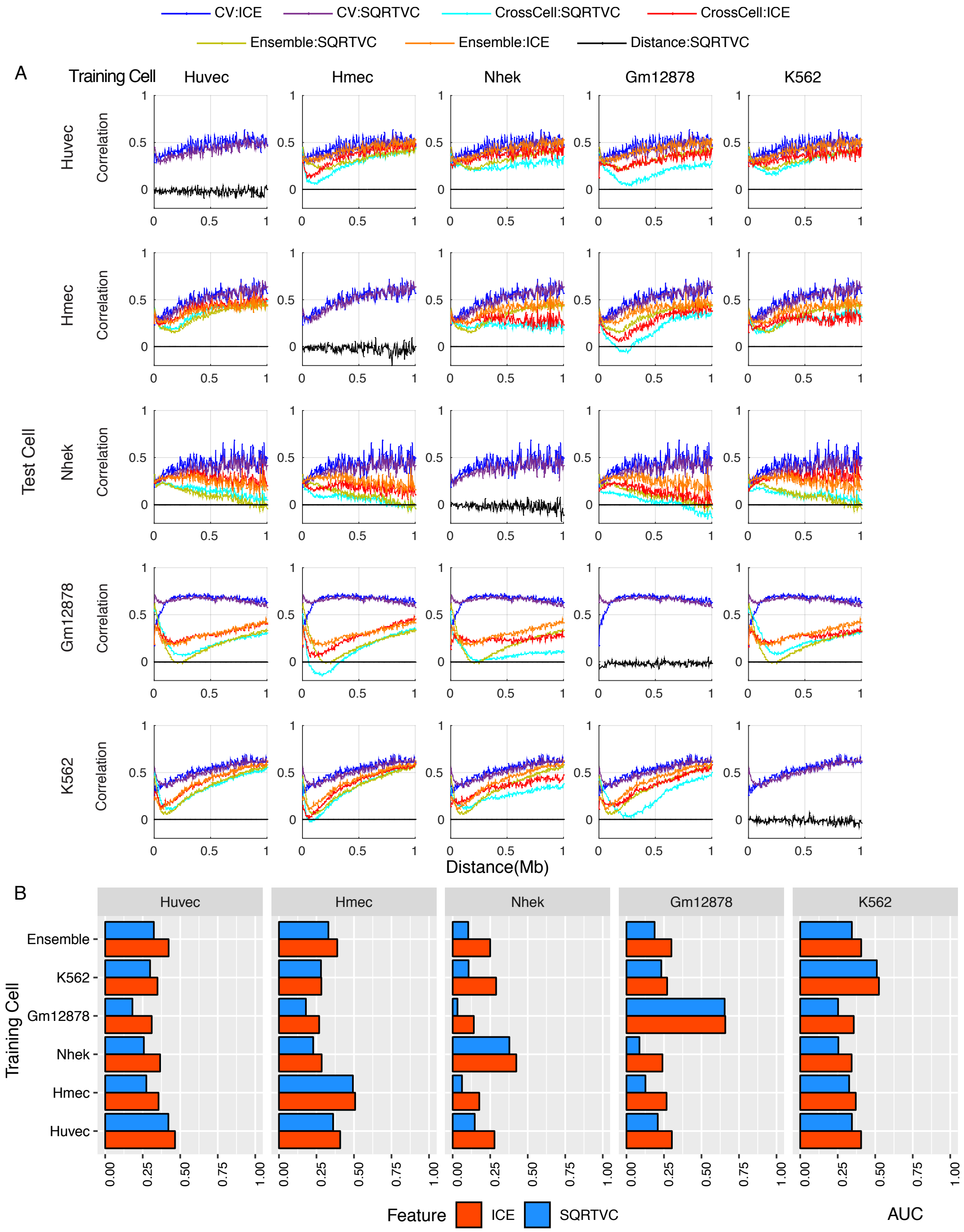

Supplementary Fig 31. Comparing cross-cell line performance based on WINDOW features using data normalized with the ICE normalization versus the SQRTVC normalization on chromosome 17. **A.** Shown are distance stratified Pearson's correlation curve when training in the column cell line and testing in the row cell line using data from the two normalization methods. The diagonal plots show the same cell line cross-validation performance. The CV curves in each row are the same. **B.** Bar plots of AUCs for predicting ICE normalized and SQRTVC normalized counts in each of the five test cell lines. For each test cell line, the bar plots show the AUC when training a model using either the SQRTVC or the ICE normalized data or when using the Ensemble.

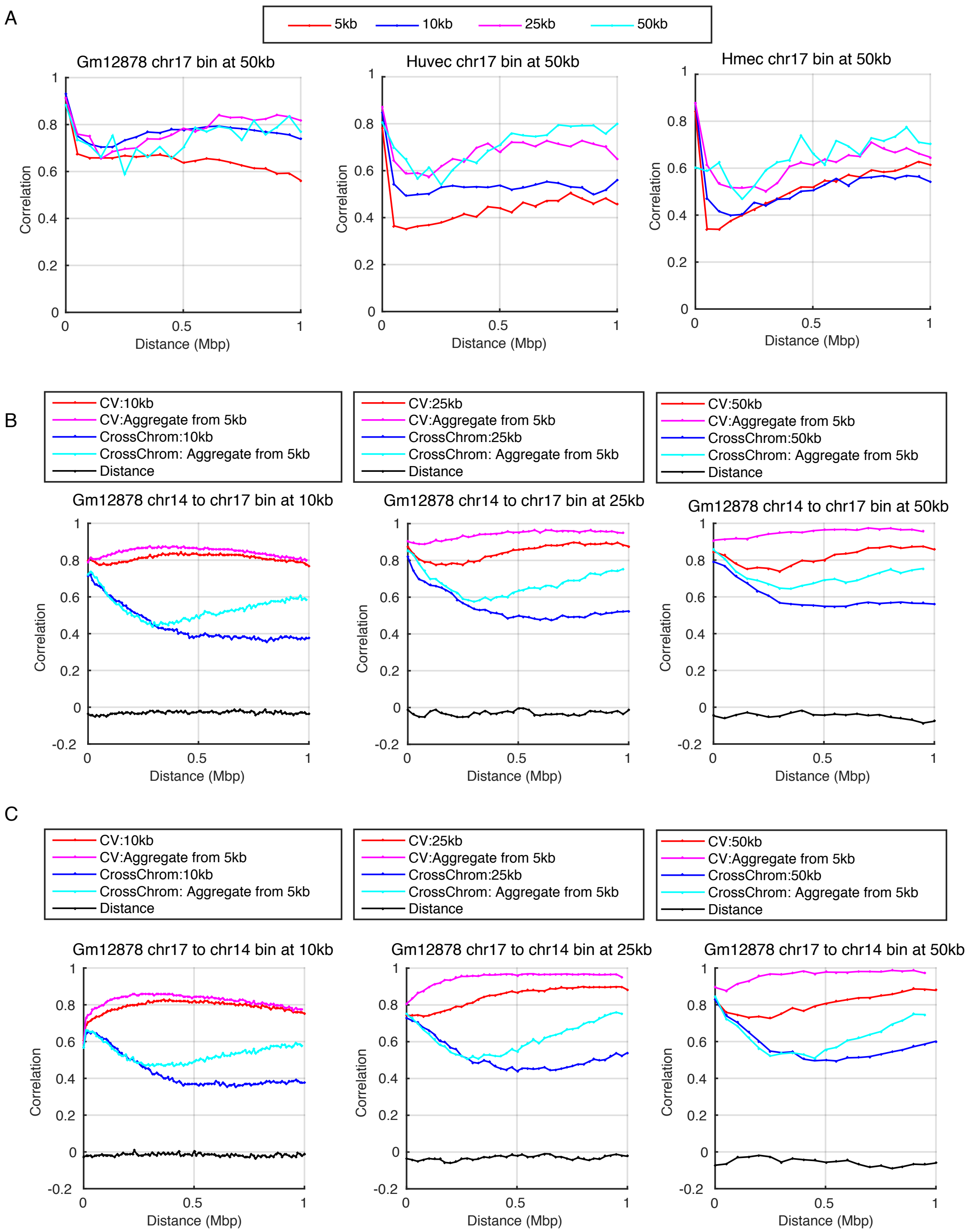

Supplementary Fig 32. Performance of HiC-Reg at different resolutions using data from the Gm12878 cell line. **A.** Shown are distance-stratified Pearson's correlation curves when training on data at different resolutions, 5kb, 10kb, 25kb and 50 kb. **B.** Cross-chromosome performance when training and testing at different resolutions. Here we also compare the performance of aggregating counts for larger bin sizes using a model trained at the highest resolution 5kb (magenta and cyan curves). At all resolutions, the training chromosome was chr14 and the test chromosome was chr17. **C.** Same as **B.** but using chr17 as the training chromosome and chr14 as the test chromosome.

Supplementary Tables:

Cell	Total Pairs	Significant True Count (FitHiC)	Significant True Count (Duan)	# Intersection	# Union	Jaccard Index	p-value
Gm12878	94899261	513002	719668	509545	723125	0.705	0
K562	63240643	3277992	1944205	1944197	3278000	0.593	0
Huvec	46160033	6946377	1902593	1902593	6946377	0.274	0
Hmec	35665655	9976366	1914586	1914586	9976366	0.192	0
Nhek	46035999	4795236	1389321	1389321	4795236	0.290	0

Supplementary Table 1. The Jaccard Index comparing the significant interactions identified from Fit-Hi-C and Duan et al with a cutoff of q-value <0.05, when applied to true counts.

Cell	Total Pairs	Top%	Number of top % pairs	# Intersection	# Union	Jaccard Index	Odds Ratio	p-value
Gm12878	94899261	1%	948993	808531	1089455	0.742	3844.388	0
Gm12878	94899261	5%	4744963	2918820	6571106	0.444	77.310	0
Gm12878	94899261	10%	9489926	3553381	15426471	0.230	8.013	0
K562	63240643	1%	632406	305445	959367	0.318	177.950	0
K562	63240643	5%	3162032	2469151	3854913	0.641	305.431	0
K562	63240643	10%	6324064	5340910	7307218	0.731	309.061	0
Huvec	46160033	1%	461600	146391	776809	0.188	66.867	0
Huvec	46160033	5%	2308002	1453035	3162969	0.459	85.470	0
Huvec	46160033	10%	4616003	3323992	5908014	0.563	80.152	0
Hmec	35665655	1%	356657	95846	617468	0.155	49.384	0
Hmec	35665655	5%	1783283	928757	2637809	0.352	42.008	0
Hmec	35665655	10%	3566566	2221009	4912123	0.452	37.726	0
Nhek	46035999	1%	460360	176432	744288	0.237	99.124	0
Nhek	46035999	5%	2301800	1444535	3159065	0.457	84.279	0
Nhek	46035999	10%	4603600	3195604	6011596	0.532	64.517	0

Supplementary Table 2. The Jaccard Index comparing the top 1%, 5% and 10% interactions ranked by significance identified from Fit-Hi-C and Duan et al, when applied to true counts.

Cell	chr	Number of significant interactions from Predictions	Number of Significant interactions from true count	Intersection	Union	Jaccard Index	Precision	Recall
Gm12878	9	815	13700	753	13762	0.055	0.924	0.055
Hmec	9	154240	422627	119013	457854	0.260	0.772	0.282
Nhek	9	1933	195444	1828	195549	0.009	0.946	0.009
K562	9	2408	113512	2135	113785	0.019	0.887	0.019
Huvec	9	4570	295038	4067	295541	0.014	0.890	0.014
Gm12878	14	832	13167	784	13215	0.059	0.942	0.060
Hmec	14	191541	343004	144213	390332	0.369	0.753	0.420
Nhek	14	1843	159381	1664	159560	0.010	0.903	0.010
K562	14	364	55757	333	55788	0.006	0.915	0.006
Huvec	14	4535	235446	4094	235887	0.017	0.903	0.017
Gm12878	16	419	20432	401	20450	0.020	0.957	0.020
Hmec	16	173158	300271	128341	345088	0.372	0.741	0.427
Nhek	16	9084	153338	8319	154103	0.054	0.916	0.054
K562	16	6786	107564	5824	108526	0.054	0.858	0.054
Huvec	16	17481	225226	14940	227767	0.066	0.855	0.066
Gm12878	17	920	23058	885	23093	0.038	0.962	0.038
Hmec	17	159791	309982	118994	350779	0.339	0.745	0.384
Nhek	17	5376	138574	4990	138960	0.036	0.928	0.036
K562	17	2082	102645	1913	102814	0.019	0.919	0.019
Huvec	17	12317	231923	10685	233555	0.046	0.868	0.046
Gm12878	19	196	12810	192	12814	0.015	0.980	0.015
Hmec	19	122321	217936	94965	245292	0.387	0.776	0.436
Nhek	19	402	79029	387	79044	0.005	0.963	0.005
K562	19	1282	76865	1131	77016	0.015	0.882	0.015
Huvec	19	15031	157838	13095	159774	0.082	0.871	0.083

Supplementary Table 3. The overlap of Fit-Hi-C interactions selected at a cutoff of q-value <0.05 called on true and predicted interaction counts for selected chromosomes. These chromosomes were selected because they had the well-characterized loci for long-range interactions.

Cell	chr	Number of pairs (top 5%)	Intersection	Union	Jaccard index	Precision	Recall	p-value (Hypergeometric)
Gm12878	9	197208	128697	265719	0.484	0.653	0.653	0
Hmec	9	76250	17369	135131	0.129	0.228	0.228	0
Nhek	9	96077	12163	179991	0.068	0.127	0.127	0
K562	9	114358	33542	195174	0.172	0.293	0.293	0
Huvec	9	97643	21894	173392	0.126	0.224	0.224	0
Gm12878	14	158786	11603	305969	0.038	0.073	0.073	0
Hmec	14	55899	16499	95299	0.173	0.295	0.295	0
Nhek	14	75927	10621	141233	0.075	0.140	0.140	0
K562	14	90592	7790	173394	0.045	0.086	0.086	0
Huvec	14	76136	16689	135583	0.123	0.219	0.219	0
Gm12878	16	132499	24909	240089	0.104	0.188	0.188	0
Hmec	16	47184	13184	81184	0.162	0.279	0.279	0
Nhek	16	59970	15563	104377	0.149	0.260	0.260	0
K562	16	95757	34290	157224	0.218	0.358	0.358	0
Huvec	16	64836	18404	111268	0.165	0.284	0.284	0
Gm12878	17	137415	18808	256022	0.073	0.137	0.137	0
Hmec	17	50710	13607	87813	0.155	0.268	0.268	0
Nhek	17	57238	13651	100825	0.135	0.238	0.238	0
K562	17	96698	27860	165536	0.168	0.288	0.288	0
Huvec	17	65862	16487	115237	0.143	0.250	0.250	0
Gm12878	19	101094	9516	192672	0.049	0.094	0.094	0
Hmec	19	34695	12431	56959	0.218	0.358	0.358	0
Nhek	19	39129	6270	71988	0.087	0.160	0.160	0
K562	19	74939	22215	127663	0.174	0.296	0.296	0
Huvec	19	46592	11337	81847	0.139	0.243	0.243	0

Supplementary Table 4. The overlap of top 5% interactions ranked using Fit-Hi-C q-values on the true and predicted counts for selected chromosomes. These chromosomes were selected because they had the well-characterized loci for long-range interactions.

Cell	Total Pairs	Significant pairs (SQRTVC)	Significant pairs (ICE)	Intersect ion	Union	Jaccard Index	Odds Ratio	p-value
Gm12878	94899261	513002	5321096	503742	5330356	0.095	1011.453	0
K562	63240643	3277992	1336005	1087661	3526336	0.308	119.401	0
Huvec	46160033	6946377	354193	334969	6965601	0.048	103.298	0
Hmec	35665655	9976366	336625	329306	9983685	0.033	119.779	0
Nhek	46035999	4795236	249043	228117	4816162	0.047	98.386	0

Supplementary Table 5. The overlap of Fit-Hi-C interactions selected at a cutoff of q-value <0.05 between ICE and SQRTVC normalized counts

Cell	Top %	Total Pairs	Number of top % pairs	Intersection	Union	Jaccard Index	Odds Ratio	p-value (Hypergeometric)
Gm12878	1%	94899261	948993	590142	1307844	0.451	428.908	0
Gm12878	5%	94899261	4744963	2628605	6861321	0.383	51.667	0
Gm12878	10%	94899261	9489926	3485661	15494191	0.225	7.677	0
K562	1%	63240643	632406	325479	939333	0.347	215.253	0
K562	5%	63240643	3162032	2154186	4169878	0.517	125.276	0
K562	10%	63240643	6324064	4635402	8012726	0.579	89.776	0
Huvec	1%	46160033	461600	229286	693914	0.330	193.159	0
Huvec	5%	46160033	2308002	1538697	3077307	0.500	112.011	0
Huvec	10%	46160033	4616003	2946460	6285546	0.469	42.150	0
Hmec	1%	35665655	356657	197540	515774	0.383	274.249	0
Hmec	5%	35665655	1783283	1204355	2362211	0.510	119.673	0
Hmec	10%	35665655	3566566	2592538	4540594	0.571	85.054	0
Nhek	1%	46035999	460360	269297	651423	0.413	334.801	0
Nhek	5%	46035999	2301800	1662878	2940722	0.565	175.547	0
Nhek	10%	46035999	4603600	2456374	6750826	0.364	20.930	0

Supplementary Table 6. The overlap of top 1%, 5%, 10% interactions ranked using Fit-Hi-C q-value scores between ICE and SQRTVC normalized counts

HBA1	chr16_220000_225000				chr16_225000_230000			
Prediction setting	Predicted Count		True Count		Predicted Count		True Count	
	# significant (Fit-Hi-C)	# overlap 5C	# significant (Fit-Hi-C)	# overlap 5C	# significant (Fit-Hi-C)	# overlap 5C	# significant (Fit-Hi-C)	# overlap 5C
Gm12878 CV	0	0	3	1	0	0	15	2
K562 CV	2	2	20	2	2	0	35	4
Huvec CV	0	0	38	2	0	0	37	3
Hmec CV	12	0	32	0	14	0	44	4
Nhek CV	2	0	25	2	0	0	35	1
K562toGm12878	0	0	NA		0	0	NA	
HuvectoGm12878	0	0	NA		1	0	NA	
HmectoGm12878	1	0	NA		2	0	NA	
NhektoGm12878	0	0	NA		1	0	NA	
Gm12878toK562	0	0	NA		0	0	NA	
HuvectoK562	0	0	NA		0	0	NA	
HmectoK562	0	0	NA		0	0	NA	
NhektoK562	0	0	NA		0	0	NA	
Gm12878toHuvec	0	0	NA		0	0	NA	
K562toHuvec	0	0	NA		0	0	NA	
HmectoHuvec	10	0	NA		5	0	NA	
NhektoHuvec	0	0	NA		0	0	NA	
Gm12878toHmec	0	0	NA		0	0	NA	
K562toHmec	0	0	NA		0	0	NA	
HuvectoHmec	10	0	NA		8	0	NA	
NhektoHmec	13	0	NA		4	0	NA	
Gm12878toNhek	0	0	NA		0	0	NA	
K562toNhek	0	0	NA		0	0	NA	
HuvectoNhek	1	0	NA		1	0	NA	
HmectoNhek	1	0	NA		7	0	NA	
Ensemble-Gm12878	0	0	NA		0	0	NA	
Ensemble-K562	0	0	NA		0	0	NA	
Ensemble-Huvec	0	0	NA		0	0	NA	
Ensemble-Hmec	0	0	NA		0	0	NA	
Ensemble-Nhek	0	0	NA		0	0	NA	

Supplementary Table 7. Number of significant interactions associated with *HBA1* gene and how many of them overlapped with 5C dataset identified by Fit-Hi-C in different prediction settings and true counts.

PAPPA gene	chr9_118910000_118915000						chr9_118915000_118920000					
	Predicted Count			True Count			Predicted Count			True Count		
Prediction setting	# significant (Fit-Hi-C)	MCS5C	TCE	# significant (Fit-Hi-C)	MCS5C	TCE	# significant (Fit-Hi-C)	MCS5C	TCE	# significant (Fit-Hi-C)	MCS5C	TCE
Gm12878 CV	0	0	0	4	0	0	0	0	0	1	0	0
K562 CV	0	0	0	22	2	1	0	0	0	16	0	0
Huvec CV	0	0	0	28	11	3	0	0	0	12	2	1
Hmec CV	4	2	2	70	10	2	1	0	0	45	6	1
Nhek CV	0	0	0	37	8	3	0	0	0	33	3	1
K562toGm12878	0	0	0	NA			0	0	0	NA		
HuvectoGm12878	0	0	0	NA			0	0	0	NA		
HmectoGm12878	0	0	0	NA			0	0	0	NA		
NhektoGm12878	0	0	0	NA			0	0	0	NA		
Gm12878toK562	0	0	0	NA			0	0	0	NA		
HuvectoK562	0	0	0	NA			0	0	0	NA		
HmectoK562	23	1	0	NA			14	0	0	NA		
NhektoK562	0	0	0	NA			0	0	0	NA		
Gm12878toHuvec	0	0	0	NA			0	0	0	NA		
K562toHuvec	0	0	0	NA			0	0	0	NA		
HmectoHuvec	58	23	3	NA			33	14	3	NA		
NhektoHuvec	0	0	0	NA			0	0	0	NA		
Gm12878toHmec	0	0	0	NA			0	0	0	NA		
K562toHmec	0	0	0	NA			0	0	0	NA		
HuvectoHmec	0	0	0	NA			0	0	0	NA		
NhektoHmec	0	0	0	NA			0	0	0	NA		
Gm12878toNhek	0	0	0	NA			0	0	0	NA		
K562toNhek	0	0	0	NA			0	0	0	NA		
HuvectoNhek	0	0	0	NA			0	0	0	NA		
HmectoNhek	9	0	0	NA			3	0	0	NA		
Ensemble-Gm12878	0	0	0	NA			0	0	0	NA		
Ensemble-K562	0	0	0	NA			0	0	0	NA		
Ensemble-Huvec	0	0	0	NA			0	0	0	NA		
Ensemble-Hmec	0	0	0	NA			0	0	0	NA		
Ensemble-Nhek	0	0	0	NA			0	0	0	NA		

Supplementary Table 8. Number of significant interactions associated with *PAPPA* gene and how many of them overlapped with MCS5C region and TCE region identified by Fit-Hi-C in different prediction settings and true counts
