# Supplementary material for: In silico prediction of high-resolution Hi-C interaction matrices
Source: Nat Commun. 2019 Dec 6;10:5449. doi: 10.1038/s41467-019-13423-8 (PMC6898380; doi:10.1038/s41467-019-13423-8)
Supplement: Supplementary file 2 — Reporting Summary [file 41467_2019_13423_MOESM2_ESM.pdf]

nature research

Corresponding author(s): Sushmita Roy

Last updated by author(s): Nov 4, 2019

Reporting Summary

Nature Research wishes to improve the reproducibility of the work that we publish. This form provides structure for consistency and transparency in reporting. For further information on Nature Research policies, see Authors & Referees and the Editorial Policy Checklist .

Statistics

For all statistical analyses, confirm that the following items are present in the figure legend, table legend, main text, or Methods section.

n/a	Confirmed	
☐	☒	The exact sample size (n) for each experimental group/condition, given as a discrete number and unit of measurement
☒	☐	A statement on whether measurements were taken from distinct samples or whether the same sample was measured repeatedly
☐	☒	The statistical test(s) used AND whether they are one- or two-sided<br>*Only common tests should be described solely by name; describe more complex techniques in the Methods section.*
☒	☐	A description of all covariates tested
☐	☒	A description of any assumptions or corrections, such as tests of normality and adjustment for multiple comparisons
☐	☒	A full description of the statistical parameters including central tendency (e.g. means) or other basic estimates (e.g. regression coefficient) AND variation (e.g. standard deviation) or associated estimates of uncertainty (e.g. confidence intervals)
☐	☒	For null hypothesis testing, the test statistic (e.g. F, t, r) with confidence intervals, effect sizes, degrees of freedom and P value noted<br>*Give P values as exact values whenever suitable.*
☒	☐	For Bayesian analysis, information on the choice of priors and Markov chain Monte Carlo settings
☒	☐	For hierarchical and complex designs, identification of the appropriate level for tests and full reporting of outcomes
☐	☒	Estimates of effect sizes (e.g. Cohen's d, Pearson's r), indicating how they were calculated

Our web collection on statistics for biologists contains articles on many of the points above.

Software and code

Policy information about availability of computer code

Data collection	Hi-C SQRTVC normalized contact counts in five cell lines are downloaded from Rao et al (GEO: GSE63525). ChIP-seq datasets of histone marks in five cell lines are downloaded from http://hgdownload.cse.ucsc.edu/goldenPath/hg19/encodeDCC/wgEncodeBroadHistone/. DNase I-seq datasets in five cell lines are downloaded from http://hgdownload.cse.ucsc.edu/goldenPath/hg19/encodeDCC/wgEncodeOpenChromDnase/.
Data analysis	We obtained the raw fastq files of ChIP-seq and DNase I-seq datasets from the ENCODE consortium, aligned reads to the human hg19 assembly using bowtie2 (bowtie2-2.2.0), retrieved reads aligned to a locus using SAMtools (samtools-1.3) and applied BEDTools (bedtools-2.17.0) to obtain a base pair level read count. We next aggregated the read counts of each base pair in a 5kb region using our custom code. Next, we normalized aggregated signal by sequencing depth and collapsed replicates by taking the median using our custom code. As TBP and RAD21 ChIP-seq data are not available in Huvec, Nhek and Hmec cell lines, we predicted the binding sites using PIQ (http://piq.csail.mit.edu/) on the DNase I data and used the sum of purity scores for all motifs mapped to the same 5kb bin as the signal value.<br>We used our custom scripts to generate training and test data, train HiC-Reg models, make predictions and conduct feature analysis. Our custom scripts are available at https://github.com/Roy-lab/HiC-Reg.<br>We used Fit-Hi-C (https://github.com/ay-lab/fithic) to call significant pairs of interactions. We applied the Hi-C Domain Caller program in Dixon et al (http://bioinformatics-renlab.ucsd.edu/collaborations/sid/domaincall_software.zip) to call TADs for HiC-Reg predicted counts and true counts. For feature selection with Multi-task Group-LASSO, we used the implementation of this regression framework (mtLeastR function) in the Sparse Learning with Efficient Projections package for MATLAB (SLEP v4.1, https://github.com/jiayuzhou/SLEP).

For manuscripts utilizing custom algorithms or software that are central to the research but not yet described in published literature, software must be made available to editors/reviewers. We strongly encourage code deposition in a community repository (e.g. GitHub). See the Nature Research guidelines for submitting code & software for further information.

Data

Policy information about availability of data

All manuscripts must include a data availability statement. This statement should provide the following information, where applicable:

- Accession codes, unique identifiers, or web links for publicly available datasets
- A list of figures that have associated raw data
- A description of any restrictions on data availability

The datasets generated and analysed by the current study are available at http://doi.org/10.5281/zenodo.3525432 (part I), https://doi.org/10.5281/zenodo.3525510 (part II) and https://doi.org/10.5281/zenodo.3525514 (part III).
The associated data for Figs. 5A-C, 7A-C are provided as a Source Data file. The associated data underlying Figs. 2, 3, 6, 7 and 8A, C and Supplementary Figs 1-10, 19, 20, 22, 24-26 are provided at https://doi.org/10.5281/zenodo.3525514.

Field-specific reporting

Please select the one below that is the best fit for your research. If you are not sure, read the appropriate sections before making your selection.

[x] Life sciences [ ] Behavioural & social sciences [ ] Ecological, evolutionary & environmental sciences

For a reference copy of the document with all sections, see nature.com/documents/nr-reporting-summary-flat.pdf

Life sciences study design

All studies must disclose on these points even when the disclosure is negative.

Sample size	Our training and testing were done for each cell line using 5-fold cross validation. The sample size for 5-fold cross-validation is determined by the number of pairs available in Hi-C data for each cell line.
Data exclusions	We included all relevant data for our analysis.
Replication	We have provided code, scripts, inputs and outputs from our experiments to enable replication of our study.
Randomization	Training/testing datasets were generated by randomly partitioning regions into non-overlapping sets and considering measured interactions among the regions.
Blinding	n/a

Reporting for specific materials, systems and methods

We require information from authors about some types of materials, experimental systems and methods used in many studies. Here, indicate whether each material, system or method listed is relevant to your study. If you are not sure if a list item applies to your research, read the appropriate section before selecting a response.

Materials & experimental systems

n/a	Involved in the study	
[x]	[ ]	Antibodies
[x]	[ ]	Eukaryotic cell lines
[x]	[ ]	Palaeontology
[x]	[ ]	Animals and other organisms
[x]	[ ]	Human research participants
[x]	[ ]	Clinical data

Methods

n/a	Involved in the study	
[x]	[ ]	ChIP-seq
[x]	[ ]	Flow cytometry
[x]	[ ]	MRI-based neuroimaging
