# Supplementary material for: In silico prediction of high-resolution Hi-C interaction matrices
Source: Nat Commun. 2019 Dec 6;10:5449. doi: 10.1038/s41467-019-13423-8 (PMC6898380; doi:10.1038/s41467-019-13423-8)
Supplement: Supplementary file 3 — Description of Additional Supplementary Files [file 41467_2019_13423_MOESM3_ESM.pdf]

Description of Additional Supplementary Files

File Name: Supplementary Data 1
Description: Memory usage and run time of HiC-Reg for MULTI-CELL and WINDOW features per chromosome and genome-wide.
